# Supplementary material for: Social and health system factors associated with maternal mortality in Eastern and Western China: Population health estimates using provincial-level data
Source: PLoS Med. 2025 Dec 4;22(12):e1004837. doi: 10.1371/journal.pmed.1004837 (PMC12677549; doi:10.1371/journal.pmed.1004837)
Supplement: S1 Table — Note: Ob/Gyn, obstetrics and gynecology. (DOCX) [file pmed.1004837.s001.docx]

**Table S1 Social and health system-related factors of maternal mortality.**

| Factors cited from Souza and colleagues’ review | Relevant variables in available data source |
| --- | --- |
| *Social determinants* | |
| - Low income and low socioeconomic status | - Per capita disposable income |
| - Low maternal education | - Average years of schooling for females |
| - Living in a rural area | - Urbanization rate |
| - Gender dynamics that favor sexism and gender injustice (inequity and inequality) - Ethnic and racial dynamics that favor racism and discrimination - Sociocultural factors that sustain gender and social bias against women, including but not limited to gender roles, and restricted agency over sexual and reproductive rights - Exposure to sources of hyperinformation and disinformation - Hunger - Corruption - Armed conflict - Violence (including but not limited to intimate partner violence) | N.A. |
| *Health services and health education* | |
| - Low knowledge of danger signs related to obstetric complication (first delay) | - Average years of schooling for females - Per capita disposable income |
| - Absence of agency and autonomy for health-care seeking (first delay) | - Prenatal booking rate - Antenatal care rate - Hospital delivery rate - Local fiscal expenditure on healthcare - Urbanization rate - Per capita disposable income - Number of hospital beds for Ob/Gyn per 1000 births - Number of health technical personnel in maternal and child health care per 1000 births |
| - Poor access to health services (second delay; includes no antenatal care visits and long distance to a health facility) | - Prenatal booking rate - Antenatal care rate - Hospital delivery rate - Local fiscal expenditure on healthcare - Urbanization rate - Per capita disposable income |
| - Substandard care (third delay) | - Local fiscal expenditure on healthcare - Number of hospital beds for Ob/Gyn per 1000 births - Number of health technical personnel in maternal and child health care per 1000 births |

Note: Ob/Gyn, obstetrics and gynecology.
