# Supplementary material for: Social and health system factors associated with maternal mortality in Eastern and Western China: Population health estimates using provincial-level data
Source: PLoS Med. 2025 Dec 4;22(12):e1004837. doi: 10.1371/journal.pmed.1004837 (PMC12677549; doi:10.1371/journal.pmed.1004837)
Supplement: S2 Table — (DOCX) [file pmed.1004837.s002.docx]

**Table S2 Cause-specific maternal mortality rate in China, 2004-2020.**

| **Year** | **Maternal hemorrhage** | **Indirect maternal deaths** | **Other direct maternal disorders** | **Maternal hypertensive disorders** | **Maternal obstructed labor and uterine rupture** | **Late maternal deaths** | **Ectopic pregnancy** | **Maternal sepsis and other maternal infections** | **Maternal abortion and miscarriage** | **Maternal deaths aggravated by HIV/AIDS** |
| --- | --- | --- | --- | --- | --- | --- | --- | --- | --- | --- |
| 2004 | 0.34 | 0.31 | 0.14 | 0.13 | 0.06 | 0.03 | 0.03 | 0.03 | 0.01 | 0.00 |
| 2005 | 0.29 | 0.28 | 0.13 | 0.12 | 0.05 | 0.03 | 0.03 | 0.03 | 0.01 | 0.00 |
| 2006 | 0.25 | 0.25 | 0.12 | 0.10 | 0.05 | 0.03 | 0.03 | 0.02 | 0.01 | 0.00 |
| 2007 | 0.21 | 0.23 | 0.11 | 0.09 | 0.04 | 0.03 | 0.03 | 0.02 | 0.01 | 0.00 |
| 2008 | 0.19 | 0.21 | 0.10 | 0.09 | 0.04 | 0.03 | 0.03 | 0.02 | 0.01 | 0.00 |
| 2009 | 0.16 | 0.19 | 0.10 | 0.08 | 0.03 | 0.03 | 0.03 | 0.01 | 0.01 | 0.00 |
| 2010 | 0.13 | 0.16 | 0.08 | 0.06 | 0.03 | 0.03 | 0.02 | 0.01 | 0.01 | 0.00 |
| 2011 | 0.11 | 0.14 | 0.07 | 0.06 | 0.02 | 0.03 | 0.02 | 0.01 | 0.01 | 0.00 |
| 2012 | 0.10 | 0.13 | 0.07 | 0.05 | 0.02 | 0.03 | 0.02 | 0.01 | 0.01 | 0.00 |
| 2013 | 0.09 | 0.12 | 0.06 | 0.05 | 0.02 | 0.03 | 0.02 | 0.01 | 0.01 | 0.00 |
| 2014 | 0.08 | 0.11 | 0.06 | 0.05 | 0.02 | 0.02 | 0.02 | 0.01 | 0.01 | 0.00 |
| 2015 | 0.08 | 0.11 | 0.06 | 0.05 | 0.02 | 0.03 | 0.02 | 0.01 | 0.01 | 0.00 |
| 2016 | 0.08 | 0.11 | 0.06 | 0.05 | 0.02 | 0.03 | 0.02 | 0.01 | 0.01 | 0.00 |
| 2017 | 0.07 | 0.10 | 0.06 | 0.04 | 0.02 | 0.03 | 0.02 | 0.01 | 0.00 | 0.00 |
| 2018 | 0.06 | 0.09 | 0.05 | 0.04 | 0.02 | 0.02 | 0.02 | 0.01 | 0.00 | 0.00 |
| 2019 | 0.05 | 0.08 | 0.05 | 0.03 | 0.02 | 0.02 | 0.01 | 0.01 | 0.00 | 0.00 |
| 2020 | 0.04 | 0.07 | 0.04 | 0.03 | 0.01 | 0.02 | 0.01 | 0.00 | 0.00 | 0.00 |
