# Supplementary material for: Social and health system factors associated with maternal mortality in Eastern and Western China: Population health estimates using provincial-level data
Source: PLoS Med. 2025 Dec 4;22(12):e1004837. doi: 10.1371/journal.pmed.1004837 (PMC12677549; doi:10.1371/journal.pmed.1004837)
Supplement: S3 Table — (DOCX) [file pmed.1004837.s003.docx]

**Table S3 Cause-specific maternal mortality fractions in China, 2004-2020.**

| **Year** | **Maternal hemorrhage** | **Indirect maternal deaths** | **Other direct maternal disorders** | **Maternal hypertensive disorders** | **Maternal obstructed labor and uterine rupture** | **Late maternal deaths** | **Ectopic pregnancy** | **Maternal sepsis and other maternal infections** | **Maternal abortion and miscarriage** | **Maternal deaths aggravated by HIV/AIDS** |
| --- | --- | --- | --- | --- | --- | --- | --- | --- | --- | --- |
| 2004 | 31.2 | 28.2 | 13.2 | 11.9 | 5.2 | 3.2 | 3.1 | 2.8 | 1.3 | 0.0 |
| 2005 | 29.9 | 28.5 | 13.7 | 11.9 | 5.2 | 3.5 | 3.3 | 2.7 | 1.3 | 0.0 |
| 2006 | 28.8 | 28.7 | 14.1 | 12.0 | 5.2 | 3.8 | 3.5 | 2.5 | 1.4 | 0.0 |
| 2007 | 27.5 | 29.1 | 14.5 | 12.1 | 5.2 | 4.1 | 3.7 | 2.4 | 1.4 | 0.0 |
| 2008 | 26.4 | 29.5 | 14.8 | 12.1 | 5.1 | 4.5 | 3.9 | 2.3 | 1.4 | 0.0 |
| 2009 | 25.0 | 29.9 | 15.1 | 12.2 | 5.1 | 4.9 | 4.1 | 2.3 | 1.4 | 0.0 |
| 2010 | 24.1 | 30.1 | 15.3 | 12.2 | 5.1 | 5.2 | 4.3 | 2.2 | 1.4 | 0.0 |
| 2011 | 23.4 | 30.2 | 15.5 | 12.2 | 5.2 | 5.6 | 4.4 | 2.2 | 1.4 | 0.0 |
| 2012 | 22.7 | 30.1 | 15.7 | 12.2 | 5.4 | 5.9 | 4.4 | 2.2 | 1.4 | 0.0 |
| 2013 | 22.0 | 30.0 | 15.9 | 12.2 | 5.6 | 6.2 | 4.5 | 2.2 | 1.4 | 0.0 |
| 2014 | 21.3 | 30.0 | 16.1 | 12.2 | 5.8 | 6.6 | 4.6 | 2.1 | 1.4 | 0.0 |
| 2015 | 20.7 | 30.0 | 16.2 | 12.2 | 5.9 | 6.9 | 4.7 | 2.1 | 1.4 | 0.0 |
| 2016 | 20.2 | 29.9 | 16.4 | 12.2 | 6.0 | 7.2 | 4.7 | 2.1 | 1.4 | 0.0 |
| 2017 | 19.5 | 30.0 | 16.6 | 12.0 | 6.1 | 7.5 | 4.9 | 2.1 | 1.3 | 0.0 |
| 2018 | 18.9 | 30.1 | 16.9 | 12.0 | 6.2 | 7.9 | 4.9 | 2.0 | 1.3 | 0.0 |
| 2019 | 18.3 | 30.2 | 17.1 | 11.8 | 6.2 | 8.2 | 5.0 | 1.9 | 1.3 | 0.0 |
| 2020 | 17.8 | 30.3 | 17.3 | 11.6 | 6.3 | 8.6 | 5.0 | 1.8 | 1.3 | 0.0 |
