# Supplementary material for: Social and health system factors associated with maternal mortality in Eastern and Western China: Population health estimates using provincial-level data
Source: PLoS Med. 2025 Dec 4;22(12):e1004837. doi: 10.1371/journal.pmed.1004837 (PMC12677549; doi:10.1371/journal.pmed.1004837)
Supplement: S4 Table — Note: GroupPIP, group posterior inclusion probabilities; CondPIP, conditional posterior inclusion probabilities; MCH, maternal and child health; Ob/Gyn, obstetrics and gynecology; PCDI, per capita disposable income. (DOCX) [file pmed.1004837.s004.docx]

**Table S4 Group and conditional posterior inclusion probabilities for each factor in Eastern China, 2004-2012, using Bayesian Kernel Machine Regression hierarchical variable selection.**

| **Exposure** | **Exposure group** | **Total maternal mortality** | | **Maternal mortality due to hemorrhage** | | **Maternal mortality due to coexisting medical diseases** | | **Maternal mortality due to hypertensive disorders in pregnancy** | |
| --- | --- | --- | --- | --- | --- | --- | --- | --- | --- |
|  |  | **GroupPIP** | **CondPIP** | **GroupPIP** | **CondPIP** | **GroupPIP** | **CondPIP** | **GroupPIP** | **CondPIP** |
| Hospital delivery rate | 1 | 1 | 1 | 1 | 1 | 1 | 0.877 | 1 | 0.941 |
| Antenatal care rate | 1 | 1 | 0 | 1 | 0 | 1 | 0 | 1 | 0.059 |
| Prenatal booking rate | 1 | 1 | 0 | 1 | 0 | 1 | 0.123 | 1 | 0 |
| Local fiscal expenditure on healthcare | 2 | 1 | 1 | 1 | 1 | 0.874 | 1 | 0.917 | 1 |
| Urbanization rate | 3 | 1 | 1 | 1 | 1 | 0.995 | 0.864 | 0.665 | 0.410 |
| PCDI | 3 | 1 | 0 | 1 | 0 | 0.995 | 0.134 | 0.665 | 0.549 |
| Average years of schooling for females | 3 | 1 | 0 | 1 | 0 | 0.995 | 0.001 | 0.665 | 0.041 |
| Number of Ob/Gyn beds per 1000 livebirths | 4 | 0.818 | 0.103 | 0.998 | 0 | 0.642 | 0.734 | 0.487 | 0.889 |
| Number of MCH personnel per 1000 livebirths | 4 | 0.818 | 0.897 | 0.998 | 1 | 0.642 | 0.266 | 0.487 | 0.111 |

Note: GroupPIP, group posterior inclusion probabilities; CondPIP, conditional posterior inclusion probabilities; MCH, maternal and child health; Ob/Gyn, obstetrics and gynecology; PCDI, per capita disposable income.
