# Supplementary material for: Social and health system factors associated with maternal mortality in Eastern and Western China: Population health estimates using provincial-level data
Source: PLoS Med. 2025 Dec 4;22(12):e1004837. doi: 10.1371/journal.pmed.1004837 (PMC12677549; doi:10.1371/journal.pmed.1004837)
Supplement: S5 Table — Note: GroupPIP, group posterior inclusion probabilities; CondPIP, conditional posterior inclusion probabilities; MCH, maternal and child health; Ob/Gyn, obstetrics and gynecology; PCDI, per capita disposable income. (DOCX) [file pmed.1004837.s005.docx]

**Table S5 Group and conditional posterior inclusion probabilities for each factor in Western China, 2004-2012, using Bayesian Kernel Machine Regression hierarchical variable selection.**

| **Exposure** | **Exposure group** | **Total maternal mortality** | | **Maternal mortality due to hemorrhage** | | **Maternal mortality due to coexisting medical diseases** | | **Maternal mortality due to hypertensive disorders in pregnancy** | |
| --- | --- | --- | --- | --- | --- | --- | --- | --- | --- |
|  |  | **GroupPIP** | **CondPIP** | **GroupPIP** | **CondPIP** | **GroupPIP** | **CondPIP** | **GroupPIP** | **CondPIP** |
| Hospital delivery rate | 1 | 1 | 0.017 | 1 | 0.317 | 1 | 1 | 1 | 0 |
| Antenatal care rate | 1 | 1 | 0.967 | 1 | 0.623 | 1 | 0 | 1 | 1 |
| Prenatal booking rate | 1 | 1 | 0.016 | 1 | 0.061 | 1 | 0 | 1 | 0 |
| Local fiscal expenditure on healthcare | 2 | 0.348 | 1 | 0.221 | 1 | 0.411 | 1 | 0.024 | 1 |
| Urbanization rate | 3 | 1 | 0 | 0.857 | 0.005 | 0.564 | 0.269 | 0.075 | 0.094 |
| PCDI | 3 | 1 | 1 | 0.857 | 0.918 | 0.564 | 0.061 | 0.075 | 0.834 |
| Average years of schooling for females | 3 | 1 | 0 | 0.857 | 0.077 | 0.564 | 0.670 | 0.075 | 0.072 |
| Number of Ob/Gyn beds per 1000 livebirths | 4 | 0.652 | 0.481 | 0.293 | 0.432 | 0.323 | 0.457 | 0.032 | 0.285 |
| Number of MCH personnel per 1000 livebirths | 4 | 0.652 | 0.519 | 0.293 | 0.568 | 0.323 | 0.543 | 0.032 | 0.715 |

Note: GroupPIP, group posterior inclusion probabilities; CondPIP, conditional posterior inclusion probabilities; MCH, maternal and child health; Ob/Gyn, obstetrics and gynecology; PCDI, per capita disposable income.
