# Supplementary material for: Social and health system factors associated with maternal mortality in Eastern and Western China: Population health estimates using provincial-level data
Source: PLoS Med. 2025 Dec 4;22(12):e1004837. doi: 10.1371/journal.pmed.1004837 (PMC12677549; doi:10.1371/journal.pmed.1004837)
Supplement: S6 Table — Note: GroupPIP, group posterior inclusion probabilities; CondPIP, conditional posterior inclusion probabilities; MCH, maternal and child health; Ob/Gyn, obstetrics and gynecology; PCDI, per capita disposable income. (DOCX) [file pmed.1004837.s006.docx]

**Table S6 Group and conditional posterior inclusion probabilities for each factor in Eastern China, 2013-2020, using Bayesian Kernel Machine Regression hierarchical variable selection.**

| **Exposure** | **Exposure group** | **Total maternal mortality** | | **Maternal mortality due to hemorrhage** | | **Maternal mortality due to coexisting medical diseases** | | **Maternal mortality due to hypertensive disorders in pregnancy** | |
| --- | --- | --- | --- | --- | --- | --- | --- | --- | --- |
|  |  | **GroupPIP** | **CondPIP** | **GroupPIP** | **CondPIP** | **GroupPIP** | **CondPIP** | **GroupPIP** | **CondPIP** |
| Hospital delivery rate | 1 | 1 | 0.995 | 1 | 1 | 0.855 | 0.873 | 0.936 | 0.128 |
| Antenatal care rate | 1 | 1 | 0.005 | 1 | 0 | 0.855 | 0.098 | 0.936 | 0.859 |
| Prenatal booking rate | 1 | 1 | 0 | 1 | 0 | 0.855 | 0.029 | 0.936 | 0.013 |
| Local fiscal expenditure on healthcare | 2 | 0.528 | 1 | 0.412 | 1 | 1 | 1 | 0.530 | 1 |
| Urbanization rate | 3 | 0.978 | 0.648 | 1 | 0.998 | 0.993 | 0.733 | 0.985 | 0.255 |
| PCDI | 3 | 0.978 | 0.347 | 1 | 0.002 | 0.993 | 0.256 | 0.985 | 0.706 |
| Average years of schooling for females | 3 | 0.978 | 0.005 | 1 | 0 | 0.993 | 0.011 | 0.985 | 0.039 |
| Number of Ob/Gyn beds per 1000 livebirths | 4 | 0.248 | 0.497 | 0.549 | 0.173 | 0.889 | 0.367 | 0.935 | 0.990 |
| Number of MCH personnel per 1000 livebirths | 4 | 0.248 | 0.503 | 0.549 | 0.827 | 0.889 | 0.633 | 0.935 | 0.010 |

Note: GroupPIP, group posterior inclusion probabilities; CondPIP, conditional posterior inclusion probabilities; MCH, maternal and child health; Ob/Gyn, obstetrics and gynecology; PCDI, per capita disposable income.
