# Supplementary material for: Social and health system factors associated with maternal mortality in Eastern and Western China: Population health estimates using provincial-level data
Source: PLoS Med. 2025 Dec 4;22(12):e1004837. doi: 10.1371/journal.pmed.1004837 (PMC12677549; doi:10.1371/journal.pmed.1004837)
Supplement: S7 Table — Note: GroupPIP, group posterior inclusion probabilities; CondPIP, conditional posterior inclusion probabilities; MCH, maternal and child health; Ob/Gyn, obstetrics and gynecology; PCDI, per capita disposable income. (DOCX) [file pmed.1004837.s007.docx]

**Table S7 Group and conditional posterior inclusion probabilities for each factor in Western China, 2013-2020, using Bayesian Kernel Machine Regression hierarchical variable selection.**

| **Exposure** | **Exposure group** | **Total maternal mortality** | | **Maternal mortality due to hemorrhage** | | **Maternal mortality due to coexisting medical diseases** | | **Maternal mortality due to hypertensive disorders in pregnancy** | |
| --- | --- | --- | --- | --- | --- | --- | --- | --- | --- |
|  |  | **GroupPIP** | **CondPIP** | **GroupPIP** | **CondPIP** | **GroupPIP** | **CondPIP** | **GroupPIP** | **CondPIP** |
| Hospital delivery rate | 1 | 1 | 0.122 | 1 | 1 | 0.984 | 0.562 | 1 | 0 |
| Antenatal care rate | 1 | 1 | 0.878 | 1 | 0 | 0.984 | 0.389 | 1 | 0 |
| Prenatal booking rate | 1 | 1 | 0 | 1 | 0 | 0.984 | 0.049 | 1 | 1 |
| Local fiscal expenditure on healthcare | 2 | 0.450 | 1 | 0.126 | 1 | 0.525 | 1 | 0.379 | 1 |
| Urbanization rate | 3 | 0.988 | 0.847 | 1 | 0 | 0.684 | 0.139 | 0.565 | 0.229 |
| PCDI | 3 | 0.988 | 0.138 | 1 | 1 | 0.684 | 0.065 | 0.565 | 0.503 |
| Average years of schooling for females | 3 | 0.988 | 0.015 | 1 | 0 | 0.684 | 0.795 | 0.565 | 0.267 |
| Number of Ob/Gyn beds per 1000 livebirths | 4 | 0.336 | 0.100 | 0.15 | 0.279 | 0.413 | 0.534 | 0.678 | 0.426 |
| Number of MCH personnel per 1000 livebirths | 4 | 0.336 | 0.899 | 0.15 | 0.721 | 0.413 | 0.466 | 0.678 | 0.574 |

Note: GroupPIP, group posterior inclusion probabilities; CondPIP, conditional posterior inclusion probabilities; MCH, maternal and child health; Ob/Gyn, obstetrics and gynecology; PCDI, per capita disposable income.
