# Supplementary material for: Social and health system factors associated with maternal mortality in Eastern and Western China: Population health estimates using provincial-level data
Source: PLoS Med. 2025 Dec 4;22(12):e1004837. doi: 10.1371/journal.pmed.1004837 (PMC12677549; doi:10.1371/journal.pmed.1004837)
Supplement: S8 Table — Note: Effects are shown as estimates and the 95% confidence intervals in the linear mixed effects models. (DOCX) [file pmed.1004837.s008.docx]

**Table S8 Effects of factors on maternal mortality, using univariate linear mixed-effects models weighted by the number of livebirths.**

| **Outcome** | **Factor** | **2004-2012** | | **2013-2020** | |
| --- | --- | --- | --- | --- | --- |
|  |  | **East** | **West** | **East** | **West** |
| Total maternal mortality | Hospital delivery rate | -1.51 (-1.62, -1.41) | -2.37 (-2.66, -2.09) | -4.42 (-5.34, -3.5) | -6.95 (-8.06, -5.84) |
|  | Antenatal care rate | -2.37 (-2.77, -1.97) | -6.30 (-7.15, -5.45) | -1.16 (-1.52, -0.81) | -1.66 (-2.62, -0.70) |
|  | Local fiscal expenditure on healthcare | -0.07 (-0.08, -0.06) | -0.28 (-0.35, -0.21) | -0.01 (-0.01, 0) | -0.07 (-0.11, -0.03) |
|  | Urbanization rate | -0.95 (-1.06, -0.84) | -2.13 (-2.86, -1.39) | -0.33 (-0.40, -0.25) | -1.28 (-1.78, -0.79) |
|  | Per capita disposable income | -21.1 (-24.2, -18.0) | -59.8 (-73.8, -45.8) | -2.13 (-2.72, -1.54) | -10.4 (-15.2, -5.47) |
| Maternal mortality due to hemorrhage | Hospital delivery rate | -1.07 (-1.13, -1.01) | -1.31 (-1.48, -1.15) | -2.81 (-3.16, -2.46) | -3.76 (-4.23, -3.29) |
|  | Antenatal care rate | -1.47 (-1.76, -1.19) | -3.37 (-3.78, -2.96) | -0.46 (-0.63, -0.29) | -1.96 (-2.33, -1.59) |
|  | Local fiscal expenditure on healthcare | -0.04 (-0.05, -0.04) | -0.15 (-0.19, -0.11) | 0 (0, 0) | -0.04 (-0.06, -0.01) |
|  | Urbanization rate | -0.61 (-0.69, -0.54) | -1.28 (-1.67, -0.90) | -0.16 (-0.19, -0.12) | -0.73 (-1, -0.46) |
|  | Per capita disposable income | -13.0 (-15.2, -10.7) | -32.9 (-40.8, -25.0) | -0.99 (-1.26, -0.72) | -5.51 (-8.44, -2.58) |
| Maternal mortality due to coexisting medical diseases | Hospital delivery rate | -0.31 (-0.35, -0.27) | -0.62 (-0.72, -0.52) | -0.76 (-1.21, -0.32) | -0.69 (-1.09, -0.3) |
|  | Antenatal care rate | -0.52 (-0.63, -0.42) | -1.44 (-1.77, -1.11) | -0.23 (-0.37, -0.08) | -0.16 (-0.4, 0.07) |
|  | Local fiscal expenditure on healthcare | -0.02 (-0.02, -0.01) | -0.07 (-0.1, -0.05) | 0 (0, 0) | -0.01 (-0.02, 0) |
|  | Urbanization rate | -0.23 (-0.26, -0.2) | -0.53 (-0.75, -0.31) | -0.06 (-0.09, -0.03) | -0.16 (-0.29, -0.04) |
|  | Per capita disposable income | -5.16 (-5.92, -4.40) | -15.8 (-20.3, -11.4) | -0.54 (-0.77, -0.31) | -0.83 (-2.21, 0.55) |
| Maternal mortality due to hypertensive disorders in pregnancy | Hospital delivery rate | -0.16 (-0.17, -0.14) | -0.53 (-0.65, -0.41) | -0.39 (-0.59, -0.18) | -1.17 (-1.5, -0.84) |
|  | Antenatal care rate | -0.26 (-0.31, -0.21) | -1.47 (-1.74, -1.21) | -0.13 (-0.2, -0.06) | -0.41 (-0.63, -0.19) |
|  | Local fiscal expenditure on healthcare | -0.01 (-0.01, -0.01) | -0.05 (-0.08, -0.03) | 0 (0, 0) | -0.01 (-0.02, 0) |
|  | Urbanization rate | -0.1 (-0.12, -0.09) | -0.39 (-0.62, -0.17) | -0.03 (-0.04, -0.02) | -0.23 (-0.36, -0.11) |
|  | Per capita disposable income | -2.33 (-2.7, -1.95) | -11.7 (-16.5, -6.96) | -0.27 (-0.38, -0.16) | -1.77 (-3.11, -0.43) |

Note: Effects are shown as estimates and the 95% confidence intervals in the linear mixed effects models.
