# Supplementary material for: Social and health system factors associated with maternal mortality in Eastern and Western China: Population health estimates using provincial-level data
Source: PLoS Med. 2025 Dec 4;22(12):e1004837. doi: 10.1371/journal.pmed.1004837 (PMC12677549; doi:10.1371/journal.pmed.1004837)
Supplement: S9 Table — Note: Effects are shown as estimates and the 95% confidence intervals in the linear mixed effects model, adjusting for the other eight factors. For example, the effect of hospital delivery rate was estimated when urbanization rate, per capita disposable income, average years of schooling, number of health technical personnel in maternal and child health, number of hospital beds for obstetrics and gynecology, local fiscal expenditure on healthcare, prenatal booking rate, and antenatal care rate were adjusted for. (DOCX) [file pmed.1004837.s009.docx]

**Table S9 Effects of factors on maternal mortality, using multivariate linear mixed-effects models weighted by the number of livebirths.**

| **Outcome** | **Factor** | **2004-2012** | | **2013-2020** | |
| --- | --- | --- | --- | --- | --- |
|  |  | **East** | **West** | **East** | **West** |
| Total maternal mortality | Hospital delivery rate | -1.09 (-1.22, -0.95) | -1.94 (-2.56, -1.31) | -3.09 (-4.05, -2.13) | -4.93 (-6.57, -3.29) |
|  | Antenatal care rate | -0.05 (-0.46, 0.36) | -1.90 (-3.97, 0.17) | -0.64 (-1.00, -0.29) | -0.96 (-1.97, 0.05) |
|  | Local fiscal expenditure on healthcare | -0.01 (-0.02, 0.01) | 0.07 (-0.07, 0.22) | 0 (0, 0.01) | -0.02 (-0.06, 0.02) |
|  | Urbanization rate | -0.27 (-0.42, -0.13) | 0.98 (0.22, 1.74) | -0.16 (-0.31, -0.01) | -0.60 (-1.47, 0.27) |
|  | Per capita disposable income | -1.42 (-5.78, 2.94) | -30.5 (-55.9, -5.18) | 0.21 (-1.05, 1.46) | 6.88 (-2.27, 16.02) |
| Maternal mortality due to hemorrhage | Hospital delivery rate | -0.90 (-0.99, -0.82) | -0.86 (-1.22, -0.5) | -2.16 (-2.52, -1.08) | -2.78 (-3.54, -2.02) |
|  | Antenatal care rate | -0.02 (-0.27, 0.23) | -2.01 (-3.11, -0.92) | -0.17 (-0.3, -0.04) | -1.05 (-1.61, -0.49) |
|  | Local fiscal expenditure on healthcare | 0 (-0.01, 0) | 0.04 (-0.03, 0.1) | 0 (0, 0) | -0.01 (-0.02, 0) |
|  | Urbanization rate | -0.14 (-0.23, -0.06) | -0.01 (-0.44, 0.43) | -0.11 (-0.16, -0.06) | -0.36 (-0.64, -0.08) |
|  | Per capita disposable income | 1.11 (-1.58, 3.79) | -3.4 (-18.0, 11.1) | 0.56 (0.11, 1.01) | 3.57 (-0.35, 7.49) |
| Maternal mortality due to coexisting medical diseases | Hospital delivery rate | -0.16 (-0.21, -0.11) | -0.59 (-0.80, -0.37) | -0.39 (-0.87, 0.08) | -0.58 (-1.20, 0.04) |
|  | Antenatal care rate | 0.09 (-0.05, 0.22) | -0.37 (-1.04, 0.3) | -0.04 (-0.21, 0.12) | 0.01 (-0.4, 0.42) |
|  | Local fiscal expenditure on healthcare | 0 (-0.01, 0) | 0 (-0.05, 0.05) | 0 (0, 0) | -0.02 (-0.03, -0.01) |
|  | Urbanization rate | -0.10 (-0.15, -0.06) | 0.48 (0.22, 0.75) | -0.03 (-0.09, 0.03) | -0.27 (-0.54, 0) |
|  | Per capita disposable income | -0.45 (-1.95, 1.05) | -8.51 (-17.4, 0.41) | -0.13 (-0.66, 0.41) | 5.14 (1.77, 8.52) |
| Maternal mortality due to hypertensive disorders in pregnancy | Hospital delivery rate | -0.10 (-0.12, -0.08) | -0.38 (-0.63, -0.12) | -0.14 (-0.36, 0.07) | -1.13 (-1.71, -0.55) |
|  | Antenatal care rate | 0.02 (-0.04, 0.08) | -1.05 (-1.83, -0.26) | -0.08 (-0.16, -0.01) | -0.12 (-0.52, 0.27) |
|  | Local fiscal expenditure on healthcare | 0 (0, 0) | 0.05 (0, 0.1) | 0 (0, 0) | 0 (-0.01, 0.01) |
|  | Urbanization rate | -0.03 (-0.05, -0.01) | 0.26 (-0.05, 0.57) | -0.03 (-0.05, 0) | -0.06 (-0.31, 0.18) |
|  | Per capita disposable income | 0.01 (-0.66, 0.68) | -7.63 (-18.2, 2.91) | -0.04 (-0.29, 0.21) | 1.45 (-1.69, 4.59) |

Note: Effects are shown as estimates and the 95% confidence intervals in the linear mixed effects model adjusting for the other eight determinants. For example, the effect of hospital delivery rate was estimated when urbanization rate, per capita disposable income, average years of schooling, number of health technical personnel in maternal and child health, number of hospital beds for obstetrics and gynecology, local fiscal expenditure on healthcare, prenatal booking rate, and antenatal care rate were adjusted for.
