# Supplementary material for: Social and health system factors associated with maternal mortality in Eastern and Western China: Population health estimates using provincial-level data
Source: PLoS Med. 2025 Dec 4;22(12):e1004837. doi: 10.1371/journal.pmed.1004837 (PMC12677549; doi:10.1371/journal.pmed.1004837)
Supplement: S10 Table — Note: GroupPIP, group posterior inclusion probabilities; CondPIP, conditional posterior inclusion probabilities; MCH, maternal and child health; Ob/Gyn, obstetrics and gynecology; PCDI, per capita disposable income. (DOCX) [file pmed.1004837.s010.docx]

**Table S10 Group and conditional posterior inclusion probabilities for each factor in Eastern China, 2004-2012, using Bayesian Kernel Machine Regression hierarchical variable selection with fiscal expenditure adjusted for inflation.**

| **Exposure** | **Exposure group** | **Total maternal mortality** | | **Maternal mortality due to hemorrhage** | | **Maternal mortality due to coexisting medical diseases** | | **Maternal mortality due to hypertensive disorders in pregnancy** | |
| --- | --- | --- | --- | --- | --- | --- | --- | --- | --- |
|  |  | **GroupPIP** | **CondPIP** | **GroupPIP** | **CondPIP** | **GroupPIP** | **CondPIP** | **GroupPIP** | **CondPIP** |
| Hospital delivery rate | 1 | 1 | 1 | 1 | 1 | 1 | 0.767 | 1 | 0.860 |
| Antenatal care rate | 1 | 1 | 0 | 1 | 0 | 1 | 0.001 | 1 | 0.140 |
| Prenatal booking rate | 1 | 1 | 0 | 1 | 0 | 1 | 0.233 | 1 | 0 |
| Local fiscal expenditure on healthcare | 2 | 0.997 | 1 | 1 | 1 | 0.807 | 1 | 0.928 | 1 |
| Urbanization rate | 3 | 1 | 1 | 1 | 1 | 0.992 | 0.836 | 0.844 | 0.370 |
| PCDI | 3 | 1 | 0 | 1 | 0 | 0.992 | 0.164 | 0.844 | 0.617 |
| Average years of schooling for females | 3 | 1 | 0 | 1 | 0 | 0.992 | 0 | 0.844 | 0.013 |
| Number of Ob/Gyn beds per 1000 livebirths | 4 | 0.881 | 0.046 | 0.917 | 0.025 | 0.641 | 0.727 | 0.735 | 0.939 |
| Number of MCH personnel per 1000 livebirths | 4 | 0.881 | 0.953 | 0.917 | 0.974 | 0.641 | 0.272 | 0.735 | 0.061 |

Note: GroupPIP, group posterior inclusion probabilities; CondPIP, conditional posterior inclusion probabilities; MCH, maternal and child health; Ob/Gyn, obstetrics and gynecology; PCDI, per capita disposable income.
