# Supplementary material for: Social and health system factors associated with maternal mortality in Eastern and Western China: Population health estimates using provincial-level data
Source: PLoS Med. 2025 Dec 4;22(12):e1004837. doi: 10.1371/journal.pmed.1004837 (PMC12677549; doi:10.1371/journal.pmed.1004837)
Supplement: S11 Table — Note: GroupPIP, group posterior inclusion probabilities; CondPIP, conditional posterior inclusion probabilities; MCH, maternal and child health; Ob/Gyn, obstetrics and gynecology; PCDI, per capita disposable income. (DOCX) [file pmed.1004837.s011.docx]

**Table S11 Group and conditional posterior inclusion probabilities for each factor in Western China, 2004-2012, using Bayesian Kernel Machine Regression hierarchical variable selection with fiscal expenditure adjusted for inflation.**

| **Exposure** | **Exposure group** | **Total maternal mortality** | | **Maternal mortality due to hemorrhage** | | **Maternal mortality due to coexisting medical diseases** | | **Maternal mortality due to hypertensive disorders in pregnancy** | |
| --- | --- | --- | --- | --- | --- | --- | --- | --- | --- |
|  |  | **GroupPIP** | **CondPIP** | **GroupPIP** | **CondPIP** | **GroupPIP** | **CondPIP** | **GroupPIP** | **CondPIP** |
| Hospital delivery rate | 1 | 1 | 0.012 | 1 | 0.186 | 1 | 0.994 | 1 | 0 |
| Antenatal care rate | 1 | 1 | 0.949 | 1 | 0.722 | 1 | 0.005 | 1 | 1 |
| Prenatal booking rate | 1 | 1 | 0.040 | 1 | 0.098 | 1 | 0.001 | 1 | 0 |
| Local fiscal expenditure on healthcare | 2 | 0.37 | 1 | 0.302 | 1 | 0.406 | 1 | 0.020 | 1 |
| Urbanization rate | 3 | 1 | 0 | 0.957 | 0.006 | 0.533 | 0.243 | 0.121 | 0.061 |
| PCDI | 3 | 1 | 1 | 0.957 | 0.944 | 0.533 | 0.104 | 0.121 | 0.876 |
| Average years of schooling for females | 3 | 1 | 0 | 0.957 | 0.050 | 0.533 | 0.652 | 0.121 | 0.063 |
| Number of Ob/Gyn beds per 1000 livebirths | 4 | 0.637 | 0.364 | 0.406 | 0.407 | 0.335 | 0.551 | 0.040 | 0.218 |
| Number of MCH personnel per 1000 livebirths | 4 | 0.637 | 0.636 | 0.406 | 0.592 | 0.335 | 0.449 | 0.040 | 0.782 |

Note: GroupPIP, group posterior inclusion probabilities; CondPIP, conditional posterior inclusion probabilities; MCH, maternal and child health; Ob/Gyn, obstetrics and gynecology; PCDI, per capita disposable income.
