# Supplementary material for: Social and health system factors associated with maternal mortality in Eastern and Western China: Population health estimates using provincial-level data
Source: PLoS Med. 2025 Dec 4;22(12):e1004837. doi: 10.1371/journal.pmed.1004837 (PMC12677549; doi:10.1371/journal.pmed.1004837)
Supplement: S12 Table — Note: GroupPIP, group posterior inclusion probabilities; CondPIP, conditional posterior inclusion probabilities; MCH, maternal and child health; Ob/Gyn, obstetrics and gynecology; PCDI, per capita disposable income. (DOCX) [file pmed.1004837.s012.docx]

**Table S12 Group and conditional posterior inclusion probabilities for each factor in Eastern China, 2013-2020, using Bayesian Kernel Machine Regression hierarchical variable selection with fiscal expenditure adjusted for inflation.**

| **Exposure** | **Exposure group** | **Total maternal mortality** | | **Maternal mortality due to hemorrhage** | | **Maternal mortality due to coexisting medical diseases** | | **Maternal mortality due to hypertensive disorders in pregnancy** | |
| --- | --- | --- | --- | --- | --- | --- | --- | --- | --- |
|  |  | **GroupPIP** | **CondPIP** | **GroupPIP** | **CondPIP** | **GroupPIP** | **CondPIP** | **GroupPIP** | **CondPIP** |
| Hospital delivery rate | 1 | 1 | 1 | 1 | 1 | 0.710 | 0.772 | 0.909 | 0.085 |
| Antenatal care rate | 1 | 1 | 0 | 1 | 0 | 0.710 | 0.183 | 0.909 | 0.897 |
| Prenatal booking rate | 1 | 1 | 0 | 1 | 0 | 0.710 | 0.045 | 0.909 | 0.017 |
| Local fiscal expenditure on healthcare | 2 | 0.663 | 1 | 0.488 | 1 | 0.999 | 1 | 0.644 | 1 |
| Urbanization rate | 3 | 0.996 | 0.781 | 1 | 0.996 | 0.990 | 0.684 | 0.992 | 0.325 |
| PCDI | 3 | 0.996 | 0.205 | 1 | 0.004 | 0.990 | 0.300 | 0.992 | 0.652 |
| Average years of schooling for females | 3 | 0.996 | 0.014 | 1 | 0 | 0.990 | 0.016 | 0.992 | 0.022 |
| Number of Ob/Gyn beds per 1000 livebirths | 4 | 0.330 | 0.548 | 0.571 | 0.136 | 0.711 | 0.399 | 0.972 | 0.995 |
| Number of MCH personnel per 1000 livebirths | 4 | 0.330 | 0.452 | 0.571 | 0.864 | 0.711 | 0.601 | 0.972 | 0.005 |

Note: GroupPIP, group posterior inclusion probabilities; CondPIP, conditional posterior inclusion probabilities; MCH, maternal and child health; Ob/Gyn, obstetrics and gynecology; PCDI, per capita disposable income.
