# Supplementary material for: Social and health system factors associated with maternal mortality in Eastern and Western China: Population health estimates using provincial-level data
Source: PLoS Med. 2025 Dec 4;22(12):e1004837. doi: 10.1371/journal.pmed.1004837 (PMC12677549; doi:10.1371/journal.pmed.1004837)
Supplement: S13 Table — Note: GroupPIP, group posterior inclusion probabilities; CondPIP, conditional posterior inclusion probabilities; MCH, maternal and child health; Ob/Gyn, obstetrics and gynecology; PCDI, per capita disposable income. (DOCX) [file pmed.1004837.s013.docx]

**Table S13 Group and conditional posterior inclusion probabilities for each factor in Western China, 2013-2020, using Bayesian Kernel Machine Regression hierarchical variable selection with fiscal expenditure adjusted for inflation.**

| **Exposure** | **Exposure group** | **Total maternal mortality** | | **Maternal mortality due to hemorrhage** | | **Maternal mortality due to coexisting medical diseases** | | **Maternal mortality due to hypertensive disorders in pregnancy** | |
| --- | --- | --- | --- | --- | --- | --- | --- | --- | --- |
|  |  | **GroupPIP** | **CondPIP** | **GroupPIP** | **CondPIP** | **GroupPIP** | **CondPIP** | **GroupPIP** | **CondPIP** |
| Hospital delivery rate | 1 | 1 | 0 | 1 | 0.977 | 0.962 | 0.026 | 1 | 0 |
| Antenatal care rate | 1 | 1 | 1 | 1 | 0.023 | 0.962 | 0.902 | 1 | 0 |
| Prenatal booking rate | 1 | 1 | 0 | 1 | 0 | 0.962 | 0.072 | 1 | 1 |
| Local fiscal expenditure on healthcare | 2 | 0.773 | 1 | 0.268 | 1 | 0.673 | 1 | 0.360 | 1 |
| Urbanization rate | 3 | 1 | 0 | 1 | 0 | 0.899 | 0.088 | 0.480 | 0.229 |
| PCDI | 3 | 1 | 0 | 1 | 0.449 | 0.899 | 0.020 | 0.480 | 0.491 |
| Average years of schooling for females | 3 | 1 | 1 | 1 | 0.551 | 0.899 | 0.891 | 0.480 | 0.232 |
| Number of Ob/Gyn beds per 1000 livebirths | 4 | 0.832 | 0.040 | 0.487 | 0.700 | 0.473 | 0.638 | 0.705 | 0.662 |
| Number of MCH personnel per 1000 livebirths | 4 | 0.832 | 0.960 | 0.487 | 0.300 | 0.473 | 0.362 | 0.705 | 0.338 |

Note: GroupPIP, group posterior inclusion probabilities; CondPIP, conditional posterior inclusion probabilities; MCH, maternal and child health; Ob/Gyn, obstetrics and gynecology; PCDI, per capita disposable income.
