# Supplementary material for: Social and health system factors associated with maternal mortality in Eastern and Western China: Population health estimates using provincial-level data
Source: PLoS Med. 2025 Dec 4;22(12):e1004837. doi: 10.1371/journal.pmed.1004837 (PMC12677549; doi:10.1371/journal.pmed.1004837)
Supplement: S15 Table — Note: GroupPIP, group posterior inclusion probabilities; CondPIP, conditional posterior inclusion probabilities; MCH, maternal and child health; Ob/Gyn, obstetrics and gynecology; PCDI, per capita disposable income. (DOCX) [file pmed.1004837.s015.docx]

**Table S15 Group and conditional posterior inclusion probabilities for each factor in Western China, 2004-2012, using Bayesian Kernel Machine Regression hierarchical variable selection with missing data imputed by MICE.**

| **Exposure** | **Exposure group** | **Total maternal mortality** | | **Maternal mortality due to hemorrhage** | | **Maternal mortality due to coexisting medical diseases** | | **Maternal mortality due to hypertensive disorders in pregnancy** | |
| --- | --- | --- | --- | --- | --- | --- | --- | --- | --- |
|  |  | **GroupPIP** | **CondPIP** | **GroupPIP** | **CondPIP** | **GroupPIP** | **CondPIP** | **GroupPIP** | **CondPIP** |
| Hospital delivery rate | 1 | 1 | 0.178 | 1 | 0.307 | 0.999 | 0.987 | 1 | 0 |
| Antenatal care rate | 1 | 1 | 0.811 | 1 | 0.572 | 0.999 | 0.009 | 1 | 0 |
| Prenatal booking rate | 1 | 1 | 0.011 | 1 | 0.121 | 0.999 | 0.004 | 1 | 1 |
| Local fiscal expenditure on healthcare | 2 | 0.401 | 1 | 0.235 | 1 | 0.388 | 1 | 0.244 | 1 |
| Urbanization rate | 3 | 0.969 | 0.009 | 0.870 | 0.016 | 0.556 | 0.240 | 0.934 | 0.536 |
| PCDI | 3 | 0.969 | 0.884 | 0.870 | 0.875 | 0.556 | 0.142 | 0.934 | 0.294 |
| Average years of schooling for females | 3 | 0.969 | 0.106 | 0.870 | 0.108 | 0.556 | 0.618 | 0.934 | 0.170 |
| Number of Ob/Gyn beds per 1000 livebirths | 4 | 0.703 | 0.543 | 0.312 | 0.423 | 0.342 | 0.495 | 0.585 | 0.563 |
| Number of MCH personnel per 1000 livebirths | 4 | 0.703 | 0.457 | 0.312 | 0.577 | 0.342 | 0.505 | 0.585 | 0.437 |

Note: GroupPIP, group posterior inclusion probabilities; CondPIP, conditional posterior inclusion probabilities; MCH, maternal and child health; Ob/Gyn, obstetrics and gynecology; PCDI, per capita disposable income.
