# Supplementary figures and images for: Social and health system factors associated with maternal mortality in Eastern and Western China: Population health estimates using provincial-level data
Source: PLoS Med. 2025 Dec 4;22(12):e1004837. doi: 10.1371/journal.pmed.1004837 (PMC12677549; doi:10.1371/journal.pmed.1004837)

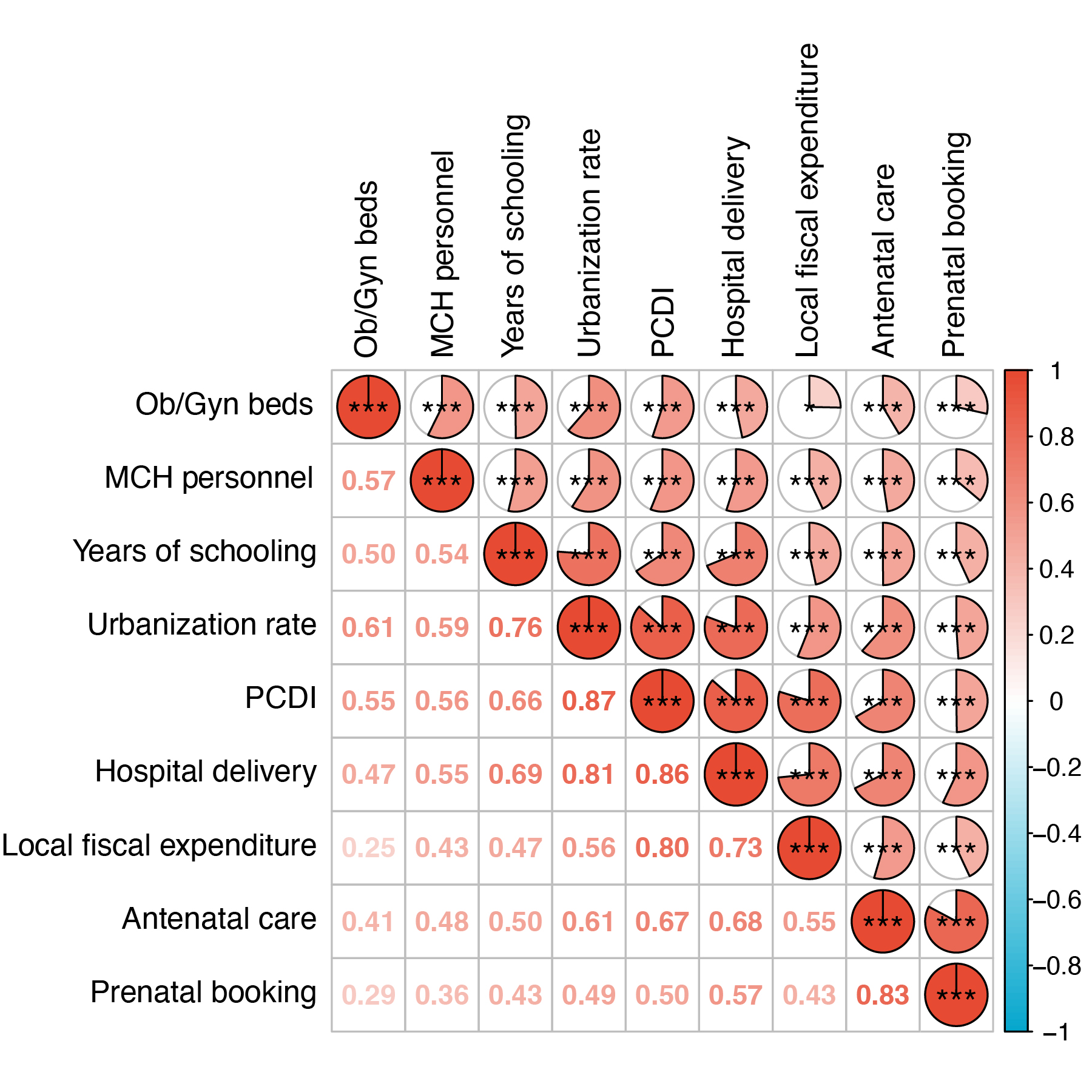

Supplement: S1 Fig — Note: The number in the cells represent the Spearman correlation coefficient. The circles are filled clockwise as pie charts to indicate the positive values of the correlation coefficients, and the color intensity represents the strength or the correlation. The asterisks in the circles indicate the significant levels with * representing a P-value of < 0.05, ** representing a P-value of < 0.01, and *** representing a P-value of < 0.001. Ob/Gyn beds: Number of hospital beds for obstetrics and gynecology per 1,000 livebirths; MCH personnel: Number of health technical personnel in maternal and child healthcare per 1,000 livebirths; Years of schooling: Average years of schooling for females; PCDI: per capita disposable income; Hospital delivery: Hospital delivery rate; Local fiscal expenditure: Local fiscal expenditure on healthcare; Antenatal care: Antenatal care rate; Prenatal booking: Prenatal booking rate (TIFF) [file pmed.1004837.s017.tiff]

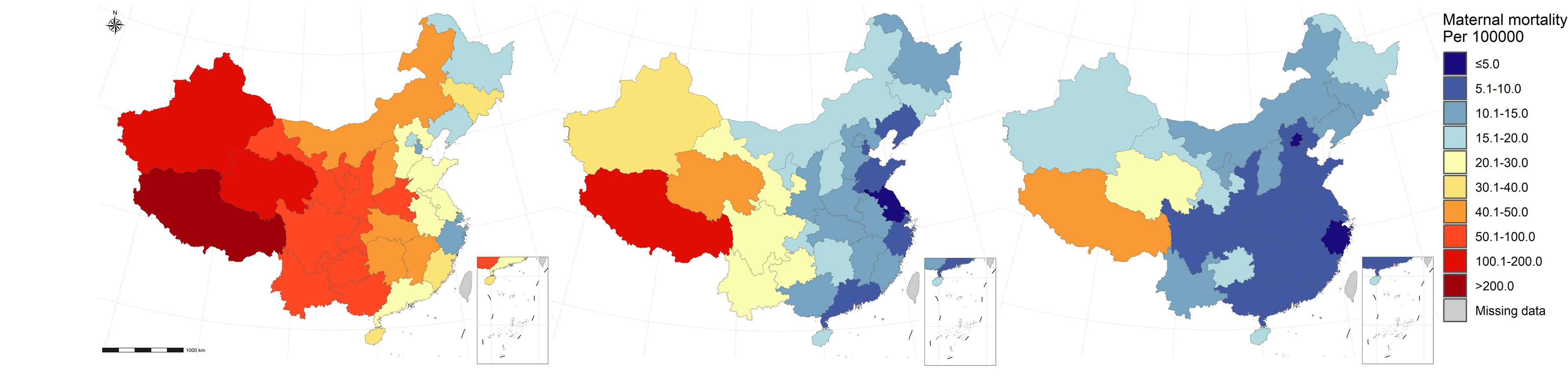

Supplement: S2 Fig — Note: The source of the basemap is: https://cloudcenter.tianditu.gov.cn/administrativeDivision. (TIFF) [file pmed.1004837.s018.tiff]

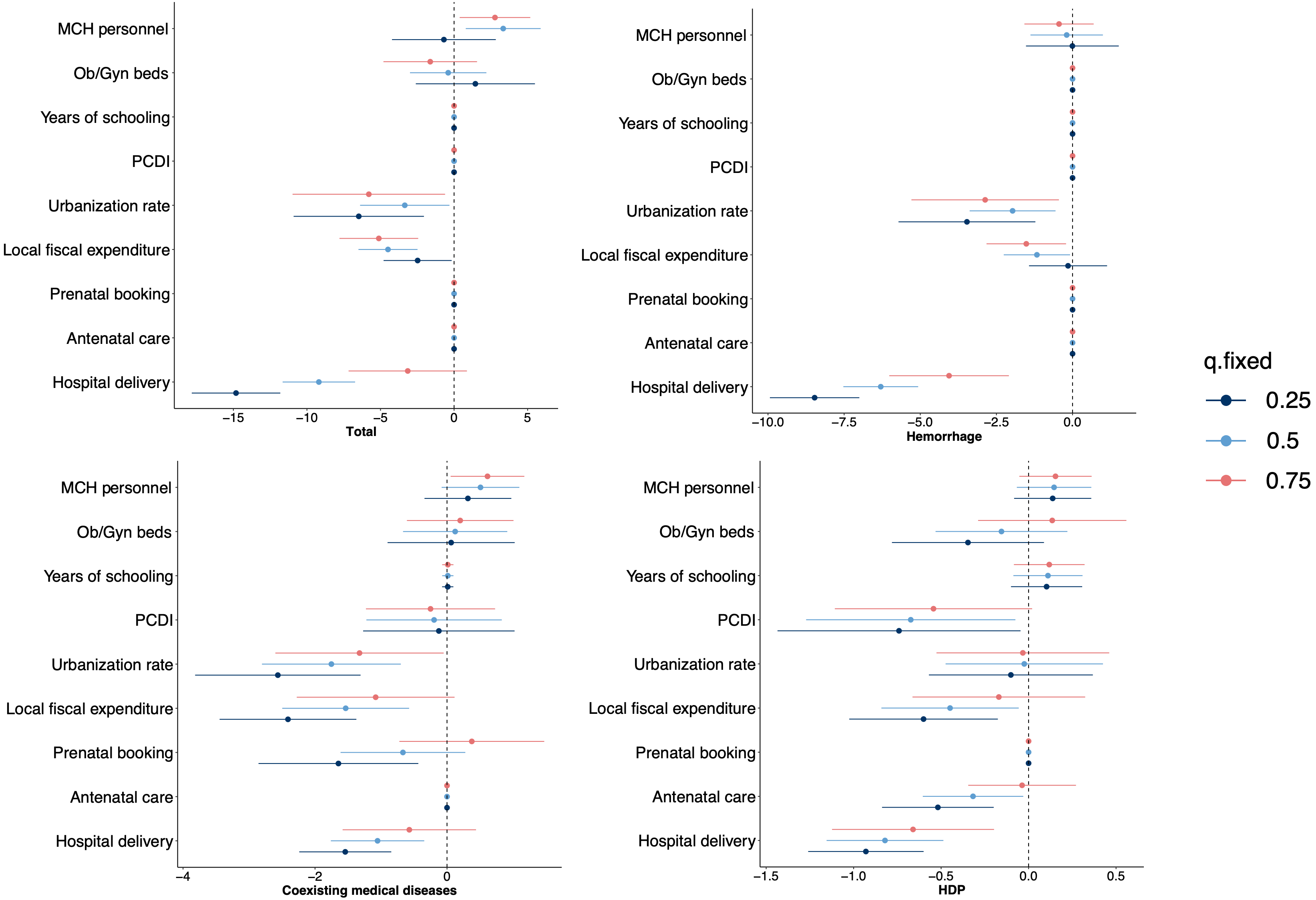

Supplement: S3 Fig — Note: The plot compares the exposure–response relationships associated with a change in a single exposure from the 75th percentile to the 25th percentile, when the other exposures are fixed at their 25th, 50th, and 75th percentiles. MCH, maternal and child health; Ob/Gyn, obstetrics and gynecology; PCDI, per capita disposable income; HDP, hypertensive disorders in pregnancy; q.fixed, quantiles at which to fix the remaining exposures. (TIFF) [file pmed.1004837.s019.tiff]

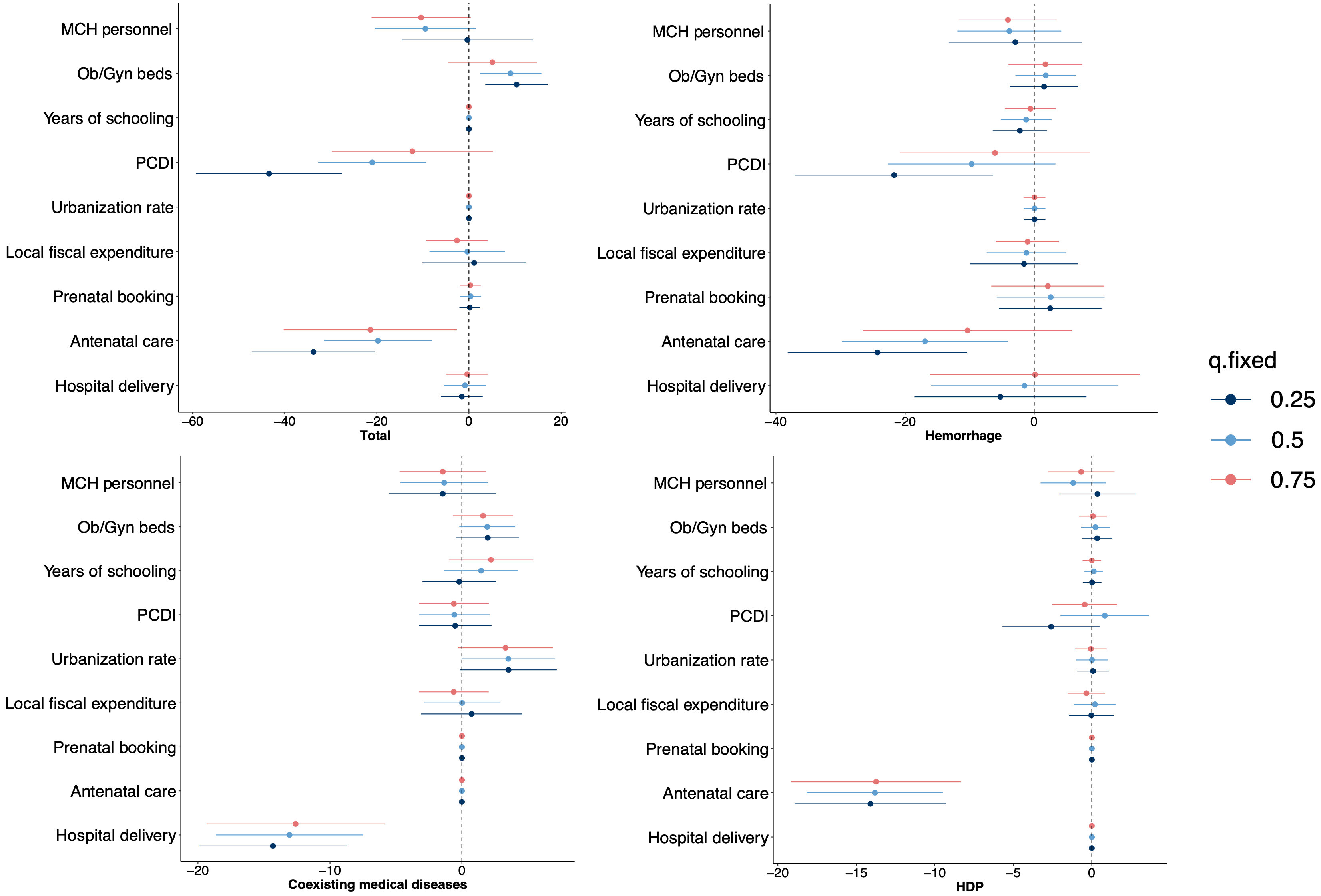

Supplement: S4 Fig — Note: The plot compares the exposure–response relationships associated with a change in a single exposure from the 75th percentile to the 25th percentile, when the other exposures are fixed at their 25th, 50th, and 75th percentiles. MCH, maternal and child health; Ob/Gyn, obstetrics and gynecology; PCDI, per capita disposable income; HDP, hypertensive disorders in pregnancy; q.fixed, quantiles at which to fix the remaining exposures. (TIFF) [file pmed.1004837.s020.tiff]

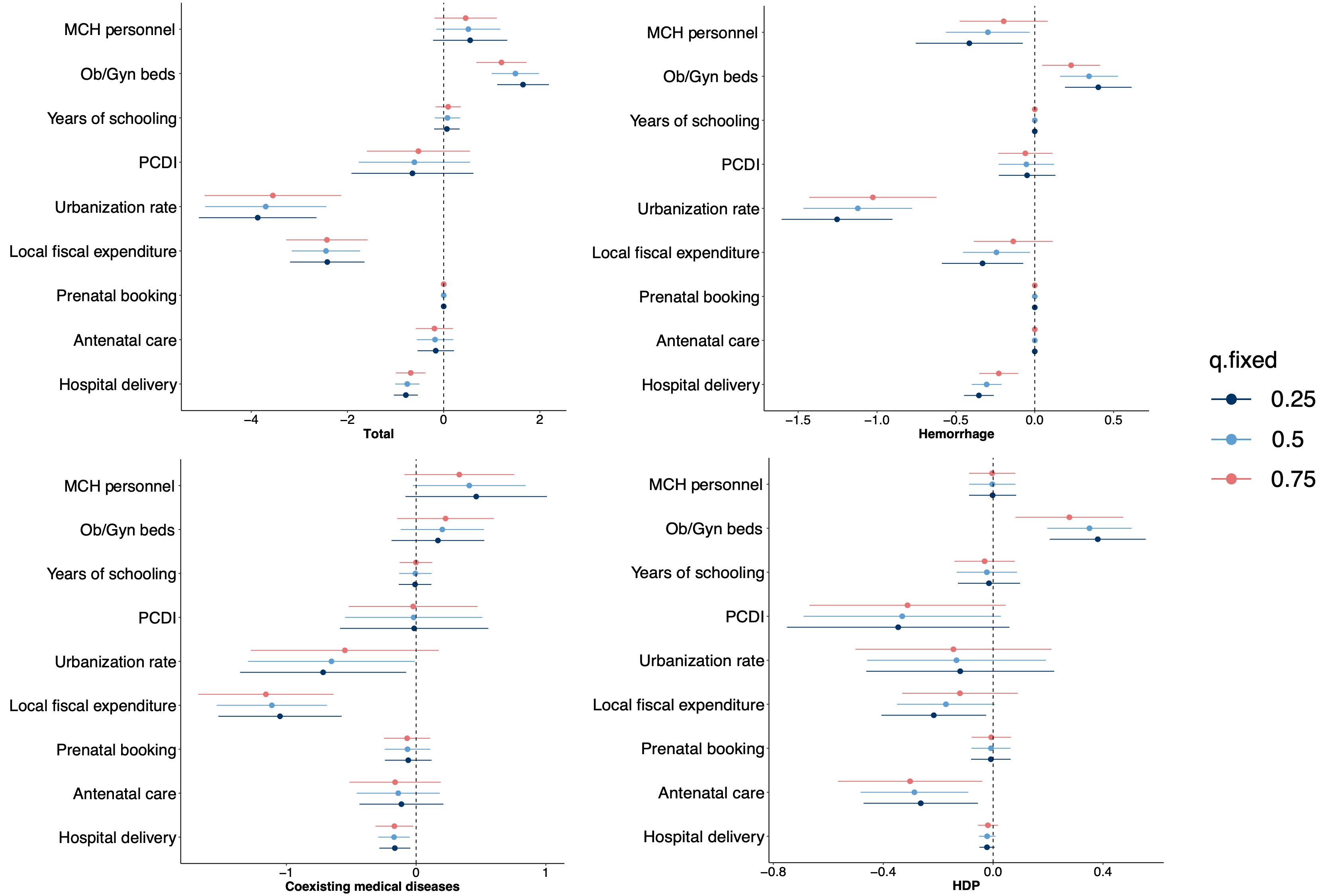

Supplement: S5 Fig — Note: The plot compares the exposure–response relationships associated with a change in a single exposure from the 75th percentile to the 25th percentile, when the other exposures are fixed at their 25th, 50th, and 75th percentiles. MCH, maternal and child health; Ob/Gyn, obstetrics and gynecology; PCDI, per capita disposable income; HDP, hypertensive disorders in pregnancy; q.fixed, quantiles at which to fix the remaining exposures. (TIFF) [file pmed.1004837.s021.tiff]

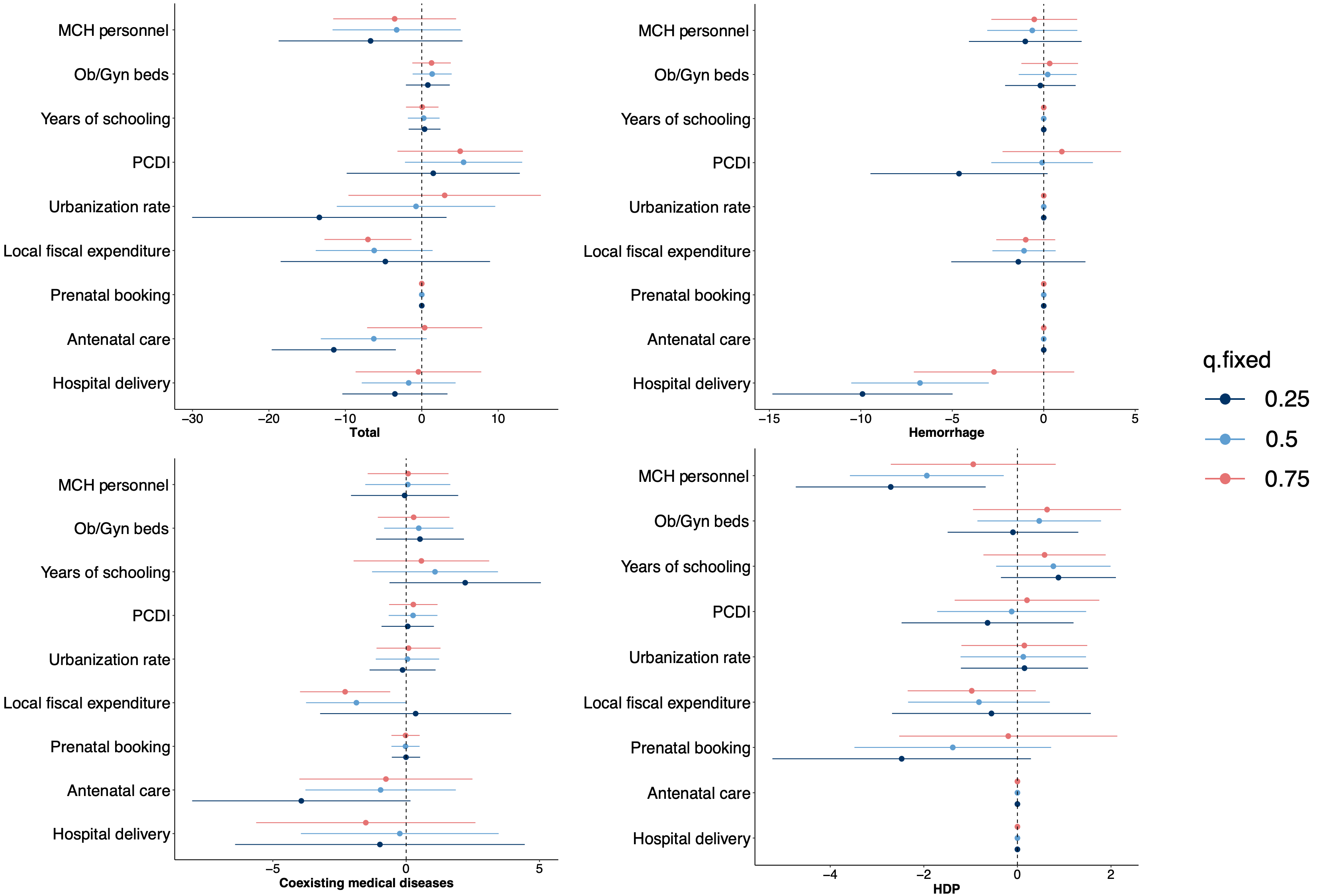

Supplement: S6 Fig — Note: The plot compares the exposure–response relationships associated with a change in a single exposure from the 75th percentile to the 25th percentile, when the other exposures are fixed at their 25th, 50th, and 75th percentiles. MCH, maternal and child health; Ob/Gyn, obstetrics and gynecology; PCDI, per capita disposable income; HDP, hypertensive disorders in pregnancy; q.fixed, quantiles at which to fix the remaining exposures. (TIFF) [file pmed.1004837.s022.tiff]

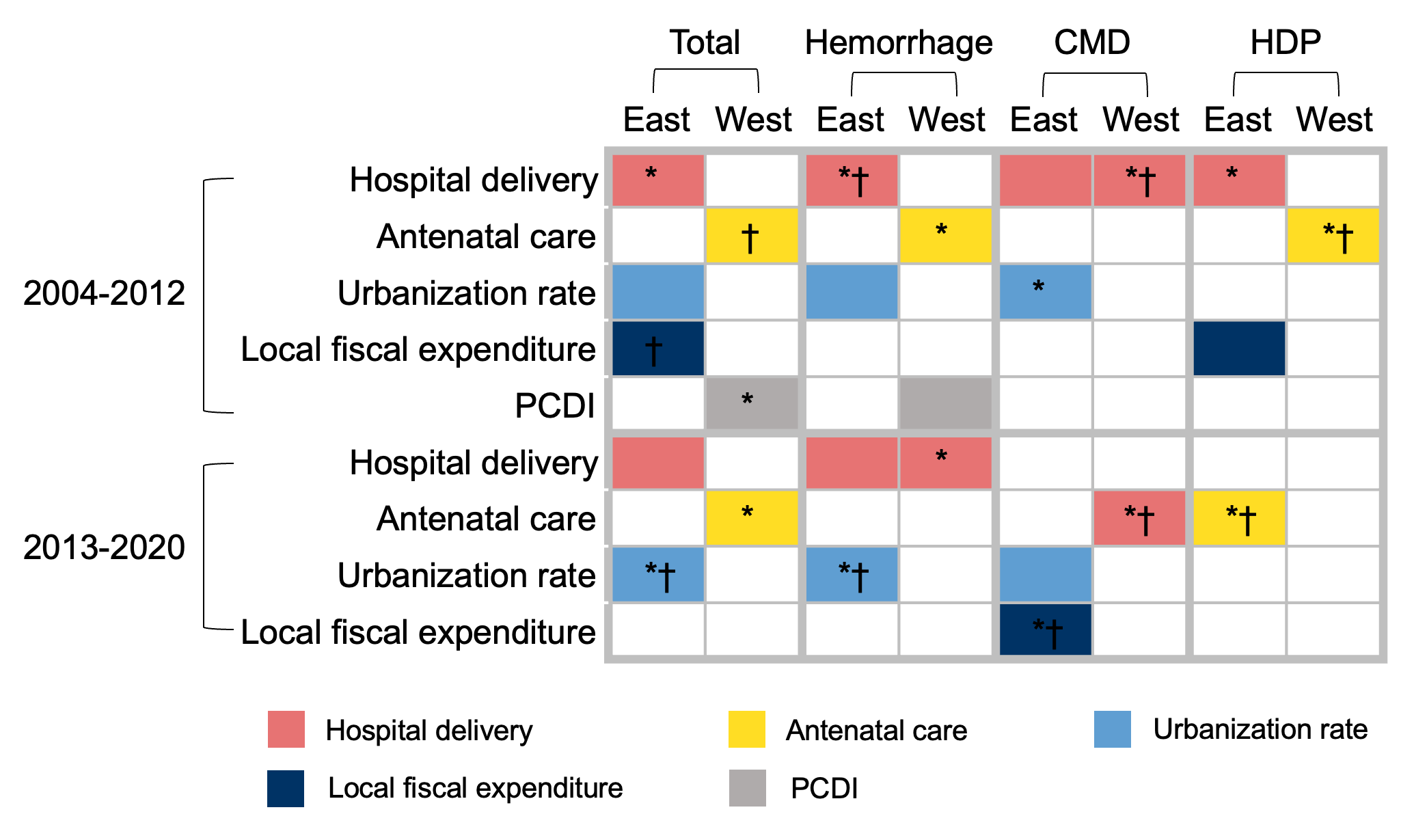

Supplement: S7 Fig — Note: The asterisk * and dagger † symbols in the cell represent that the factor contributes the most to the exposure–response relationship when all other factors are fixed at their 25th and 75th percentiles, respectively. White cell means that the factor is not identified as the component associated with reduced maternal mortality in the mixture. CMD, coexisting medical diseases; HDP, hypertensive disorders in pregnancy; PCDI, per capita disposable income. (TIFF) [file pmed.1004837.s023.tiff]

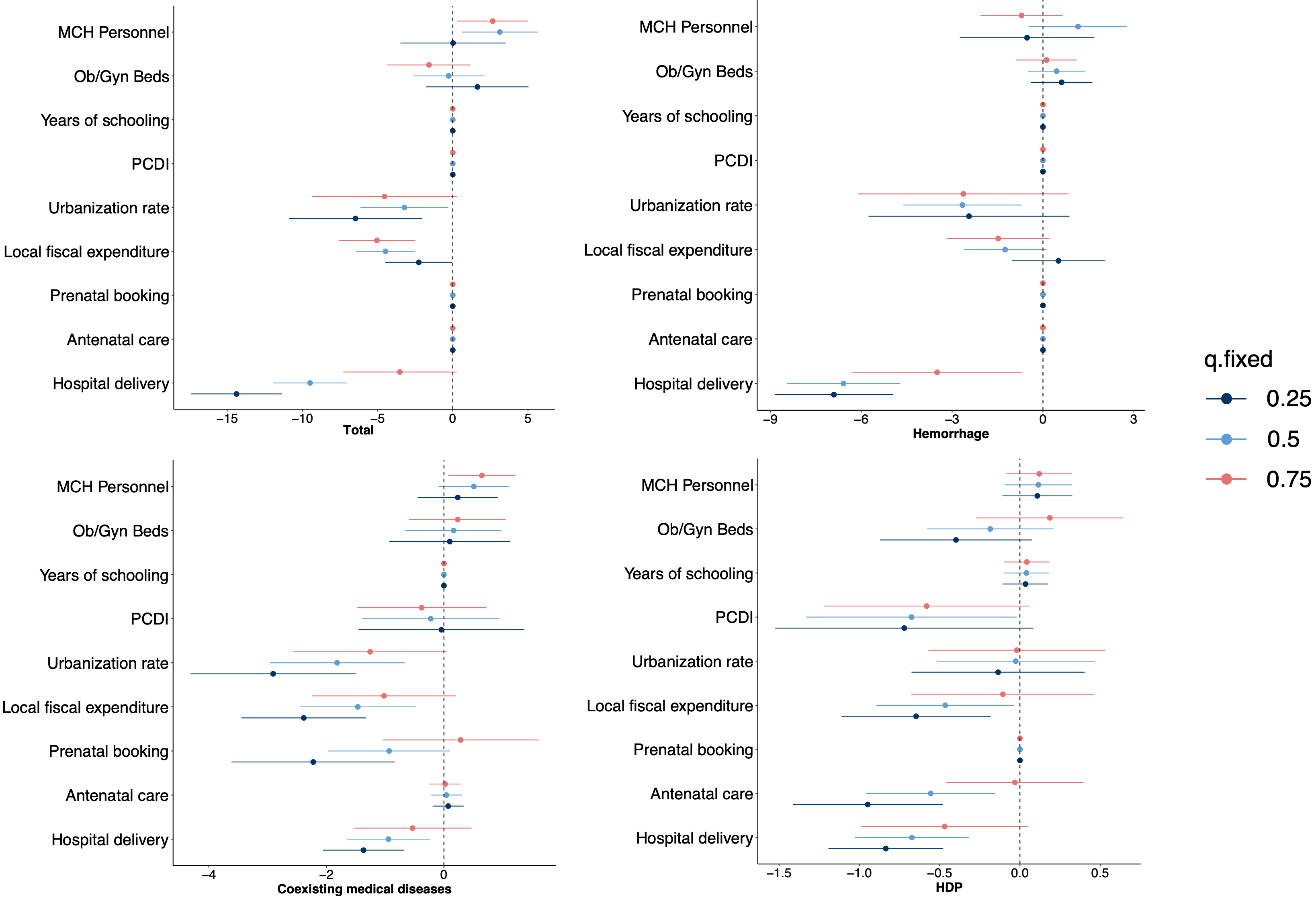

Supplement: S8 Fig — Note: The plot compares the exposure–response relationships associated with a change in a single exposure from the 75th percentile to the 25th percentile, when the other exposures are fixed at their 25th, 50th, and 75th percentiles. MCH, maternal and child health; Ob/Gyn, obstetrics and gynecology; PCDI, per capita disposable income; HDP, hypertensive disorders in pregnancy; q.fixed, quantiles at which to fix the remaining exposures. (TIFF) [file pmed.1004837.s024.tiff]

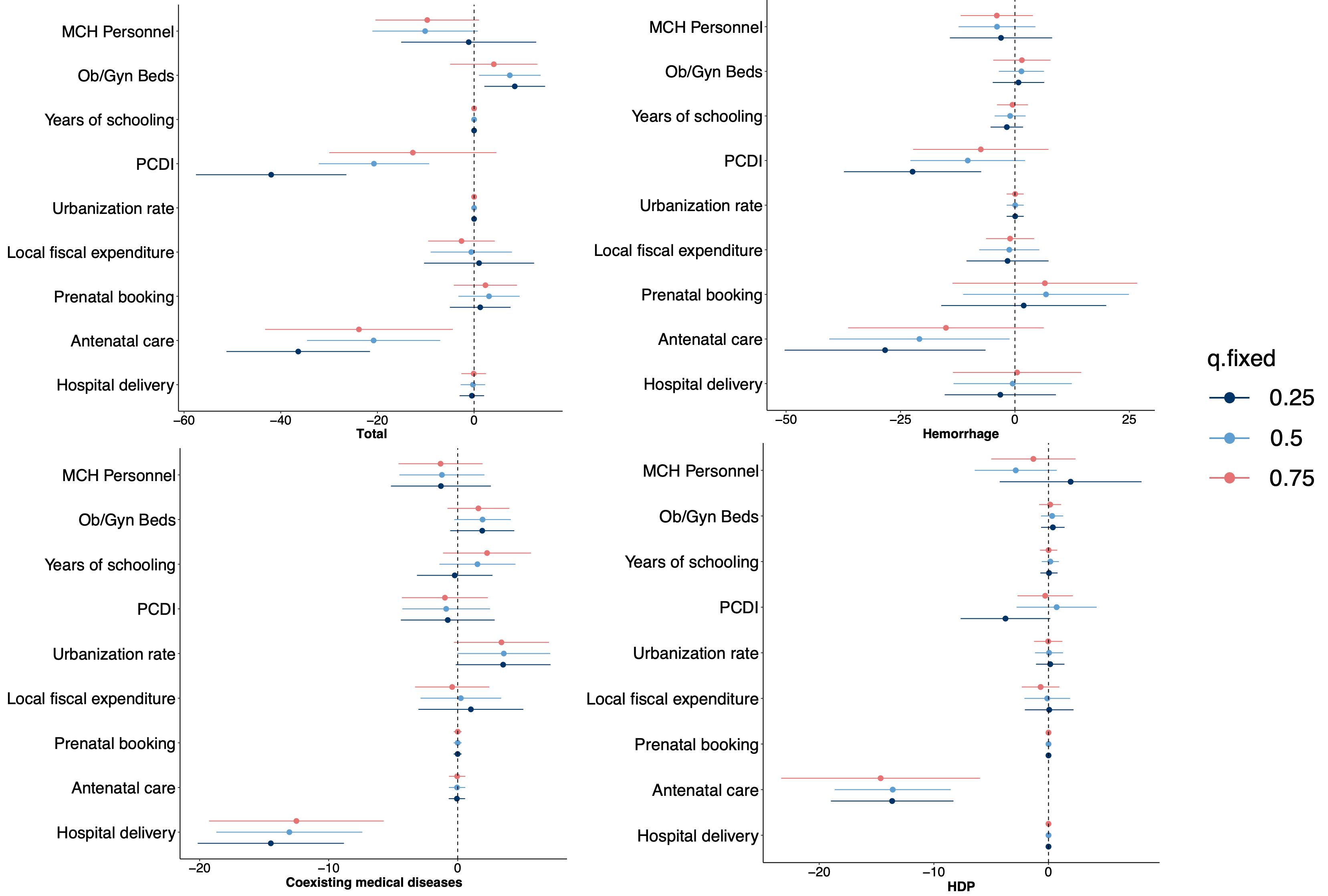

Supplement: S9 Fig — Note: The plot compares the exposure–response relationships associated with a change in a single exposure from the 75th percentile to the 25th percentile, when the other exposures are fixed at their 25th, 50th, and 75th percentiles. MCH, maternal and child health; Ob/Gyn, obstetrics and gynecology; PCDI, per capita disposable income; HDP, hypertensive disorders in pregnancy; q.fixed, quantiles at which to fix the remaining exposures. (TIFF) [file pmed.1004837.s025.tiff]

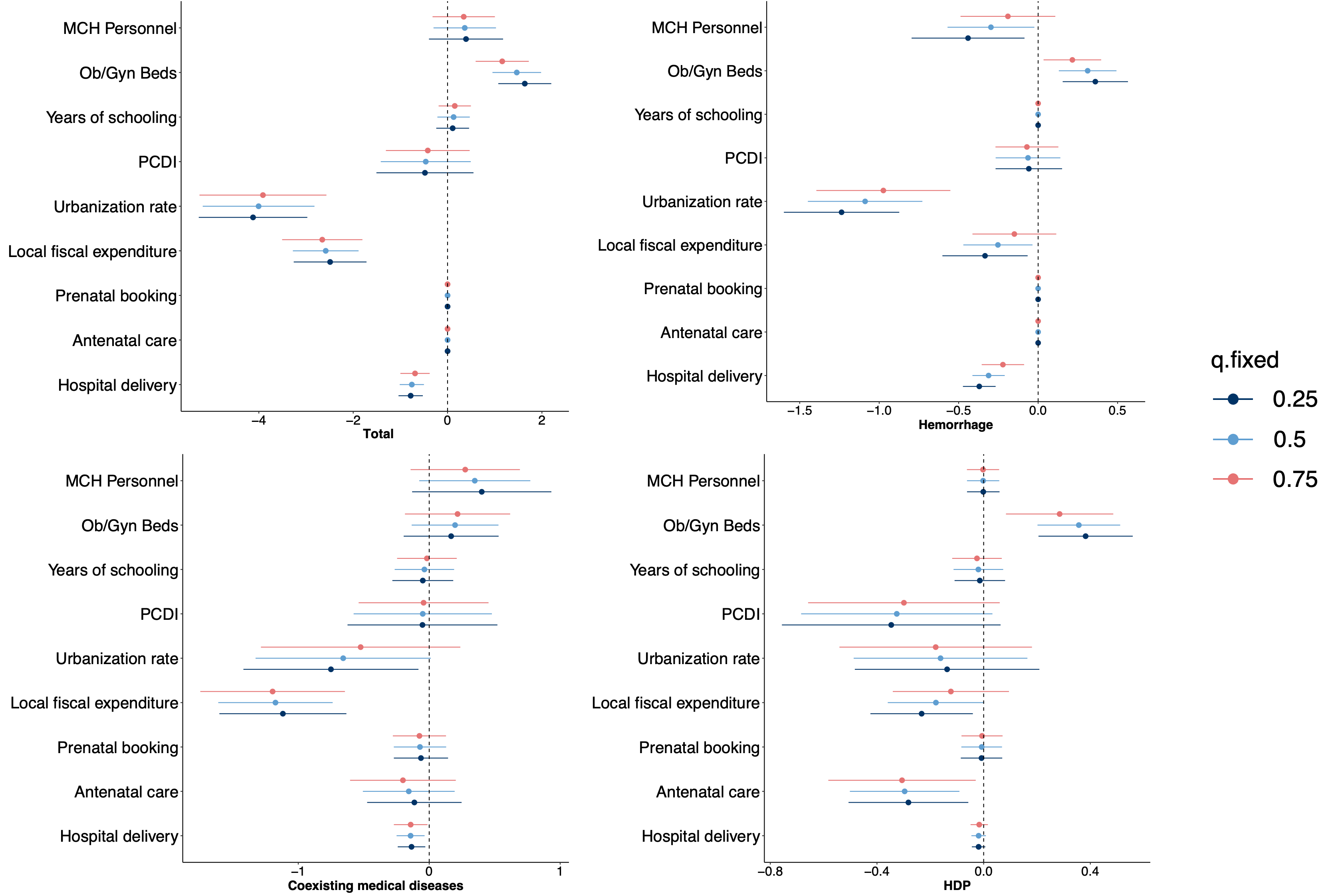

Supplement: S10 Fig — Note: The plot compares the exposure–response relationships associated with a change in a single exposure from the 75th percentile to the 25th percentile, when the other exposures are fixed at their 25th, 50th, and 75th percentiles. MCH, maternal and child health; Ob/Gyn, obstetrics and gynecology; PCDI, per capita disposable income; HDP, hypertensive disorders in pregnancy; q.fixed, quantiles at which to fix the remaining exposures (TIFF) [file pmed.1004837.s026.tiff]

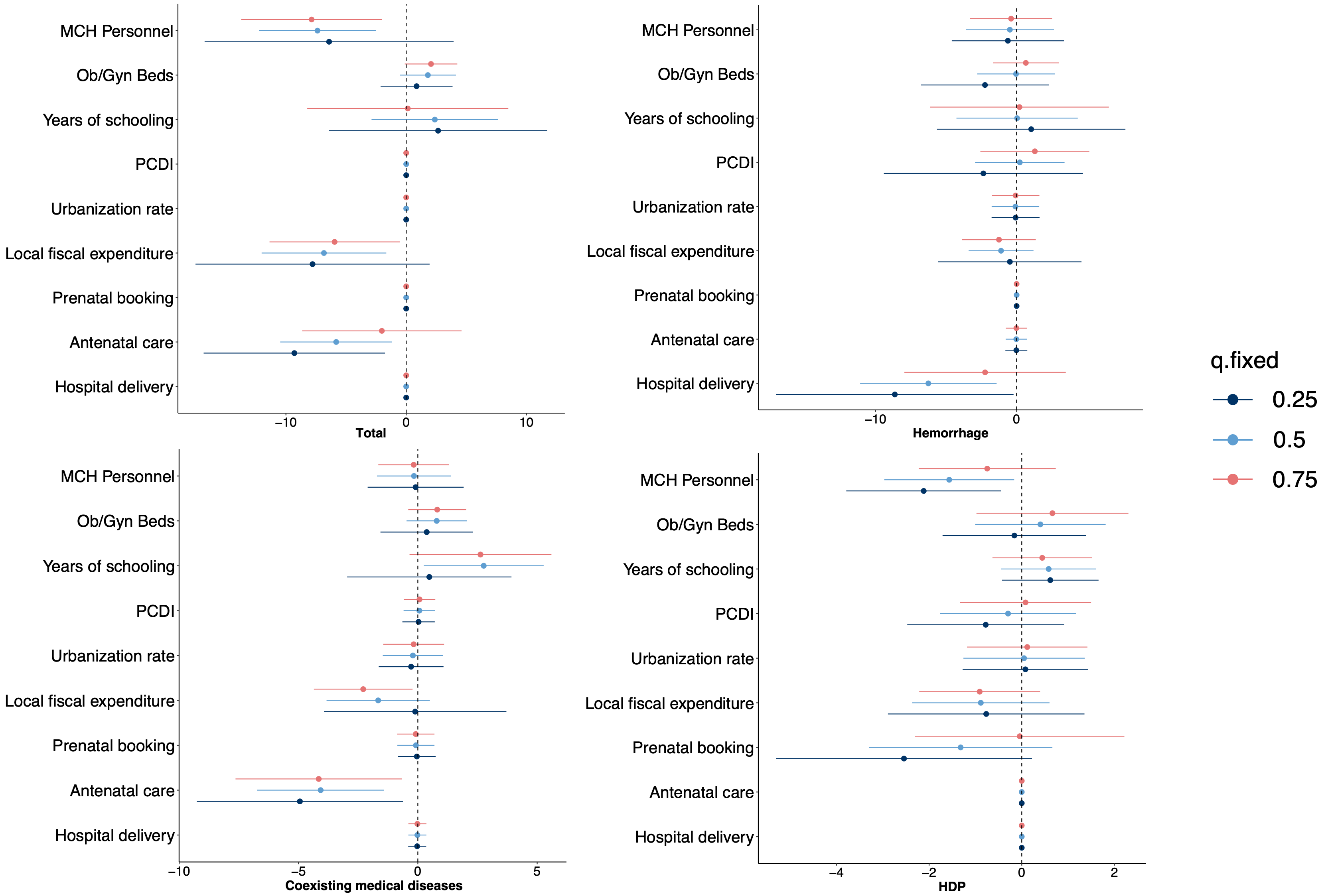

Supplement: S11 Fig — Note: The plot compares the exposure–response relationships associated with a change in a single exposure from the 75th percentile to the 25th percentile, when the other exposures are fixed at their 25th, 50th, and 75th percentiles. MCH, maternal and child health; Ob/Gyn, obstetrics and gynecology; PCDI, per capita disposable income; HDP, hypertensive disorders in pregnancy; q.fixed, quantiles at which to fix the remaining exposures. (TIFF) [file pmed.1004837.s027.tiff]

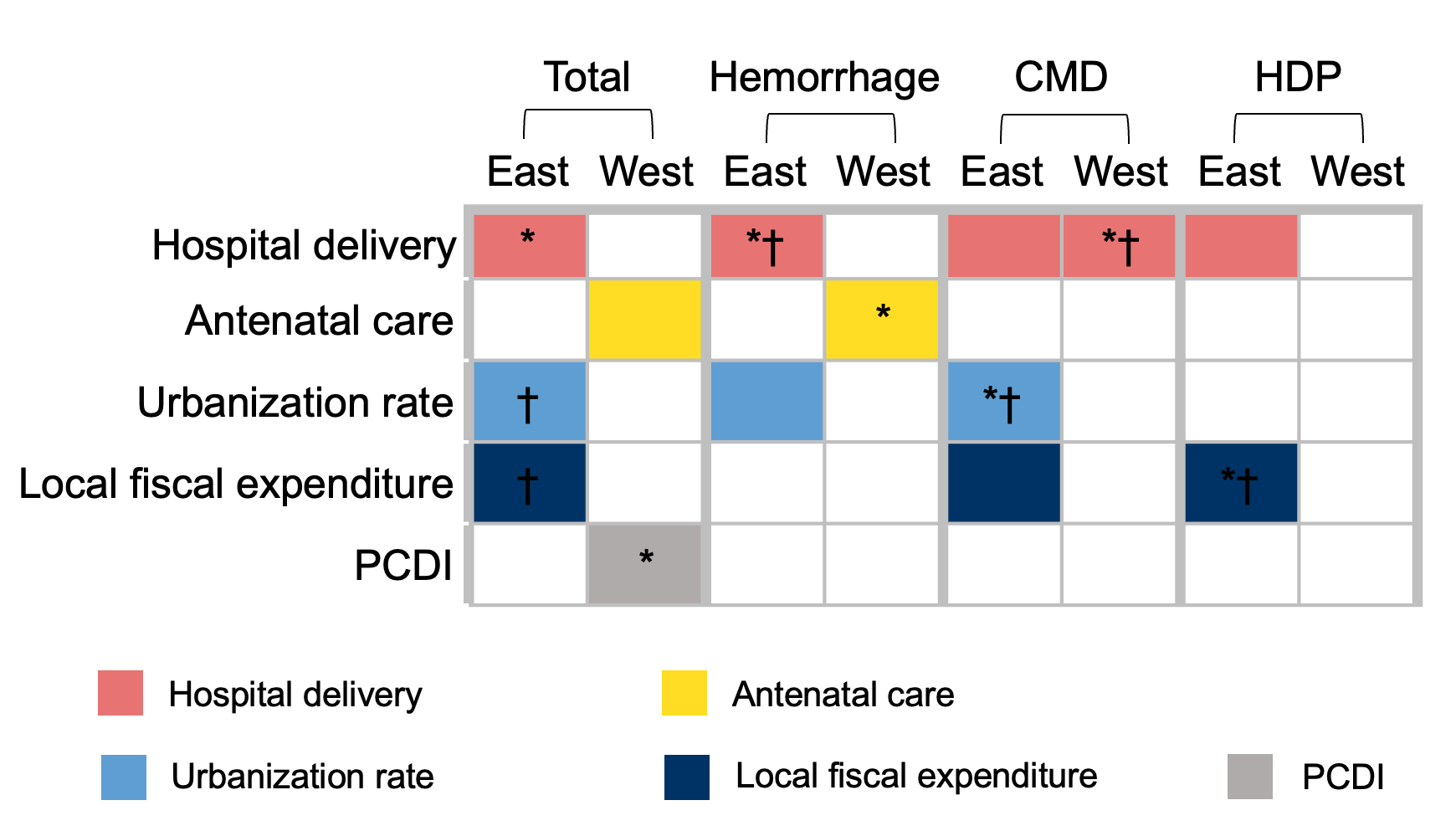

Supplement: S12 Fig — Note: The asterisk * and dagger † symbols in the cell represent that the factor contributes the most to the exposure–response relationship when all other factors are fixed at their 25th and 75th percentiles, respectively. White cell means that the factor is not identified as the component associated with reduced maternal mortality in the mixture. CMD, coexisting medical diseases; HDP, hypertensive disorders in pregnancy; PCDI, per capita. (TIFF) [file pmed.1004837.s028.tiff]

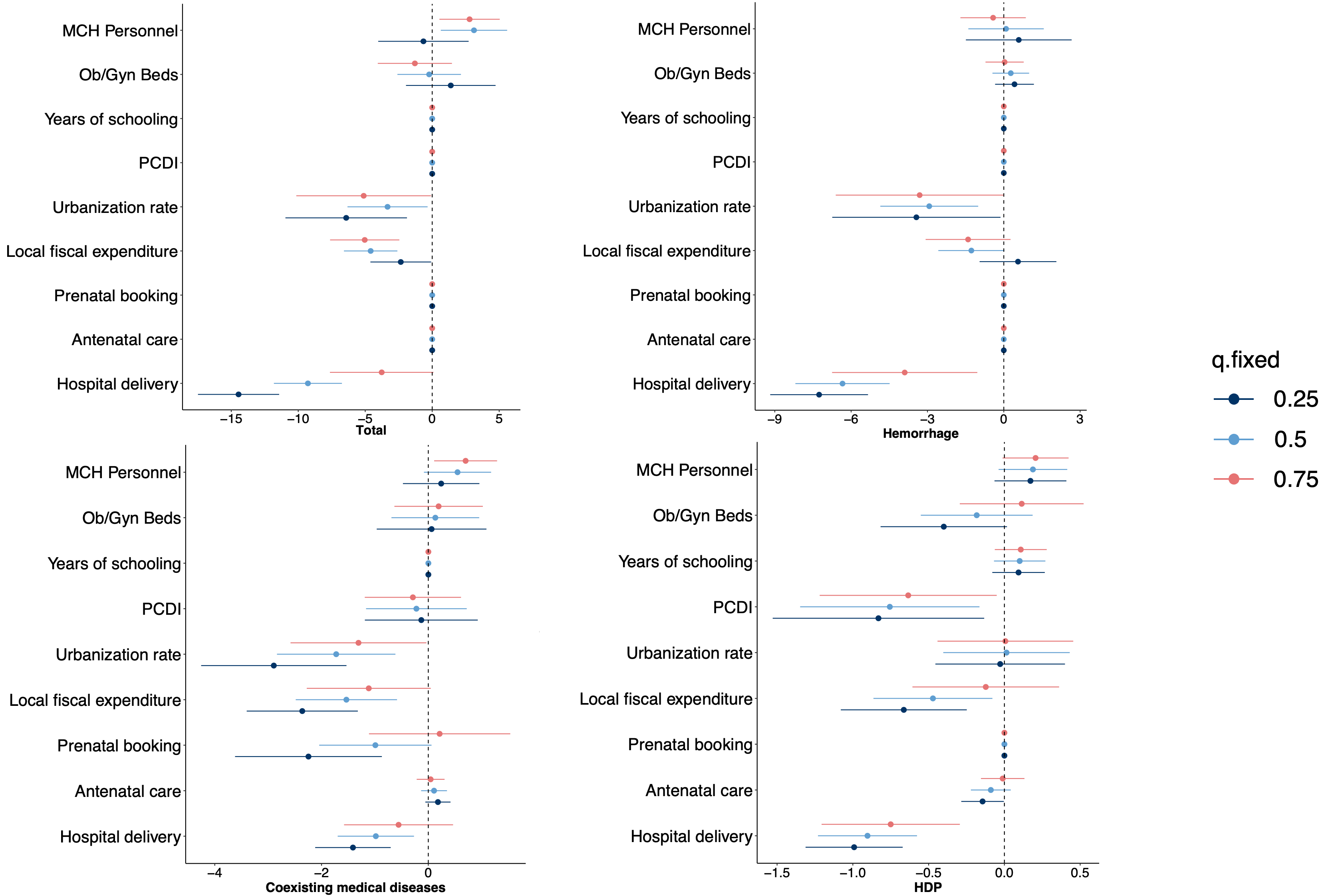

Supplement: S13 Fig — Note: The plot compares the exposure–response relationships associated with a change in a single exposure from the 75th percentile to the 25th percentile, when the other exposures are fixed at their 25th, 50th, and 75th percentiles. MCH, maternal and child health; Ob/Gyn, obstetrics and gynecology; PCDI, per capita disposable income; HDP, hypertensive disorders in pregnancy; q.fixed, quantiles at which to fix the remaining exposures. (TIFF) [file pmed.1004837.s029.tiff]

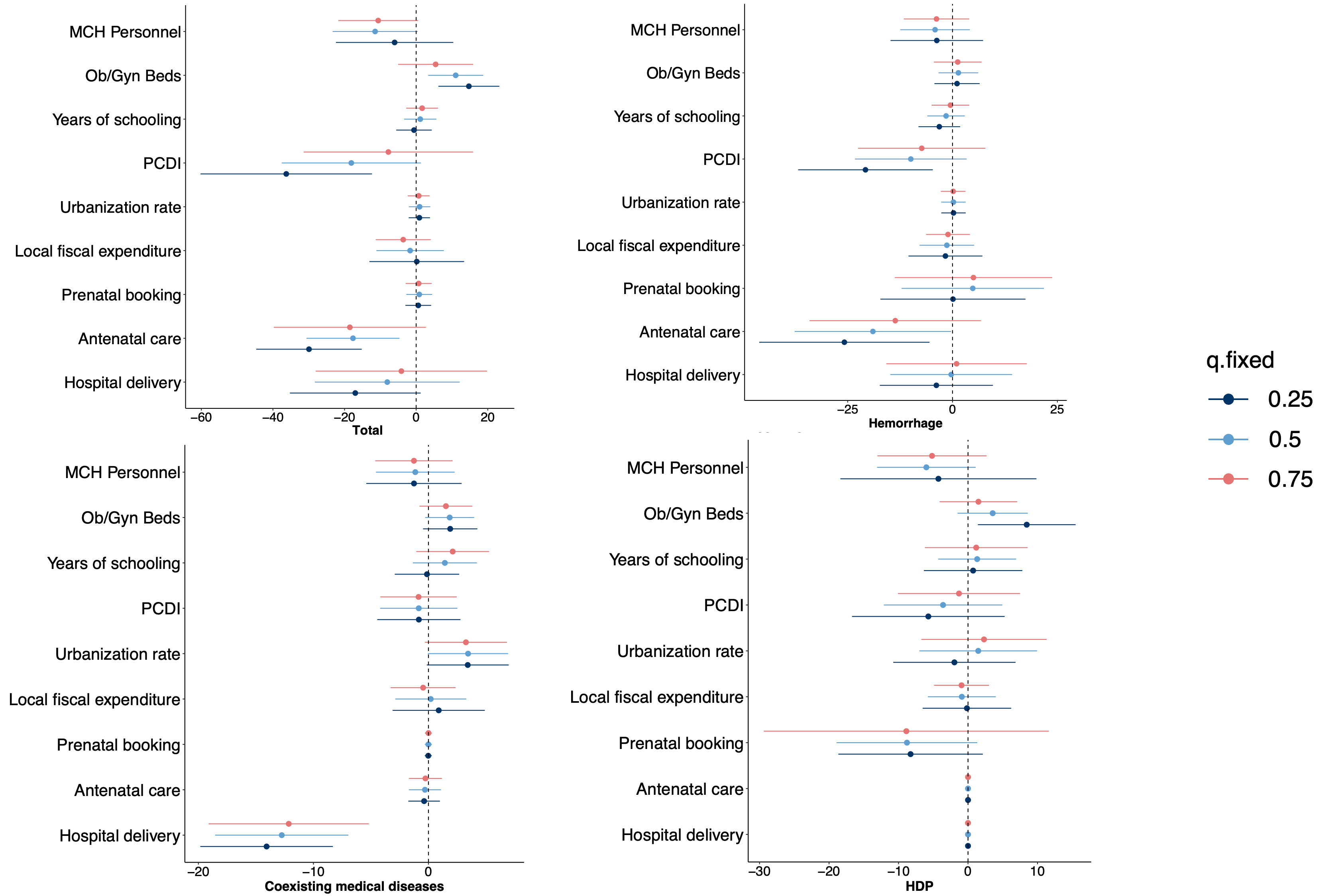

Supplement: S14 Fig — Note: The plot compares the exposure–response relationships associated with a change in a single exposure from the 75th percentile to the 25th percentile, when the other exposures are fixed at their 25th, 50th, and 75th percentiles. MCH, maternal and child health; Ob/Gyn, obstetrics and gynecology; PCDI, per capita disposable income; HDP, hypertensive disorders in pregnancy; q.fixed, quantiles at which to fix the remaining exposures. (TIFF) [file pmed.1004837.s030.tiff]
