# Supplementary material for: Social and health system factors associated with maternal mortality in Eastern and Western China: Population health estimates using provincial-level data
Source: PLoS Med. 2025 Dec 4;22(12):e1004837. doi: 10.1371/journal.pmed.1004837 (PMC12677549; doi:10.1371/journal.pmed.1004837)
Supplement: Attachment — Submitted filename: 20250928 Response letter.docx [file pmed.1004837.s032.docx]

Dear editor,

Thank you for the reviewers’ and editorial comments and suggestions concerning our manuscript entitled “Social and Health System Determinants of Maternal Mortality in More and Less Developed Regions of China: Implications for SDG 2030”. They are very helpful for improving our paper. We have reviewed them carefully and revised the paper thoroughly. The following is our point-by-point response to the reviewers’ comments and editorial requests.

***Comments from Reviewer 1:*** 

**This article uses Bayesian kernel machine regression to perform variable selection on various provincial-level social determinants of health to examine associations with longitudinal maternal mortality markers. In general, this was a very well-written article, with strong visualizations, and utilizing a statistical methodology that has been used before in similar settings. I appreciated the clarity in which the variables were defined, and the background provided on China's impressive improvements in maternal mortality and public health were helpful and provided great context for the study. However, there is potential room for improvement, as outlined in the suggestions below. I am not necessarily asking for entire reanalyses of the data or even that the suggestions be run, but would like to engage with the authors and understand more regarding the following points.**

Reply:

Thank you for your positive comments. Please see our point-by-point responses below.

**Comment 1:**

**I have two main comments: the first being that I didn't think the GP model was particularly necessary (in that use of this model and the interpretations that came out of it might be obfuscating simpler relationships), and the second being that some of the most relevant epidemiological interpretations weren't actually provided in terms of direct interpretations of exposure/response relationships. There were only nine predictors considered in the study, with "exposure group 2" comprising only a single variable! I'm curious what a model that simply used all predictors or one with sociologically-informed variable selection with groups would look like; my guess is pretty similar in terms of coefficient estimates to the one presented after the complex statistical machinery.**

Reply:

The reason why we used hierarchical BKMR models with GroupPIP and CondPIP outputs is that there is multicollinearity among these social and health system–related determinants. There are two approaches for variable selection in BKMR: the component-wise approach and the hierarchical approach. Unlike the component-wise approach, the hierarchical approach was specifically proposed to address collinearity among mixture components. The component-wise approach—that is, the model you mentioned that includes all predictors—treats components interchangeably and might fail to distinguish among these correlated components.

Results from component-wise BKMR models are as follows (Figs. R1-R5 and Tables R1-R4). The selected determinants and single-exposure risk measures did not change substantially, and the associations of some determinants with maternal mortality remained robust, such as hospital delivery and antenatal care. However, we consider the results obtained using the hierarchical approach to be relatively more reliable.

**Fig. R1 Determinants of total and cause-specific maternal mortality in eastern and western China during 2004-2012 and 2013-2020, using Bayesian Kernel Machine Regression component-wise variable selection.**

The asterisk * and dagger † symbols in the cell represent that the determinant contributes the most to the exposure-response relationship when all other determinants are fixed at their 25^th^ and 75^th^ percentiles, respectively. White cell represents that the determinant is not identified as the most important determinant of maternal mortality. CMD, coexisting medical diseases; HDP, hypertensive disorders in pregnancy; PCDI, per capita disposable income; Ob/Gyn, obstetrics and gynecology.

**Table R1 Posterior inclusion probabilities for each determinant in Eastern China, 2004-2012, using Bayesian Kernel Machine Regression component-wise variable selection.**

| **Exposure** | **Total maternal mortality** | **Maternal mortality due to hemorrhage** | **Maternal mortality due to coexisting medical diseases** | **Maternal mortality due to hypertensive disorders in pregnancy** |
| --- | --- | --- | --- | --- |
| Hospital delivery rate | 1 | 1 | 1 | 1 |
| Antenatal care rate | 0.978 | 0.996 | 0.526 | 0.987 |
| Prenatal booking rate | 0.957 | 0.997 | 0.993 | 0.833 |
| Local fiscal expenditure on healthcare | 0.559 | 0.811 | 0.485 | 0.941 |
| Urbanization rate | 1 | 1 | 1 | 0.963 |
| PCDI | 0.983 | 0.347 | 0.817 | 0.913 |
| Average years of schooling for females | 0.084 | 0.027 | 0.429 | 0.684 |
| Number of Ob/Gyn beds per 1000 livebirths | 0.574 | 0.696 | 0.828 | 0.980 |
| Number of MCH personnel per 1000 livebirths | 0.838 | 0.360 | 0.400 | 0.811 |

Note: MCH, maternal and child health; Ob/Gyn, obstetrics and gynecology; PCDI, per capita disposable income

**Table R2 Posterior inclusion probabilities for each determinant in Western China, 2004-2012, using Bayesian Kernel Machine Regression component-wise variable selection.**

| **Exposure** | **Total maternal mortality** | **Maternal mortality due to hemorrhage** | **Maternal mortality due to coexisting medical diseases** | **Maternal mortality due to hypertensive disorders in pregnancy** |
| --- | --- | --- | --- | --- |
| Hospital delivery rate | 0.909 | 0.949 | 0.945 | 1 |
| Antenatal care rate | 1 | 0.982 | 0.827 | 0.273 |
| Prenatal booking rate | 0.791 | 0.474 | 0.674 | 1 |
| Local fiscal expenditure on healthcare | 0.393 | 0.196 | 0.565 | 0.110 |
| Urbanization rate | 0.519 | 0.180 | 0.852 | 0.142 |
| PCDI | 0.956 | 0.631 | 0.828 | 0.132 |
| Average years of schooling for females | 0.989 | 0.694 | 0.918 | 0.101 |
| Number of Ob/Gyn beds per 1000 livebirths | 0.798 | 0.210 | 0.765 | 0.116 |
| Number of MCH personnel per 1000 livebirths | 0.527 | 0.154 | 0.514 | 0.127 |

Note: MCH, maternal and child health; Ob/Gyn, obstetrics and gynecology; PCDI, per capita disposable income

**Table R3 Posterior inclusion probabilities for each determinant in Eastern China, 2013-2020, using Bayesian Kernel Machine Regression component-wise variable selection.**

| **Exposure** | **Total maternal mortality** | **Maternal mortality due to hemorrhage** | **Maternal mortality due to coexisting medical diseases** | **Maternal mortality due to hypertensive disorders in pregnancy** |
| --- | --- | --- | --- | --- |
| Hospital delivery rate | 1 | 1 | 0.389 | 0.803 |
| Antenatal care rate | 0.913 | 0.123 | 0.375 | 0.965 |
| Prenatal booking rate | 0.251 | 0.161 | 0.152 | 0.235 |
| Local fiscal expenditure on healthcare | 0.254 | 0.186 | 0.777 | 0.499 |
| Urbanization rate | 0.682 | 0.993 | 0.470 | 0.653 |
| PCDI | 0.752 | 0.216 | 0.598 | 0.687 |
| Average years of schooling for females | 0.199 | 0.042 | 0.182 | 0.567 |
| Number of Ob/Gyn beds per 1000 livebirths | 0.263 | 0.185 | 0.253 | 0.913 |
| Number of MCH personnel per 1000 livebirths | 0.165 | 0.449 | 0.151 | 0.491 |

Note: MCH, maternal and child health; Ob/Gyn, obstetrics and gynecology; PCDI, per capita disposable income

**Table R4 Posterior inclusion probabilities for each determinant in Western China, 2013-2020, using Bayesian Kernel Machine Regression component-wise variable selection.**

| **Exposure** | **Total maternal mortality** | **Maternal mortality due to hemorrhage** | **Maternal mortality due to coexisting medical diseases** | **Maternal mortality due to hypertensive disorders in pregnancy** |
| --- | --- | --- | --- | --- |
| Hospital delivery rate | 1 | 1 | 0.618 | 0.562 |
| Antenatal care rate | 0.720 | 0.370 | 1 | 0.953 |
| Prenatal booking rate | 0.876 | 0.915 | 0.420 | 1 |
| Local fiscal expenditure on healthcare | 0.541 | 0.103 | 0.422 | 0.346 |
| Urbanization rate | 0.562 | 0.216 | 0.385 | 0.478 |
| PCDI | 0.328 | 0.344 | 0.239 | 0.500 |
| Average years of schooling for females | 0.442 | 0.169 | 0.811 | 0.482 |
| Number of Ob/Gyn beds per 1000 livebirths | 0.173 | 0.329 | 0.279 | 0.434 |
| Number of MCH personnel per 1000 livebirths | 0.509 | 0.300 | 0.273 | 0.529 |

Note: MCH, maternal and child health; Ob/Gyn, obstetrics and gynecology; PCDI, per capita disposable income

**Fig. R2 Single-exposure risk measures for each determinant in Eastern China, 2004-2012, using Bayesian Kernel Machine Regression component-wise variable selection.**

Note: The plot compares the exposure-response relationships associated with a change in a single exposure from the 75^th^ percentile to the 25^th^ percentile, when the other exposures are fixed at their 25^th^, 50^th^, and 75^th^ percentiles. MCH, maternal and child health; Ob/Gyn, obstetrics and gynecology; PCDI, per capita disposable income; HDP, hypertensive disorders in pregnancy; q.fixed, quantiles at which to fix the remaining exposures.

**Fig. R3 Single-exposure risk measures for each determinant in Western China, 2004-2012, using Bayesian Kernel Machine Regression component-wise variable selection.**

Note: The plot compares the exposure-response relationships associated with a change in a single exposure from the 75^th^ percentile to the 25^th^ percentile, when the other exposures are fixed at their 25th, 50th, and 75th percentiles. MCH, maternal and child health; Ob/Gyn, obstetrics and gynecology; PCDI, per capita disposable income; HDP, hypertensive disorders in pregnancy; q.fixed, quantiles at which to fix the remaining exposures.

**Fig. R4 Single-exposure risk measures for each determinant in Eastern China, 2013-2020, using Bayesian Kernel Machine Regression component-wise variable selection.**

Note: The plot compares the exposure-response relationships associated with a change in a single exposure from the 75^th^ percentile to the 25^th^ percentile, when the other exposures are fixed at their 25th, 50th, and 75th percentiles. MCH, maternal and child health; Ob/Gyn, obstetrics and gynecology; PCDI, per capita disposable income; HDP, hypertensive disorders in pregnancy; q.fixed, quantiles at which to fix the remaining exposures.

**Fig. R5 Single-exposure risk measures for each determinant in Western China, 2013-2020, using Bayesian Kernel Machine Regression component-wise variable selection.**

Note: The plot compares the exposure-response relationships associated with a change in a single exposure from the 75^th^ percentile to the 25^th^ percentile, when the other exposures are fixed at their 25th, 50th, and 75th percentiles. MCH, maternal and child health; Ob/Gyn, obstetrics and gynecology; PCDI, per capita disposable income; HDP, hypertensive disorders in pregnancy; q.fixed, quantiles at which to fix the remaining exposures.

In terms of the sociologically-informed variable selection, we have considered the sociological properties of the determinants when partitioning the 9 determinants in our primary analysis. To clarify this point, we have further revised the corresponding sentences in the Methods as follows (Page 10 Lines 204-211):

“Based on the correlation patterns and the sociologically-informed properties of the determinants, we partitioned the 9 determinants into 4 groups for the hierarchical variable selection in BKMR: 1) social determinants, including urbanization rate, PCDI, and average years of schooling; 2) health resources, including the number of MCH personnel and the number of Ob/Gyn beds; 3) health financing, including local fiscal expenditure on healthcare; and 4) coverage of maternal care, including prenatal booking rate, antenatal care rate, and hospital delivery rate.”

Under this consideration, local fiscal expenditure on healthcare was assigned to a separate group, “health financing”, which comprises only this determinant, because it does not fall under the domains of social determinants of health or maternal care coverage. Although it might be considered a health resource, its correlations with Ob/Gyn beds (Spearman correlation coefficient = 0.25) and MCH personnel (Spearman coefficient: 0.43) were weak. Therefore, we ultimately categorized local fiscal expenditure on healthcare as a standalone group.

As for the epidemiological interpretations of exposure/response relationships, we acknowledge that BKMR is less interpretable than traditional semi-parametric and parametric statistical models in providing a direct epidemiological interpretation with parsimonious parametric inference. To increase the interpretability as much as possible, we have added the data for Q1 and Q3 of the determinants when demonstrating the results of the single-exposure risk measures according to your Comment 2, and the sensitivity analysis using mixed-effects models according to your Comment 3. Please see our reply to your Comments 2 and 3 for details.

**Comment 2:**

**As mentioned, another potential area for improvement would be in interpretability of the model. Although it is perhaps of interest to know that an increase in hospital delivery rate from Q1 to Q3 "contributed the most to the exposure-response relationship," this provides only part of the picture. Is it possible that the antenatal care rate going from Q1 to Q3 also "contributes" quite a great deal to this relationship? What are the estimated posterior means and 95% credible sets there? Additionally, it would have been helpful to have some sort of real-world idea of what going from Q1 to Q3 entailed for this variable in the text instead of having to look it up in Table 1.**

Reply:

When conducting BKMR with hierarchical variable selection, the hospital delivery rate and antenatal care rate were grouped together based on their correlation patterns and their sociologically-informed properties. When the antenatal care rate had a smaller posterior inclusion probability (PIP) than the hospital delivery rate, we did not select the antenatal care rate as the variable of interest because it is less likely to be associated with maternal mortality than the hospital delivery rate. Indeed, when antenatal care rate had a smaller PIP, its single-exposure risk measures were non-significant or smaller than that of hospital delivery (Tables S2-S5, Figs. S3-S6). For example:

1) For total maternal mortality and maternal mortality due to hemorrhage and coexisting medical diseases in the East during 2004-2012, the maternal mortality due to coexisting medical diseases in the West during 2004-2012, maternal mortality due to hemorrhage in the East during 2013-2020, and the maternal mortality due to hemorrhage and hypertensive disorders in pregnancy in the West during 2013-2020, the CondPIPs for antenatal care rate were all 0, and the estimated posterior means and 95% credible sets associated with a change in antenatal care rate from its 75^th^ percentile to the 25^th^ percentile were all 0 (0, 0), when the other exposures fixed to their 25^th^, 50^th^, and 75^th^ percentile.
2) For maternal mortality due to hypertensive disorders in pregnancy in the East during 2004-2012, the CondPIP for antenatal care rate was 0.059, and the estimated posterior means and 95% credible sets were -0.52 (-0.84, -0.20), -0.32 (-0.60, -0.03), and -0.04 (-.34, 0.27) when the other exposures fixed to their 25^th^, 50^th^, and 75^th^ percentile, respectively. However, the estimated posterior means and 95% credible sets for the hospital delivery rate were -0.93 (-1.26, -0.60), -0.82 (-1.15, -0.49), and -0.66 (-1.12, -0.20) when the other exposures were fixed to their 25^th^, 50^th^, and 75^th^ percentiles, respectively.

Moreover, the objective of our study was to identify the most contributing factors for policy priority in the mixture of social and health system determinants; hence, secondary factors are not our primary interest.

As for the real-world idea of what going from Q1 to Q3 entailed for the variable, we have added the data in the Results as follows:

1) Page 14 Lines 305-307: “……an increase in hospital delivery rate from 91.7% to 99.7% percentile was associated with a higher value of the exposure-response relationship…...”
2) Pages 14-15 Lines 309-312: “……an increase in urbanization rate from 35.9% to 55.9% and an increase in local fiscal expenditure on healthcare from 9 billion to 21.9 billion Chinese Yuan were associated with larger responses in the decrease in maternal mortality……”
3) Page 15 Lines 316-320: “An increase in PCDI from 5500 to 9800 Chinese Yuan was associated with a larger single-exposure risk measure…… and an increase in antenatal care rate from 85.7% to 95.9% was associated with a higher response in the decrease in maternal mortality……”
4) Page 15 Lines 324-326: “……an increase in the urbanization rate from 52.7% to 67.4% was associated with a larger response when considering the single-exposure risk measures……”
5) Page 15 Lines 328-330: “……an increase in antenatal care rate from 94.7% to 97.7% was associated with a larger response when considering the single-exposure risk measures.”

**Comment 3:**

**On this note, the real-world implications of Q1 to Q3 changes also vary quite a bit. For instance, in Western China from 2004-2012, Q1-Q3 was 77.2 - 97.4 for hospital delivery rate, whereas in Eastern China from 2013-2020 that same Q1-Q3 was 99.9-100. To me, this seems to drive the story - there simply wasn't any meaningful variability in Eastern China in that time period, thus driving the storyline that hospital delivery "wasn't important" in explaining exposure/outcome relationships for that model. Would it not simply be more relevant to present results from a simpler model? Even a straightforward mixed-effects longitudinal model would be able to quantify something more directly relevant to public health/sociological understanding, something along the lines of "for this region in this time period, adjusting for xyz, each additional percentage of hospital delivery % was associated with an ##% reduction in maternal death due to hemorrhage" or something, instead of identifying the "most important variable" when going to Q1-Q3, knowing that this difference might be quite different across variables or even irrelevant in other cases (cf. the example earlier in this paragraph).**

Reply:

Thank you for your valuable comment. We acknowledge that the real-world implications of Q1 to Q3 are quite different across variables, and we have added the real-world data of what going from Q1 to Q3 entailed for each variable in the Results (Please see details in our reply to your Comment 2).

It is precisely because the real-world changes from Q1 to Q3 are so important that, when selecting the “important variables” in the BKMR model, we considered not only the PIP but also the contribution of an individual determinant to the response. Therefore, in Fig. 4 and in the Results, we did not merely highlight the variables with high PIP and significant single-exposure risk measures; we also emphasized the determinants contributing the most to the exposure–response relationship.

For total maternal mortality and maternal mortality due to hemorrhage in the East from 2013 to 2020, although hospital delivery rate had both a high PIP and a significant single-exposure risk measure, its contribution to the response was not as large as that of the urbanization rate. Consequently, we placed greater emphasis on the contribution of urbanization rate in the Results (e.g. “an increase in the urbanization rate from 52.7% to 67.4% was associated with a larger response when considering the single-exposure risk measures” in Page 15 Lines 324-326), rather than that of hospital delivery. Moreover, in Fig. 4A and 4B, we marked urbanization rate with both an asterisk and a dagger to emphasize its importance. The asterisk and dagger symbols represent that the determinant contributes the most to the exposure-response relationship when all other determinants are fixed at their 25th and 75th percentiles, respectively.

In terms of using a simpler model such as mixed-effects models, as we mentioned in the Introduction in Page 6 Lines 96-111, hierarchical BKMR addresses the possibility of collinearity by concurrently estimating both the importance of groups of highly correlated exposures or exposures from common sources, and the individual components within the group, making it more suitable for co-occurring exposure data than a mixed-effects model. However, BKMR cannot provide a direct epidemiological interpretation with parsimonious parametric inference. In order to quantify the parameter inference, we added the analyses using linear mixed-effects models in the sensitivity analysis. As suggested by your Comment 5 regarding the weights for different provinces, we added the sensitivity analyses of linear mixed-effects models weighted by the number of livebirths in each province for each year in the main text as follows. Results of models that are not weighted by the number of livebirths are shown in Table R5. Results from models weighted and not weighted by population did not show significant disparities.

1. In the Methods (Pages 11-12 Lines 251-259):

“Several sensitivity analyses were performed to test the robustness of our results. 1) BKMR is less interpretable than traditional semi-parametric and parametric statistical models in providing a direct epidemiological interpretation with parsimonious parametric inference. Therefore, we further applied linear mixed-effects models to investigate the parameter inference for the important components in the mixture of determinants identified by BKMR. The effect of each determinant was estimated using linear mixed-effects models adjusted for the other eight determinants and weighted by the number of livebirths in each province for each year.”

2. In the Results (Pages 16-17 Lines 358-371):

“Table S6 shows the results of linear mixed-effects models for important components in the mixture of determinants identified by BKMR, including hospital delivery rate, antenatal care rate, urbanization rate, local fiscal expenditure on healthcare, and PCDI. Consistent with the results in the primary analyses, hospital delivery rate was negatively associated with total and cause-specific maternal mortality in the East before 2013. When adjusting for the other eight determinants, each additional percentage of hospital delivery rate was associated with nearly one percent reduction in total maternal mortality and maternal death due to hemorrhage. In the West before 2013, each additional percentage of antenatal care rate was associated with 2% and 1% of reduction in maternal death due to hemorrhage and HDP, respectively, when adjusting for the other eight determinants. The observations for the urbanization rate and PCDI also aligned with the primary results.”

**Table S6 Effects of determinants on total maternal mortality, using linear mixed-effects models weighted by the number of livebirths.**

| Outcome | Determinant | 2004-2012 | | 2013-2020 | |
| --- | --- | --- | --- | --- | --- |
|  |  | East | West | East | West |
| Total maternal mortality | Hospital delivery rate | -1.09 (-1.22, -0.95) | -1.94 (-2.56, -1.31) | -3.09 (-4.05, -2.13) | -4.93 (-6.57, -3.29) |
|  | Antenatal care rate | -0.05 (-0.46, 0.36) | -1.90 (-3.97, 0.17) | -0.64 (-1.00, -0.29) | -0.96 (-1.97, 0.05) |
|  | Local fiscal expenditure on healthcare | -0.01 (-0.02, 0.01) | 0.07 (-0.07, 0.22) | 0 (0, 0.01) | -0.02 (-0.06, 0.02) |
|  | Urbanization rate | -0.27 (-0.42, -0.13) | 0.98 (0.22, 1.74) | -0.16 (-0.31, -0.01) | -0.60 (-1.47, 0.27) |
|  | Per capita disposable income | -1.42 (-5.78, 2.94) | -30.5 (-55.9, -5.18) | 0.21 (-1.05, 1.46) | 6.88 (-2.27, 16.02) |
| Maternal mortality due to hemorrhage | Hospital delivery rate | -0.90 (-0.99, -0.82) | -0.86 (-1.22, -0.5) | -2.16 (-2.52, -1.08) | -2.78 (-3.54, -2.02) |
|  | Antenatal care rate | -0.02 (-0.27, 0.23) | -2.01 (-3.11, -0.92) | -0.17 (-0.3, -0.04) | -1.05 (-1.61, -0.49) |
|  | Local fiscal expenditure on healthcare | 0 (-0.01, 0) | 0.04 (-0.03, 0.1) | 0 (0, 0) | -0.01 (-0.02, 0) |
|  | Urbanization rate | -0.14 (-0.23, -0.06) | -0.01 (-0.44, 0.43) | -0.11 (-0.16, -0.06) | -0.36 (-0.64, -0.08) |
|  | Per capita disposable income | 1.11 (-1.58, 3.79) | -3.4 (-18.0, 11.1) | 0.56 (0.11, 1.01) | 3.57 (-0.35, 7.49) |
| Maternal mortality due to coexisting medical diseases | Hospital delivery rate | -0.16 (-0.21, -0.11) | -0.59 (-0.80, -0.37) | -0.39 (-0.87, 0.08) | -0.58 (-1.20, 0.04) |
|  | Antenatal care rate | 0.09 (-0.05, 0.22) | -0.37 (-1.04, 0.3) | -0.04 (-0.21, 0.12) | 0.01 (-0.4, 0.42) |
|  | Local fiscal expenditure on healthcare | 0 (-0.01, 0) | 0 (-0.05, 0.05) | 0 (0, 0) | -0.02 (-0.03, -0.01) |
|  | Urbanization rate | -0.10 (-0.15, -0.06) | 0.48 (0.22, 0.75) | -0.03 (-0.09, 0.03) | -0.27 (-0.54, 0) |
|  | Per capita disposable income | -0.45 (-1.95, 1.05) | -8.51 (-17.4, 0.41) | -0.13 (-0.66, 0.41) | 5.14 (1.77, 8.52) |
| Maternal mortality due to hypertensive disorders in pregnancy | Hospital delivery rate | -0.10 (-0.12, -0.08) | -0.38 (-0.63, -0.12) | -0.14 (-0.36, 0.07) | -1.13 (-1.71, -0.55) |
|  | Antenatal care rate | 0.02 (-0.04, 0.08) | -1.05 (-1.83, -0.26) | -0.08 (-0.16, -0.01) | -0.12 (-0.52, 0.27) |
|  | Local fiscal expenditure on healthcare | 0 (0, 0) | 0.05 (0, 0.1) | 0 (0, 0) | 0 (-0.01, 0.01) |
|  | Urbanization rate | -0.03 (-0.05, -0.01) | 0.26 (-0.05, 0.57) | -0.03 (-0.05, 0) | -0.06 (-0.31, 0.18) |
|  | Per capita disposable income | 0.01 (-0.66, 0.68) | -7.63 (-18.2, 2.91) | -0.04 (-0.29, 0.21) | 1.45 (-1.69, 4.59) |

Note: Effects are shown as estimates and the 95% confidence intervals in the linear mixed effects model adjusting for the other eight determinants

3. Table R5 Effects of determinants on total maternal mortality, using linear mixed-effects models not weighted by the number of livebirths.

| Outcome | Determinant | 2004-2012 | | 2013-2020 | |
| --- | --- | --- | --- | --- | --- |
|  |  | East | West | East | West |
| Total maternal mortality | Hospital delivery rate | -1.12 (-1.25, -0.98) | -2.26 (-2.96, -1.55) | -3.18 (-4.45, -1.91) | -4.73 (-6.26, -3.19) |
|  | Antenatal care rate | -0.02 (-0.35, 0.30) | -0.47 (-2.36, 1.42) | -0.56 (-0.96, -0.15) | -1.29 (-2.08, -0.51) |
|  | Local fiscal expenditure on healthcare | -0.01 (-0.02, 0) | 0.04 (-0.14, 0.21) | 0 (0, 0) | -0.03 (-0.07, 0.02) |
|  | Urbanization rate | -0.27 (-0.41, -0.14) | 1.10 (0.06, 2.15) | -0.11 (-0.25, 0.03) | -0.86 (-1.82, 0.11) |
|  | Per capita disposable income | -0.27 (-3.40, 2.87) | -23.6 (-58.2, 11.0) | -0.62 (-1.69, 0.46) | 12.5 (1.57, 23.4) |
| Maternal mortality due to hemorrhage | Hospital delivery rate | -0.89 (-0.97, -0.81) | -1.33 (-1.76, -0.89) | -2.30 (-2.74, -1.86) | -2.50 (-3.26, -1.74) |
|  | Antenatal care rate | -0.02 (-0.21, 0.18) | -2.02 (-3.05, -0.99) | -0.13 (-0.27, 0) | -1.42 (-1.92, -0.91) |
|  | Local fiscal expenditure on healthcare | -0.01 (-0.01, 0) | 0.05 (-0.03, 0.13) | 0 (0, 0) | -0.01 (-0.02, 0.01) |
|  | Urbanization rate | -0.14 (-0.22, -0.06) | 0.37 (-0.26, 1.01) | -0.08 (-0.12, -0.03) | -0.44 (-0.8, -0.08) |
|  | Per capita disposable income | 1.41 (-0.47, 3.30) | -3.75 (-24.4, 16.9) | 0.27 (-0.09, 0.64) | 4.31 (-1.02, 9.64) |
| Maternal mortality due to coexisting medical diseases | Hospital delivery rate | -0.17 (-0.22, -0.12) | -0.71 (-0.95, -0.46) | -0.55 (-1.14, 0.05) | -0.79 (-1.35, -0.22) |
|  | Antenatal care rate | 0.11 (-0.02, 0.23) | -0.02 (-0.63, 0.60) | -0.06 (-0.24, 0.12) | 0.12 (-0.23, 0.47) |
|  | Local fiscal expenditure on healthcare | -0.01 (-0.01, 0) | 0 (-0.06, 0.05) | 0 (0, 0) | -0.02 (-0.03, 0) |
|  | Urbanization rate | -0.11 (-0.17, -0.06) | 0.58 (0.2, 0.96) | -0.02 (-0.07, 0.04) | -0.38 (-0.68, -0.08) |
|  | Per capita disposable income | 0.24 (-1.00, 1.49) | -6.95 (-19.1, 5.18) | -0.22 (-0.68, 0.25) | 6.70 (2.66, 10.75) |
| Maternal mortality due to hypertensive disorders in pregnancy | Hospital delivery rate | -0.10 (-0.13, -0.08) | -0.42 (-0.78, -0.07) | -0.21 (-0.51, 0.09) | -1.28 (-1.77, -0.80) |
|  | Antenatal care rate | 0.03 (-0.03, 0.09) | -0.79 (-1.64, 0.07) | -0.13 (-0.22, -0.03) | -0.04 (-0.37, 0.28) |
|  | Local fiscal expenditure on healthcare | 0 (0, 0) | 0.06 (-0.01, 0.14) | 0 (0, 0) | -0.01 (-0.02, 0.01) |
|  | Urbanization rate | -0.02 (-0.05, 0) | 0.23 (-0.31, 0.78) | -0.02 (-0.04, 0.01) | -0.14 (-0.37, 0.09) |
|  | Per capita disposable income | -0.11 (-0.69, 0.46) | -5.27 (-22.5, 12.0) | -0.07 (-0.32, 0.17) | 3.46 (0.07, 6.86) |

Note: Effects are shown as estimates and the 95% confidence intervals in the linear mixed effects model adjusting for the other eight determinants.

**Comment 4:**

**An observation regarding the modeling procedure is that fitting the Eastern/Western separately removes ability to borrow information (which the Bayesian paradigm is great for) across provinces in the other region, essentially fitting interactions across all variables. This is fine if this was the intention, but I also wonder what "one big model" with all provinces and regions would have looked like together, perhaps with terms/interactions corresponding to region.**

Reply:

As we mentioned in the Methods (Page 9, Lines 182-186), there is a significant disparity in socioeconomic development between the eastern and western regions of China. Besides, the regional differences in maternal mortality existed between 2004 and 2020 (Page 12 Lines 275-280). Therefore, a “one big model” that combines all provinces might obscure the unique exposure-response relationships of each region. Additionally, due to the spatio-temporal variation of maternal mortality, fitting the models in different years and regions separately might enable other LMICs to draw lessons from the phases where China’s situation is comparable to their current situation.

Results from the models that include all provinces are shown in Figs. R6-R8 and Tables R6-R7. We are unable to add interactions corresponding to the region in the BKMR model. In BKMR, interaction can only be computed for different exposures. Similar to the results in our primary analyses, hospital delivery rate, urbanization rate, and PCDI were identified as important components in the mixture of determinants. However, the single-exposure risk measures for antenatal care rate were not significant, and the GroupPIP for local fiscal expenditure on healthcare was lower than 0.9.

**Fig. R6 Determinants of total and cause-specific maternal mortality in China during 2004-2012 and 2013-2020.**

The asterisk * and dagger † symbols in the cell represent that the determinant contributes the most to the exposure-response relationship when all other determinants are fixed at their 25^th^ and 75^th^ percentiles, respectively. White cell means that the determinant is not identified as the most important determinant of maternal mortality. HEM, hemorrhage; CMD, coexisting medical diseases; HDP, hypertensive disorders in pregnancy; PCDI, per capita disposable income; MCH, maternal and child health.

**Table R6 Group and conditional posterior inclusion probabilities for each determinant in China, 2004-2012, using Bayesian Kernel Machine Regression hierarchical variable selection.**

| **Exposure** | **Exposure group** | **Total maternal mortality** | | **Maternal mortality due to hemorrhage** | | **Maternal mortality due to coexisting medical diseases** | | **Maternal mortality due to hypertensive disorders in pregnancy** | |
| --- | --- | --- | --- | --- | --- | --- | --- | --- | --- |
|  |  | **GroupPIP** | **CondPIP** | **GroupPIP** | **CondPIP** | **GroupPIP** | **CondPIP** | **GroupPIP** | **CondPIP** |
| Hospital delivery rate | 1 | 1 | 1 | 1 | 1 | 1 | 1 | 1 | 1 |
| Antenatal care rate | 1 | 1 | 0 | 1 | 0 | 1 | 0 | 1 | 0 |
| Prenatal booking rate | 1 | 1 | 0 | 1 | 0 | 1 | 0 | 1 | 0 |
| Local fiscal expenditure on healthcare | 2 | 0.834 | 1 | 0.503 | 1 | 0.071 | 1 | 0 | 0 |
| Urbanization rate | 3 | 1 | 0.752 | 1 | 0 | 1 | 0 | 1 | 0 |
| PCDI | 3 | 1 | 0 | 1 | 0 | 1 | 0 | 1 | 0 |
| Average years of schooling for females | 3 | 1 | 0.248 | 1 | 1 | 1 | 1 | 1 | 1 |
| Number of Ob/Gyn beds per 1000 livebirths | 4 | 0.990 | 0.0004 | 0.918 | 0 | 0.159 | 0.918 | 0.593 | 0 |
| Number of MCH personnel per 1000 livebirths | 4 | 0.990 | 1 | 0.918 | 1 | 0.159 | 0.082 | 0.593 | 1 |

Note: GroupPIP, group posterior inclusion probabilities; CondPIP, conditional posterior inclusion probabilities; MCH, maternal and child health; Ob/Gyn, obstetrics and gynecology; PCDI, per capita disposable income.

**Table R7 Group and conditional posterior inclusion probabilities for each determinant in China, 2013-2020, using Bayesian Kernel Machine Regression hierarchical variable selection.**

| **Exposure** | **Exposure group** | **Total maternal mortality** | | **Maternal mortality due to hemorrhage** | | **Maternal mortality due to coexisting medical diseases** | | **Maternal mortality due to hypertensive disorders in pregnancy** | |
| --- | --- | --- | --- | --- | --- | --- | --- | --- | --- |
|  |  | **GroupPIP** | **CondPIP** | **GroupPIP** | **CondPIP** | **GroupPIP** | **CondPIP** | **GroupPIP** | **CondPIP** |
| Hospital delivery rate | 1 | 1 | 1 | 1 | 1 | 1 | 1 | 1 | 1 |
| Antenatal care rate | 1 | 1 | 0 | 1 | 0 | 1 | 0 | 1 | 0 |
| Prenatal booking rate | 1 | 1 | 0 | 1 | 0 | 1 | 0 | 1 | 0 |
| Local fiscal expenditure on healthcare | 2 | 0.074 | 1 | 0.0004 | 1 | 0.845 | 1 | 0.020 | 1 |
| Urbanization rate | 3 | 1 | 0 | 1 | 0 | 0.355 | 0.208 | 0.135 | 0.343 |
| PCDI | 3 | 1 | 1 | 1 | 0 | 0.355 | 0.592 | 0.135 | 0.228 |
| Average years of schooling for females | 3 | 1 | 0 | 1 | 1 | 0.355 | 0.200 | 0.135 | 0.429 |
| Number of Ob/Gyn beds per 1000 livebirths | 4 | 0.360 | 0 | 1 | 0 | 0.068 | 0.102 | 1 | 0.0004 |
| Number of MCH personnel per 1000 livebirths | 4 | 0.360 | 1 | 1 | 1 | 0.068 | 0.898 | 1 | 1 |

Note: GroupPIP, group posterior inclusion probabilities; CondPIP, conditional posterior inclusion probabilities; MCH, maternal and child health; Ob/Gyn, obstetrics and gynecology; PCDI, per capita disposable income.

**Fig. R7 Single-exposure risk measures for each determinant in China, 2004-2012, using Bayesian Kernel Machine Regression hierarchical variable selection.**

Note: The plot compares the exposure-response relationships associated with a change in a single exposure from the 75^th^ percentile to the 25^th^ percentile, when the other exposures are fixed at their 25th, 50th, and 75th percentiles. MCH, maternal and child health; Ob/Gyn, obstetrics and gynecology; PCDI, per capita disposable income; HDP, hypertensive disorders in pregnancy; q.fixed, quantiles at which to fix the remaining exposures.

**Fig. R8 Single-exposure risk measures for each determinant in China, 2013-2020, using Bayesian Kernel Machine Regression hierarchical variable selection.**

Note: The plot compares the exposure-response relationships associated with a change in a single exposure from the 75^th^ percentile to the 25^th^ percentile, when the other exposures are fixed at their 25th, 50th, and 75th percentiles. MCH, maternal and child health; Ob/Gyn, obstetrics and gynecology; PCDI, per capita disposable income; HDP, hypertensive disorders in pregnancy; q.fixed, quantiles at which to fix the remaining exposures.

**Comment 5:**

**As a personal curiosity, I also wonder what an analysis weighted by population would look like - as is, Qinghai has the same weight as Sichuan in the model, though on absolute public health terms regarding mortality, it seems that we might care much more about Sichuan or that its contribution should be weighted much more. It may not be worth running the model, but a sensitivity analysis (perhaps of a simple mixed-effects model) may be interesting from a scientific point of view.**

Reply:

Thank you for your valuable suggestion. We have added the sensitivity analysis using a mixed-effects model weighted by the number of livebirths in each province for each year in the main text. Please see our reply to your Comment 3 for details.

**Comment 6:**

**Finally, use of words like "attributable" or "most important" may unintentionally imply a causal relationship with these social determinants of health and reduced maternal mortality despite these being retrospective observational data. I would strongly encourage you to use more associational language in order not to inadvertently mislead less savvy readers.**

Reply:

We have revised the words like “attributable” or “most important” as more associational language such as “important determinants negatively associated with maternal mortality”, “correlated with the decreases in maternal mortality”, “relatively important determinant for”, “the most important components in the mixture of determinants”, “determinants with larger GroupPIP and CondPIP, and significant single-exposure risk measures”, “associated with a higher value of the exposure-response relationship”:

1. **Abstract:**

Pages 2-3 Lines 16-31 : “**Methods and findings** ……Hospital delivery rate, antenatal care rate, urbanization rate, local fiscal expenditure on healthcare, and per capita disposable income (PCDI) were identified as important determinants negatively associated with maternal mortality during the 2004-2020 period. In the East, hospital delivery correlated with the decreases in total maternal mortality and maternal deaths due to hemorrhage and hypertensive disorders before 2013, and the urbanization rate was negatively associated with total and hemorrhage-related maternal mortality after 2013. Local fiscal expenditure on healthcare and antenatal care were also important determinants in the East. In the West, the antenatal care rate was a relatively important determinant for reducing total maternal mortality before and after 2013. PCDI and hospital delivery were also important determinants in the West.

**Conclusions** Coverage of maternal care, health financing and urbanization were important determinants associated with the substantial reduction in maternal deaths in eastern and western China during 2004-2020……”

2. **Methods:**

Page 11 Lines 244-250: “We used the following criteria to identify the most important components in the mixture of determinants: 1) Among the 4 groups of determinants, we first identified the most important groups using a GroupPIP threshold of 0.9; 2) For a group with a GroupPIP ≥ 0.9, the most important component within the group was the one with the highest CondPIP; 3) For the most important component within a group, significant and negative estimate of the single-exposure risk measures should be observed.”

3. **Results:**
1) Page 14 Lines 296-298: “Using BKMR, five determinants were identified as important contributors associated with the decreased maternal mortality based on the results of GroupPIP, CondPIP, and single-exposure risk measures……”
2) Page 14 Lines 301-308: “For the reduction of total maternal mortality before 2013, the determinants with larger GroupPIP and CondPIP, and significant single-exposure risk measures in the East were hospital delivery rate, urbanization rate, and local fiscal expenditure on healthcare……an increase in hospital delivery rate from 91.7% to 99.7% percentile was associated with a higher value of the exposure-response relationship with a posterior mean and standard deviation (SD) of -14.8(1.5).”
3) Page 15 Lines 314-315 & 319-324 & 327-328: “Before 2013, the relatively important determinants identified by BKMR in the western region were PCDI and antenatal care rate……an increase in antenatal care rate from 85.7% to 95.9% was associated with a higher response in the decrease in maternal mortality when other determinants were at a higher level….. urbanization rate and hospital delivery rate had higher GroupPIP and CondPIP, and significant single-exposure risk measures for the reduction of total maternal mortality...... The antenatal care rate was the relatively important determinant identified by BKMR in the West after 2013…...”
4) Pages 15-16 Lines 331-340: “The reduction of deaths due to hemorrhage was mainly correlated with hospital delivery rate, urbanization rate, and local fiscal expenditure on healthcare in the East during 2004-2012 with hospital delivery rate as the top contributor in the exposure-response relationships, and hospital delivery rate and urbanization rate during 2013-2020 with urbanization rate as the relatively important component in the mixture of determinants….. antenatal care rate and hospital delivery rate were the important determinants with larger GroupPIP and CondPIP, and significant single-exposure risk measures before and after 2013, respectively.”
5) Page 16 Lines 342-347: “the urbanization rate and local fiscal expenditure on healthcare were the relatively important components in the mixture of determinants before and after 2013, respectively……hospital delivery rate was also an important determinant associated with the decreased maternal mortality due to coexisting medical diseases in both the East and the West.”
6) Page 16 Lines 349-354: “Hospital delivery rate and local fiscal expenditure on healthcare were the determinants with larger GroupPIP and CondPIP, and significant single-exposure risk measures for the decline in deaths due to HDP in the East before 2013, and hospital delivery rate was associated with a larger single-exposure risk measure……Antenatal care rate was a relatively important component in the mixture of determinants in the West……”
4. **Discussion**:
1) Page 17 Lines 382-385: “We found that hospital delivery, antenatal care, urbanization, local fiscal expenditure on healthcare, and PCDI were important determinants associated with the remarkable progress in maternal survival in both the eastern and western regions during this period.”
2) Page 17 Lines 389-392: “In our mixture analysis considering the simultaneous effects of multiple distal determinants, the coverage of maternal care, including antenatal care and hospital delivery, was a key determinant associated with the decline in maternal deaths in China.”
3) Page 23 Lines 551-554: “Although the social and health system determinants are all crucial to maternal mortality, investigating the relative importance of these determinants can provide practical guidance for policy and program planning in these developed regions.”

**Comment 7:**

**As minor suggestions, I would have appreciated a bit more information regarding the imputation approach for missing determinants of health (what specific model was used? How much data was missing? Why not an imputation method that allows for valid inference, e.g., MICE, instead of deterministic imputation according to a model, thus under-representing variability both in terms of the variable itself but also through the fact that imputation had to be performed?).**

Reply:

We have added the following details regarding the imputation approach in the Methods (Pages 9-10 Lines 196-203):

“The following data were missing for all provinces: local fiscal expenditure on healthcare between 2004 and 2006, per capita disposable income in 2004, and the number of health technical personnel in maternal and child health care per 1000 livebirths in 2009. For missing data imputation, we fitted a linear regression model for year and each of these three determinants in each province. We used linear regression estimate imputation because the socio-economic missing value has a correlation with time and is at the beginning of the time [14].

Reference: 14. Wubetie, H.T. Missing data management and statistical measurement of socio-economic status: application of big data. Journal of Big Data. 2017;4(1):47.”

We also added the sensitivity analysis using MICE as follows:

1. In the Methods (Page 12 Lines 263-268):

“3) We used another method, multivariate imputation by chained equations (MICE) algorithm, to impute the missing values of exposures in 2004-2006 and 2009. Five imputed datasets were generated and modeled separately to re-examine the determinants of maternal mortality in the East and West between 2004 and 2012. Results were pooled to obtain the final effect parameters.”

2. In the Results (Page 17 Lines 375-377):

“When using datasets with imputations for missing data by MICE, the associations between determinants and outcomes remained robust (Figs. S12-S14, Tables S11-S12).”

**Fig. S12 Determinants of total and cause-specific maternal mortality in eastern and western China during 2004-2012, using Bayesian Kernel Machine Regression hierarchical variable selection with missing data imputed by MICE.**

Note: The asterisk * and dagger † symbols in the cell represent that the determinant contributes the most to the exposure-response relationship when all other determinants are fixed at their 25^th^ and 75^th^ percentiles, respectively. White cell represents that the determinant is not identified as the most important determinant of maternal mortality. CMD, coexisting medical diseases; HDP, hypertensive disorders in pregnancy; PCDI, per capita.

**Table S11 Group and conditional posterior inclusion probabilities for each determinant in Eastern China, 2004-2012, using Bayesian Kernel Machine Regression hierarchical variable selection with missing data imputed by MICE.**

| **Exposure** | **Exposure group** | **Total maternal mortality** | | **Maternal mortality due to hemorrhage** | | **Maternal mortality due to coexisting medical diseases** | | **Maternal mortality due to hypertensive disorders in pregnancy** | |
| --- | --- | --- | --- | --- | --- | --- | --- | --- | --- |
|  |  | **GroupPIP** | **CondPIP** | **GroupPIP** | **CondPIP** | **GroupPIP** | **CondPIP** | **GroupPIP** | **CondPIP** |
| Hospital delivery rate | 1 | 1 | 1 | 1 | 1 | 1 | 0.768 | 1 | 0.990 |
| Antenatal care rate | 1 | 1 | 0 | 1 | 0 | 1 | 0.002 | 1 | 0.010 |
| Prenatal booking rate | 1 | 1 | 0 | 1 | 0 | 1 | 0.230 | 1 | 0 |
| Local fiscal expenditure on healthcare | 2 | 0.989 | 1 | 0.998 | 1 | 0.900 | 1 | 0.928 | 1 |
| Urbanization rate | 3 | 1 | 1 | 1 | 1 | 0.991 | 0.921 | 0.739 | 0.268 |
| PCDI | 3 | 1 | 0 | 1 | 0 | 0.991 | 0.079 | 0.739 | 0.710 |
| Average years of schooling for females | 3 | 1 | 0 | 1 | 0 | 0.991 | 0 | 0.739 | 0.022 |
| Number of Ob/Gyn beds per 1000 livebirths | 4 | 0.825 | 0.066 | 0.994 | 0.013 | 0.673 | 0.683 | 0.603 | 0.913 |
| Number of MCH personnel per 1000 livebirths | 4 | 0.825 | 0.934 | 0.994 | 0.987 | 0.673 | 0.317 | 0.603 | 0.087 |

Note: GroupPIP, group posterior inclusion probabilities; CondPIP, conditional posterior inclusion probabilities; MCH, maternal and child health; Ob/Gyn, obstetrics and gynecology; PCDI, per capita disposable income.

**Table S12 Group and conditional posterior inclusion probabilities for each determinant in Western China, 2004-2012, using Bayesian Kernel Machine Regression hierarchical variable selection with missing data imputed by MICE.**

| **Exposure** | **Exposure group** | **Total maternal mortality** | | **Maternal mortality due to hemorrhage** | | **Maternal mortality due to coexisting medical diseases** | | **Maternal mortality due to hypertensive disorders in pregnancy** | |
| --- | --- | --- | --- | --- | --- | --- | --- | --- | --- |
|  |  | **GroupPIP** | **CondPIP** | **GroupPIP** | **CondPIP** | **GroupPIP** | **CondPIP** | **GroupPIP** | **CondPIP** |
| Hospital delivery rate | 1 | 1 | 0.178 | 1 | 0.307 | 0.999 | 0.987 | 1 | 0 |
| Antenatal care rate | 1 | 1 | 0.811 | 1 | 0.572 | 0.999 | 0.009 | 1 | 0 |
| Prenatal booking rate | 1 | 1 | 0.011 | 1 | 0.121 | 0.999 | 0.004 | 1 | 1 |
| Local fiscal expenditure on healthcare | 2 | 0.401 | 1 | 0.235 | 1 | 0.388 | 1 | 0.244 | 1 |
| Urbanization rate | 3 | 0.969 | 0.009 | 0.870 | 0.016 | 0.556 | 0.240 | 0.934 | 0.536 |
| PCDI | 3 | 0.969 | 0.884 | 0.870 | 0.875 | 0.556 | 0.142 | 0.934 | 0.294 |
| Average years of schooling for females | 3 | 0.969 | 0.106 | 0.870 | 0.108 | 0.556 | 0.618 | 0.934 | 0.170 |
| Number of Ob/Gyn beds per 1000 livebirths | 4 | 0.703 | 0.543 | 0.312 | 0.423 | 0.342 | 0.495 | 0.585 | 0.563 |
| Number of MCH personnel per 1000 livebirths | 4 | 0.703 | 0.457 | 0.312 | 0.577 | 0.342 | 0.505 | 0.585 | 0.437 |

Note: GroupPIP, group posterior inclusion probabilities; CondPIP, conditional posterior inclusion probabilities; MCH, maternal and child health; Ob/Gyn, obstetrics and gynecology; PCDI, per capita disposable income.

**Fig. S13 Single-exposure risk measures for each determinant in Eastern China, 2004-2012, using Bayesian Kernel Machine Regression hierarchical variable selection with missing data imputed by MICE.**

Note: The plot compares the exposure-response relationships associated with a change in a single exposure from the 75^th^ percentile to the 25^th^ percentile, when the other exposures are fixed at their 25th, 50th, and 75th percentiles. MCH, maternal and child health; Ob/Gyn, obstetrics and gynecology; PCDI, per capita disposable income; HDP, hypertensive disorders in pregnancy; q.fixed, quantiles at which to fix the remaining exposures.

**Fig. S14 Single-exposure risk measures for each determinant in Western China, 2004-2012, using Bayesian Kernel Machine Regression hierarchical variable selection with missing data imputed by MICE.**

Note: The plot compares the exposure-response relationships associated with a change in a single exposure from the 75^th^ percentile to the 25^th^ percentile, when the other exposures are fixed at their 25th, 50th, and 75th percentiles. MCH, maternal and child health; Ob/Gyn, obstetrics and gynecology; PCDI, per capita disposable income; HDP, hypertensive disorders in pregnancy; q.fixed, quantiles at which to fix the remaining exposures.

**Comment 8:**

**Furthermore, the term "posterior SD" does not seem to apply (e.g., in line 259) - my guess is that you are presenting 95% HPD credible intervals for the posterior mean.**

Reply:

We have changed the numbers in parentheses to SD as follows (Pages 14-15 Lines 304-314 & 316-321): “When all other determinants were at a lower level (25th percentile), an increase in hospital delivery rate from 91.7% to 99.7% percentile was associated with a higher value of the exposure-response relationship with a posterior mean and standard deviation (SD) of -14.8(1.5) (Fig. S3). When all other determinants reached a high level (75th percentile), an increase in urbanization rate from 35.9% to 55.9% and an increase in local fiscal expenditure on healthcare from 9 billion to 21.9 billion Chinese Yuan were associated with larger responses in the decrease in maternal mortality [posterior mean and SD for urbanization rate: -5.8(2.6); posterior mean and SD for local fiscal expenditure on healthcare: -5.1(1.4)].……An increase in PCDI from 5500 to 9800 Chinese Yuan was associated with a larger single-exposure risk measure when other determinants were at a lower level [posterior mean and SD: -43.4 (8.1)], and an increase in antenatal care rate from 85.7% to 95.9% was associated with a higher response in the decrease in maternal mortality when other determinants were at a higher level [posterior mean and SD: -21.4(9.6)] (Fig. S4).”

**Comment 9:**

**The term "interquartile range" is used incorrectly; the IQR specifically refers to the difference between Q1 and Q3, and so I recommend simply reporting [Q1 - Q3] instead.**

Reply:

We have replaced the term “interquartile range” in the note of Table 1 with “Q1, Q3”.

**Comment 10:**

**It does not appear that fiscal expenditure is adjusted for inflation - the nominal values reported mean quite different things, and perhaps would look less stark if adjusted to, say, 2010 RMB.**

Reply:

Thank you for your comment. We have conducted the sensitivity analysis considering the inflation and added the methods and results as follows:

1. In the Methods (Page 12 Lines 259-263):

“2) Considering the potential impact of inflation on the analytical results, we conducted the BKMR analysis with government health expenditure adjusted for inflation. Government health expenditure was adjusted using 2012 as the base year for reference and the yearly inflation in the International Financial Statistics database [18].”

Reference: 18.	International Monetary Fund. Inflation, consumer prices (annual %) - China. World Bank Group. 2020. Available from: https://data.worldbank.org/indicator/FP.CPI.TOTL.ZG?end=2020&locations=CN&start=2004&view=chart.

2. In the Results (Page 17 Lines 368-371):

“The results of BKMR analysis with government health expenditure adjusted for inflation, are shown in Figs. S7-S11 and Tables S7-S10. The results of GroupPIP, CondPIP, and single-exposure risk measures did not change substantially.”

**Fig. S7 Determinants of total and cause-specific maternal mortality in eastern and western China during 2004-2012 and 2013-2020, using Bayesian Kernel Machine Regression hierarchical variable selection with fiscal expenditure adjusted for inflation.**

Note: The asterisk * and dagger † symbols in the cell represent that the determinant contributes the most to the exposure-response relationship when all other determinants are fixed at their 25^th^ and 75^th^ percentiles, respectively. White cell represents that the determinant is not identified as the most important determinant of maternal mortality. CMD, coexisting medical diseases; HDP, hypertensive disorders in pregnancy; PCDI, per capita disposable income.

**Table S7 Group and conditional posterior inclusion probabilities for each determinant in Eastern China, 2004-2012, using Bayesian Kernel Machine Regression hierarchical variable selection with fiscal expenditure adjusted for inflation.**

| **Exposure** | **Exposure group** | **Total maternal mortality** | | **Maternal mortality due to hemorrhage** | | **Maternal mortality due to coexisting medical diseases** | | **Maternal mortality due to hypertensive disorders in pregnancy** | |
| --- | --- | --- | --- | --- | --- | --- | --- | --- | --- |
|  |  | **GroupPIP** | **CondPIP** | **GroupPIP** | **CondPIP** | **GroupPIP** | **CondPIP** | **GroupPIP** | **CondPIP** |
| Hospital delivery rate | 1 | 1 | 1 | 1 | 1 | 1 | 0.767 | 1 | 0.860 |
| Antenatal care rate | 1 | 1 | 0 | 1 | 0 | 1 | 0.001 | 1 | 0.140 |
| Prenatal booking rate | 1 | 1 | 0 | 1 | 0 | 1 | 0.233 | 1 | 0 |
| Local fiscal expenditure on healthcare | 2 | 0.997 | 1 | 1 | 1 | 0.807 | 1 | 0.928 | 1 |
| Urbanization rate | 3 | 1 | 1 | 1 | 1 | 0.992 | 0.836 | 0.844 | 0.370 |
| PCDI | 3 | 1 | 0 | 1 | 0 | 0.992 | 0.164 | 0.844 | 0.617 |
| Average years of schooling for females | 3 | 1 | 0 | 1 | 0 | 0.992 | 0 | 0.844 | 0.013 |
| Number of Ob/Gyn beds per 1000 livebirths | 4 | 0.881 | 0.046 | 0.917 | 0.025 | 0.641 | 0.727 | 0.735 | 0.939 |
| Number of MCH personnel per 1000 livebirths | 4 | 0.881 | 0.953 | 0.917 | 0.974 | 0.641 | 0.272 | 0.735 | 0.061 |

Note: GroupPIP, group posterior inclusion probabilities; CondPIP, conditional posterior inclusion probabilities; MCH, maternal and child health; Ob/Gyn, obstetrics and gynecology; PCDI, per capita disposable income.

**Table S8 Group and conditional posterior inclusion probabilities for each determinant in Western China, 2004-2012, using Bayesian Kernel Machine Regression hierarchical variable selection with fiscal expenditure adjusted for inflation.**

| **Exposure** | **Exposure group** | **Total maternal mortality** | | **Maternal mortality due to hemorrhage** | | **Maternal mortality due to coexisting medical diseases** | | **Maternal mortality due to hypertensive disorders in pregnancy** | |
| --- | --- | --- | --- | --- | --- | --- | --- | --- | --- |
|  |  | **GroupPIP** | **CondPIP** | **GroupPIP** | **CondPIP** | **GroupPIP** | **CondPIP** | **GroupPIP** | **CondPIP** |
| Hospital delivery rate | 1 | 1 | 0.012 | 1 | 0.186 | 1 | 0.994 | 1 | 0 |
| Antenatal care rate | 1 | 1 | 0.949 | 1 | 0.722 | 1 | 0.005 | 1 | 1 |
| Prenatal booking rate | 1 | 1 | 0.040 | 1 | 0.098 | 1 | 0.001 | 1 | 0 |
| Local fiscal expenditure on healthcare | 2 | 0.37 | 1 | 0.302 | 1 | 0.406 | 1 | 0.020 | 1 |
| Urbanization rate | 3 | 1 | 0 | 0.957 | 0.006 | 0.533 | 0.243 | 0.121 | 0.061 |
| PCDI | 3 | 1 | 1 | 0.957 | 0.944 | 0.533 | 0.104 | 0.121 | 0.876 |
| Average years of schooling for females | 3 | 1 | 0 | 0.957 | 0.050 | 0.533 | 0.652 | 0.121 | 0.063 |
| Number of Ob/Gyn beds per 1000 livebirths | 4 | 0.637 | 0.364 | 0.406 | 0.407 | 0.335 | 0.551 | 0.040 | 0.218 |
| Number of MCH personnel per 1000 livebirths | 4 | 0.637 | 0.636 | 0.406 | 0.592 | 0.335 | 0.449 | 0.040 | 0.782 |

Note: GroupPIP, group posterior inclusion probabilities; CondPIP, conditional posterior inclusion probabilities; MCH, maternal and child health; Ob/Gyn, obstetrics and gynecology; PCDI, per capita disposable income.

**Table S9 Group and conditional posterior inclusion probabilities for each determinant in Eastern China, 2013-2020, using Bayesian Kernel Machine Regression hierarchical variable selection with fiscal expenditure adjusted for inflation.**

| **Exposure** | **Exposure group** | **Total maternal mortality** | | **Maternal mortality due to hemorrhage** | | **Maternal mortality due to coexisting medical diseases** | | **Maternal mortality due to hypertensive disorders in pregnancy** | |
| --- | --- | --- | --- | --- | --- | --- | --- | --- | --- |
|  |  | **GroupPIP** | **CondPIP** | **GroupPIP** | **CondPIP** | **GroupPIP** | **CondPIP** | **GroupPIP** | **CondPIP** |
| Hospital delivery rate | 1 | 1 | 1 | 1 | 1 | 0.710 | 0.772 | 0.909 | 0.085 |
| Antenatal care rate | 1 | 1 | 0 | 1 | 0 | 0.710 | 0.183 | 0.909 | 0.897 |
| Prenatal booking rate | 1 | 1 | 0 | 1 | 0 | 0.710 | 0.045 | 0.909 | 0.017 |
| Local fiscal expenditure on healthcare | 2 | 0.663 | 1 | 0.488 | 1 | 0.999 | 1 | 0.644 | 1 |
| Urbanization rate | 3 | 0.996 | 0.781 | 1 | 0.996 | 0.990 | 0.684 | 0.992 | 0.325 |
| PCDI | 3 | 0.996 | 0.205 | 1 | 0.004 | 0.990 | 0.300 | 0.992 | 0.652 |
| Average years of schooling for females | 3 | 0.996 | 0.014 | 1 | 0 | 0.990 | 0.016 | 0.992 | 0.022 |
| Number of Ob/Gyn beds per 1000 livebirths | 4 | 0.330 | 0.548 | 0.571 | 0.136 | 0.711 | 0.399 | 0.972 | 0.995 |
| Number of MCH personnel per 1000 livebirths | 4 | 0.330 | 0.452 | 0.571 | 0.864 | 0.711 | 0.601 | 0.972 | 0.005 |

Note: GroupPIP, group posterior inclusion probabilities; CondPIP, conditional posterior inclusion probabilities; MCH, maternal and child health; Ob/Gyn, obstetrics and gynecology; PCDI, per capita disposable income.

**Table S10 Group and conditional posterior inclusion probabilities for each determinant in Western China, 2013-2020, using Bayesian Kernel Machine Regression hierarchical variable selection with fiscal expenditure adjusted for inflation.**

| **Exposure** | **Exposure group** | **Total maternal mortality** | | **Maternal mortality due to hemorrhage** | | **Maternal mortality due to coexisting medical diseases** | | **Maternal mortality due to hypertensive disorders in pregnancy** | |
| --- | --- | --- | --- | --- | --- | --- | --- | --- | --- |
|  |  | **GroupPIP** | **CondPIP** | **GroupPIP** | **CondPIP** | **GroupPIP** | **CondPIP** | **GroupPIP** | **CondPIP** |
| Hospital delivery rate | 1 | 1 | 0 | 1 | 0.977 | 0.962 | 0.026 | 1 | 0 |
| Antenatal care rate | 1 | 1 | 1 | 1 | 0.023 | 0.962 | 0.902 | 1 | 0 |
| Prenatal booking rate | 1 | 1 | 0 | 1 | 0 | 0.962 | 0.072 | 1 | 1 |
| Local fiscal expenditure on healthcare | 2 | 0.773 | 1 | 0.268 | 1 | 0.673 | 1 | 0.360 | 1 |
| Urbanization rate | 3 | 1 | 0 | 1 | 0 | 0.899 | 0.088 | 0.480 | 0.229 |
| PCDI | 3 | 1 | 0 | 1 | 0.449 | 0.899 | 0.020 | 0.480 | 0.491 |
| Average years of schooling for females | 3 | 1 | 1 | 1 | 0.551 | 0.899 | 0.891 | 0.480 | 0.232 |
| Number of Ob/Gyn beds per 1000 livebirths | 4 | 0.832 | 0.040 | 0.487 | 0.700 | 0.473 | 0.638 | 0.705 | 0.662 |
| Number of MCH personnel per 1000 livebirths | 4 | 0.832 | 0.960 | 0.487 | 0.300 | 0.473 | 0.362 | 0.705 | 0.338 |

Note: GroupPIP, group posterior inclusion probabilities; CondPIP, conditional posterior inclusion probabilities; MCH, maternal and child health; Ob/Gyn, obstetrics and gynecology; PCDI, per capita disposable income.

**Fig. S8 Single-exposure risk measures for each determinant in Eastern China, 2004-2012, using Bayesian Kernel Machine Regression hierarchical variable selection with fiscal expenditure adjusted for inflation.**

Note: The plot compares the exposure-response relationships associated with a change in a single exposure from the 75^th^ percentile to the 25^th^ percentile, when the other exposures are fixed at their 25th, 50th, and 75th percentiles. MCH, maternal and child health; Ob/Gyn, obstetrics and gynecology; PCDI, per capita disposable income; HDP, hypertensive disorders in pregnancy; q.fixed, quantiles at which to fix the remaining exposures.

**Fig. S9 Single-exposure risk measures for each determinant in Western China, 2004-2012, using Bayesian Kernel Machine Regression hierarchical variable selection with fiscal expenditure adjusted for inflation.**

Note: The plot compares the exposure-response relationships associated with a change in a single exposure from the 75^th^ percentile to the 25^th^ percentile, when the other exposures are fixed at their 25th, 50th, and 75th percentiles. MCH, maternal and child health; Ob/Gyn, obstetrics and gynecology; PCDI, per capita disposable income; HDP, hypertensive disorders in pregnancy; q.fixed, quantiles at which to fix the remaining exposures.

**Fig. S10 Single-exposure risk measures for each determinant in Eastern China, 2013-2020, using Bayesian Kernel Machine Regression hierarchical variable selection with fiscal expenditure adjusted for inflation.**

Note: The plot compares the exposure-response relationships associated with a change in a single exposure from the 75^th^ percentile to the 25^th^ percentile, when the other exposures are fixed at their 25th, 50th, and 75th percentiles. MCH, maternal and child health; Ob/Gyn, obstetrics and gynecology; PCDI, per capita disposable income; HDP, hypertensive disorders in pregnancy; q.fixed, quantiles at which to fix the remaining exposures.

**Fig. S11 Single-exposure risk measures for each determinant in Western China, 2013-2020, using Bayesian Kernel Machine Regression hierarchical variable selection with fiscal expenditure adjusted for inflation.**

Note: The plot compares the exposure-response relationships associated with a change in a single exposure from the 75^th^ percentile to the 25^th^ percentile, when the other exposures are fixed at their 25th, 50th, and 75th percentiles. MCH, maternal and child health; Ob/Gyn, obstetrics and gynecology; PCDI, per capita disposable income; HDP, hypertensive disorders in pregnancy; q.fixed, quantiles at which to fix the remaining exposures.

**Comment 11:**

**Finally, the text in the figures were quite hard to read - please ensure that they are available at sufficient DPI in the final submission.** 

Reply:

We have submitted figures with sufficient DPI as separate figure files.

***Comments from Reviewer 2:*** 

**Summary
This manuscript analyzes maternal mortality in China from 2004-2020, using provincial-level data and Bayesian kernel machine regression (BKMR) to evaluate the relative contributions of social and health system determinants. The authors highlight hospital delivery, antenatal care, health financing, income, and urbanization as key factors behind China's remarkable reduction in maternal mortality, and frame lessons for low- and middle-income countries (LMICs) still striving toward SDG target 3.1.
The topic is timely and highly relevant for global health policy. The innovative application of BKMR to maternal health research is a clear methodological strength, and the East/West and pre-/post-2013 comparisons are well thought out. However, the manuscript would benefit from sharpening its narrative focus, simplifying presentation of results, and expanding the discussion of policy implications — particularly around quality of care. In addition, the quality of data collected from the national statistics especial in the 2010s may be problematic.**

Reply:

Thank you for your positive comments and suggestions regarding the narrative focus, presentation of results, discussion and data source. Please see our point-by-point responses below.

**Comment 1:**

**The manuscript has put overemphasis on descriptive results, which have been widely reported over the past decade. For example, Figures 1-3 and much of the Results reiterate well-documented declines in maternal mortality. These sections could be streamlined to allow more focus on the determinants analysis, which is the paper's main value-added.** 

Reply:

Thank you for your suggestion. We have streamlined the descriptive results by condensing the two sections on "Trends in maternal mortality in eastern and western China from 2004 to 2020" and "Cause-specific maternal mortality in eastern and western China from 2004 to 2020" into a single section as follows (Pages 12-14 Lines 273-293):

“**Trends in maternal mortality in eastern and western China from 2004 to 2020**

In both the East and West, maternal mortality declined substantially, and the urban-rural gap in maternal mortality also narrowed between 2004 and 2020 (Fig. 1). However, regional differences in maternal mortality persisted between the East and West during this period (Fig. S2). The characteristics of social and health system-related determinants in the East and West before and after 2013 are shown in Table 1.

Based on the data from the GBD 2021 study, maternal hemorrhage, indirect maternal deaths, other direct maternal disorders and maternal hypertensive disorders remained the leading causes of maternal mortality in China from 2004 to 2020 (Fig. 2). We further utilized provincial-level data from the National Health Statistics Yearbooks to estimate the cause-specific mortality rates in the East and West. Due to the lack of a detailed classification of maternal disorders in the provincial-level data comparable to that in the GBD study, we focused on hemorrhage, coexisting medical conditions, and HDP in the following analysis. These major causes of maternal mortality have been steadily declining in both the East and West from 2004 to 2020 (Fig. 3).”

**Comment 2:**

**The study used many data from the national annual statistics books published by the Chinese central and local governments. However, we all know that the quality of such routine data has been problematic. For example, China has got a huge population of rural-to-urban migrant workers (over 200 million, based on some reports). The health statistics in one province may not include those migrant populations who do not have official resident status. Such a practice was very common in the early 2000s in China. I am wondering how the authors addressed such a challenge. In addition, different provinces/regions may have different working definition of "urbanization rate". Is the indicator only the use of official residence status? Or the calculation has considered the rural-to-urban population living in cities, but registered in the rural areas?**

Reply:

During the past several years, several national information systems for maternal and child health have been established in China, including the National Maternal and Child Health Statistics System and Maternal and Child Health Surveillance System. Strict reporting and quality control procedures are implemented from the village to the national level in these systems. All pregnant women residing within the designated surveillance area, irrespective of their household registration status, are included as subjects of monitoring.

The calculation of social determinants, such as urbanization rate and per capita disposable income, was based on data from the resident population. The resident population consists of individuals who reside in the same place as their household registration, as well as those who reside in one place but have their household registration elsewhere. Therefore, the statistics include the migrant population that does not have official resident status, and the urbanization rate here has considered the rural-to-urban population living in cities, but registered in the rural areas.

**Comment 3:**
**It should also give clearer interpretation of BKMR Findings: while GroupPIP and CondPIP are presented, their practical meaning is not sufficiently explained. For a broad PLOS Medicine readership, please clarify: what does a CondPIP of 0.9 mean in policy terms? How large are these effects compared to known biomedical interventions?**

Reply:

We have added the following explanation in the Methods and Discussion to elucidate the meaning of GroupPIP and CondPIP:

1. Methods (Page 11 Lines 233-239):

“A smaller GroupPIP indicates that this group is less likely to be associated with the outcome and is a less important component in the mixture of the 4 groups. In contrast, a larger GroupPIP suggests that this group is relatively more important than other groups. Similarly, a larger ConPIP suggests that the determinant is more likely to be associated with the outcome within the group, making it a more important component within the group. Conversely, a smaller CondPIP indicates that the determinant is relatively less important.”

2. Discussion (Page 21 Lines 485-490):

“For LMICs with a high burden of maternal mortality, lessons could be learnt from China’s remarkable success. Here, we identified the most important components in the mixture of determinants in order to suggest the priorities that can be considered in policy and program planning. This is particularly relevant for resource-limited settings, where simultaneous optimization of multiple determinants may not be feasible.”

As mentioned in the Introduction (Pages 5-6 Lines 82-96) and Methods (Page 7 Lines 120-123), we focused on the non-biomedical determinants and selected the social and health system-related determinants based on a recent review on the distal, non-biomedical determinants of maternal mortality. Proximate factors of maternal mortality, such as biomedical interventions, fall outside the scope of our analytical framework. Moreover, we are unable to obtain provincial data on biomedical interventions in China from 2004 to 2020. Therefore, we were unable to compare the effects of social and health system-related determinants with those of biomedical interventions.

**Comment 4:**
**The discussion highlights antenatal care and facility births, but the issue of quality of care is underdeveloped. WHO has emphasized that coverage alone is insufficient; over-medicalization (e.g., rising cesarean section rates in China) and quality deficits are important considerations. A more balanced discussion would strengthen the global relevance.**

Reply:

Thank you for your valuable comments. We have revised the Discussion to highlight the quality of care as follows (Pages 18-19 Lines 421-437):

“It should also be noted that without ensuring adequate quality of care, increased coverage of maternal care does not necessarily improve maternal survival. Increasing the proportion of women delivering in medical facilities is estimated to reduce total maternal death, death from hypertensive disorders and from hemorrhage by merely 29%, 29% and 53%, respectively. Instead, an integrated strategy to simultaneously increase facility births, improve the availability of clinical services and quality of care at facilities, and improve linkages to care could substantially improve maternal health outcomes and reduce total maternal death, death from hypertensive disorders and from hemorrhage by 51%, 60% and 79%, respectively. In addition to the recommendation on the number of antenatal visits, the WHO also emphasizes interventions to improve the quality of antenatal services in LMICs.

In China, a comprehensive strategy consisting of multidimensional interventions was employed in the Reducing Maternal Mortality and Eliminating Neonatal Tetanus Program to improve both the quantity and quality of maternal care and in-hospital delivery……”

Indeed, over-medicalization has already emerged in China, such as too many antenatal care visits and rising cesarean section rates. Excessive medicalization has also been observed in LMICs, where too little, too late and too much, too soon are found to coexist. However, our data did not include information related to over-medicalization, and our results cannot support the discussion of this phenomenon. Therefore, given the importance of over-medicalization, we have acknowledged and briefly addressed it in the limitations sections of the Discussion, as follows (Page 23 Lines 538-542):

“Fourth, both too much, too soon and too little, too late are associated with maternal mortality and have been found to coexist in China and other LMICs. However, due to the lack of data reflecting over-medicalization, we were unable to examine the impact of insufficient and excessive medicalization on maternal mortality in China in the past decades.”

**Comment 5:**
**Abstract: Too dense. Simplify by foregrounding the five main determinants identified (hospital delivery, antenatal care, financing, urbanization, income) and reduce descriptive statistics.**

Reply:

We have simplified the Abstract as follows, and the Abstract has also been revised according to Comment 6 from Reviewer 1.

“**Background** Globally, maternal mortality is off track in achieving the Sustainable Development Goals by 2030. Over the past two decades, China has dramatically reduced maternal mortality in more developed (eastern) and less developed (western) regions. An understanding of the social and health system determinants of maternal mortality in China may be helpful for countries attempting to meet the 2030 targets and beyond.

**Methods and findings** We analyzed provincial-level data on maternal mortality and social and health system determinants from the National Health Statistics Yearbooks and China Statistical Yearbooks from 2004 to 2020. We investigated the determinants of maternal mortality before and after 2013, the year that a historic national program, Reducing Maternal Mortality and Eliminating Neonatal Tetanus, came to an end. Bayesian kernel machine regression was employed to analyze social and health system determinants as a mixture and identify relatively important determinants for the total and cause-specific maternal mortality. Hospital delivery rate, antenatal care rate, urbanization rate, local fiscal expenditure on healthcare, and per capita disposable income (PCDI) were identified as important determinants negatively associated with maternal mortality during the 2004-2020 period. In the East, hospital delivery correlated with the decreases in total maternal mortality and maternal deaths due to hemorrhage and hypertensive disorders before 2013, and the urbanization rate was negatively associated with total and hemorrhage-related maternal mortality after 2013. Local fiscal expenditure on healthcare and antenatal care were also important determinants in the East. In the West, the antenatal care rate was a relatively important determinant for reducing total maternal mortality before and after 2013. PCDI and hospital delivery were also important determinants in the West.

**Conclusions** Coverage of maternal care, health financing and urbanization were important determinants associated with the substantial reduction in maternal deaths in eastern and western China during 2004-2020. The improvement of the quantity and quality of antenatal care and hospital delivery may be a viable policy priority in less developed regions worldwide.”

**Comment 6:**
**Figures: Figure 4 is central but overloaded. Consider splitting into separate panels (total vs. cause-specific mortality) for clarity.**

Reply:

We have split Figure 4 into four panels as follows:

**Fig. 4.** Determinants of (A) total maternal mortality, (B) maternal mortality due to hemorrhage, (C) maternal mortality due to coexisting medical diseases, and (D) maternal mortality due to hypertensive disorders in pregnancy in eastern and western China during 2004-2012 and 2013-2020. The asterisk * and dagger † symbols in the cell represent that the determinant contributes the most to the exposure-response relationship when all other determinants are fixed at their 25th and 75th percentiles, respectively. White cell represents that the determinant is not identified as the most important determinant of maternal mortality. PCDI, per capita disposable income.

**Comment 7:**
**Terminology: Ensure consistent use of "maternal mortality ratio (MMR)" vs. "maternal mortality" throughout. Clarify terms like "coexisting medical conditions" vs. "indirect causes."**

Reply:

We have used “maternal mortality” throughout the manuscript. We have added the following sentences in the Methods to clarify terms like “coexisting medical conditions” vs. “indirect causes” (Page 7 Lines 137-140, Page 8 Lines 149-153):

“In the National Health Statistical Yearbook, the causes of maternal deaths were only classified as hemorrhage, coexisting medical conditions, hypertensive disorders in pregnancy (HDP), amniotic fluid embolism, and others……Indirect maternal deaths are due to existing diseases that are exacerbated by pregnancy. Coexisting medical conditions recorded in the National Health Statistical Yearbook specifically refer to internal medicine diseases that develop during pregnancy or pre-exist and are aggravated by pregnancy.”

**Comment 8:**
**Discussion Structure: Could be tightened by reducing repetition and focusing on three big global lessons (coverage + quality; system/urbanization context).**

Reply:

Thank you for your suggestion. We have revised the Discussion to reduce repetition and focus on three big global lessons. The second paragraph of the Discussion was removed to avoid repetition with the Introduction. The paragraphs discussing China’s experience in the Reducing Maternal Mortality and Eliminating Neonatal Tetanus Program have also been revised to focus more on coverage and quality, while also emphasizing the quality of care in line with your Comment 4 (Pages 17-20 Lines 389-468). Basic information on the Program was moved from the Discussion to the Methods (Page 10 Lines 213-222).

***Comments from Reviewer 3:*** 

**The study aimed to estimate the most contributing social determinants and health system-related factors to the decline in total and cause-specific maternal mortality in China from 2004 to 2020, by use of Bayesian kernel machine regression (BKMR) to tackle the collinearity of covariates. The data originated from Global Burden of Diseases, Injuries, and Risk Factors Study 2021 (GBD 2021), the National Health Statistics Yearbooks, and the China Statistical Yearbooks. This study is of significant importance. It will provide important experience for other regions to achieving MMR decline target in SDGs. Overall, the manuscript is well written. I just have minor comments.**

Reply:

Thank you for your positive comments. Please see our point-by-point responses below.

**Comment 1:**
**1.The dependent variable in this study is time-dependent. Please explain how this issue is addressed in statistical analysis.**

Reply:

We assigned an identifier to each province and set the “id” in the kmbayes() function as the vector of identifiers, allowing for the correlation between time-dependent repeated measures within the same province to be included. We have explained how this issue was addressed in the Methods (Pages 10-11 Lines 225-227):

“Each province was assigned an identifier, so that a random intercept was included in the model to account for the correlation between repeated measures within the same province.”

**Comment 2:**
**2.Have authors tried to analyze the contributing determinants by rural and urban areas?**

Reply:

Thank you for your suggestion. We acknowledge the importance of analyzing the contributing determinants in rural and urban areas. However, the analysis is limited by the unavailability of rural and urban data at the provincial level for most of the determinants. We added the following sentences in the Discussion to state this limitation (Page 23 Lines 535-538):

“Third, data on rural and urban areas at the provincial level are unavailable for most of the determinants. Therefore, we are not able to analyze the contributing determinants by rural and urban areas in the East and West.”

***Technical requests:*** 

**1) Please amend your detailed Financial Disclosure statement. This is published with the article. It must therefore be completed in full sentences and contain the exact wording you wish to be published.
1. State the initials, alongside each funding source, of each author to receive each grant.
2. Since the funders had no role in your study, please state: “The funders had no role in study design, data collection and analysis, decision to publish, or preparation of the manuscript.”**

Reply:

We have amended our Financial Disclosure statement as follows (Page 24 Lines 580-584):

“This study was supported in part by the Shanghai Sailing Program (24YF2750300) and the Postdoctoral Fellowship Program of China Postdoctoral Science Foundation (GZC20251538) (X.Z.) and the Gates Foundation (INV-088623) (J.Z.). The funders had no role in study design, data collection and analysis, decision to publish, or preparation of the manuscript.”

**2) We ask that a manuscript source file is provided at Revision. Please upload your manuscript file as a .doc, .docx, .rtf or .tex.**

Reply:

We have uploaded the manuscript file as a .docx. **3) Please upload the main figures as separate figure files in .tif or .eps format. For more information about figure files please see our guidelines: 
https://journals.plos.org/plosmedicine/s/figures  
https://journals.plos.org/plosmedicine/s/figures#loc-file-requirements**  

Reply:

We have uploaded the main figures as separate figure files in .tif format.

**4) We have noticed that you have referred to Appendix 12 in your manuscript. However, there is no corresponding file uploaded to the submission. Please upload it separately with the item type 'Supporting Information'.**

Reply:

We have uploaded all the original Appendices separately with the item type “Supporting Information”. **5) We notice that your supplementary figures and tables (Appendix p 1-11) are included in the manuscript file. Please remove them and upload them with the file type 'Supporting Information'. Please ensure that each Supporting Information file has a legend listed in the manuscript after the references list.**

Reply:

We have removed the original Appendices, uploaded them with the file type “Supporting Information”, and listed a legend for each Supporting information file after the references list. **6) Some material included in your submission may be copyrighted. According to PLOS’s copyright policy, authors who use figures or other material (e.g., graphics, clipart, maps) from another author or copyright holder must demonstrate or obtain permission to publish this material under the Creative Commons Attribution 4.0 International (CC BY 4.0) License used by PLOS journals. Please closely review the details of PLOS’s copyright requirements here: PLOS Licenses and Copyright. If you need to request permissions from a copyright holder, you may use PLOS's Copyright Content Permission form.

Please respond directly to this email and provide any known details concerning your material's license terms and permissions required for reuse, even if you have not yet obtained copyright permissions or are unsure of your material's copyright compatibility. Once you have responded and addressed all other outstanding technical requirements, you may resubmit your manuscript within Editorial Manager. 

Potential Copyright Issues:

i) Suppl 3: please (a) provide a direct link to the base layer of the map (i.e., the country or region border shape) and ensure this is also included in the figure legend; and (b) provide a link to the terms of use / license information for the base layer image or shapefile. We cannot publish proprietary or copyrighted maps (e.g. Google Maps, Mapquest) and the terms of use for your map base layer must be compatible with our CC BY 4.0 license. 

Note: if you created the map in a software program like R or ArcGIS, please locate and indicate the source of the basemap shapefile onto which data has been plotted.

If your map was obtained from a copyrighted source please amend the figure so that the base map used is from an openly available source. Alternatively, please provide explicit written permission from the copyright holder granting you the right to publish the material under our CC BY 4.0 license.

If you are unsure whether you can use a map or not, please do reach out and we will be able to help you. The following websites are good examples of where you can source open access or public domain maps: 
* U.S. Geological Survey (USGS) - All maps are in the public domain. (http://www.usgs.gov) 
* PlaniGlobe - All maps are published under a Creative Commons license so please cite “PlaniGlobe, http://www.planiglobe.com, CC BY 2.0” in the image credit after the caption. (http://www.planiglobe.com/?lang=enl) 
* Natural Earth - All maps are public domain. (http://www.naturalearthdata.com/about/terms-of-use/)**

Reply:

The map was created using R software, based on openly available administrative boundary shapefiles from the Tianditu platform (National Platform for Common Geospatial Information Services). The source of the basemap is: <https://cloudcenter.tianditu.gov.cn/administrativeDivision>. The map data are official public resources, with survey approval number GS(2024)0650, and therefore do not involve copyright restrictions incompatible with the CC BY 4.0 license. We have provide a link to the base layer of the map in the figure legend as follows: “Note: The source of the basemap is: <https://cloudcenter.tianditu.gov.cn/administrativeDivision>.”


***Editorial requests:*

 (Note: not all will apply to your paper, but please check each item carefully)**

*** Please include an Author Summary after the Abstract. See:https://journals.plos.org/plosmedicine/s/revising-your-manuscript**

Reply:

We have included an Author Summary after the Abstract as follows (Pages 3-4 Lines 35-58):

“**Author Summary**

**Why was this study done?**

•	Maternal mortality worldwide is not decreasing fast enough to reach global targets by 2030.

•	China has made major progress in reducing maternal deaths in both wealthier and poorer regions over the past two decades.

•	Learning from China’s experience may help low- and lower-middle-income countries accelerate progress in reducing maternal mortality toward global goals.

**What did the researchers do and find?**

•	We employed a Bayesian kernel machine regression model to identify important social and health system determinants associated with decreased maternal mortality in China using nationwide provincial-level data from 2004 to 2020.

•	Hospital delivery rate, antenatal care rate, urbanization rate, local fiscal expenditure on healthcare, and per capita disposable income were associated with the remarkable progress in maternal survival in both wealthier and poorer regions in China.

**What do these findings mean?**

•	To reduce maternal deaths in less developed regions, it is vital to increase both the utilization and quality of antenatal care and facility birth, supported by adequate health funding and social improvements.

•	The study’s main limitation was the unavailability of more direct and comprehensive indicators of non-biomedical determinants.”

***        We ask every co-author listed on the manuscript to fill in a contributing author statement, making sure to declare all competing interests. If any of the co-authors have not filled in the statement, we will remind them to do so when the paper is revised. If all statements are not completed in a timely fashion this could hold up the re-review process. If new competing interests are declared later in the revision process, this may also hold up the submission. Should there be a problem getting one of your co-authors to fill in a statement we will be in contact. Please do not add or remove authors without first discussing this with the handling editor. You can see our competing interests policy here:** [**http://journals.plos.org/plosmedicine/s/competing-interests**](http://journals.plos.org/plosmedicine/s/competing-interests)**.**

Reply:

Every co-author will fill in the contributing author statement in the revision process.

***        Please upload any figures associated with your paper as individual TIF or EPS files with 300dpi resolution at resubmission; please read our figure guidelines for more information on our requirements: http://journals.plos.org/plosmedicine/s/figures. While revising your submission, we strongly recommend that you use PLOS's NAAS tool (https://ngplosjournals.pagemajik.ai/artanalysis) to test your figure files. NAAS can convert your figure files to the TIFF file type and meet basic requirements (such as print size, resolution), or provide you with a report on issues that do not meet our requirements and that NAAS cannot fix.**

**After uploading your figures to PLOS's NAAS tool - https://ngplosjournals.pagemajik.ai/artanalysis, NAAS will process the files provided and display the results in the "Uploaded Files" section of the page as the processing is complete.
If the uploaded figures meet our requirements (or NAAS is able to fix the files to meet our requirements), the figure will be marked as "fixed" above. If NAAS is unable to fix the files, a red "failed" label will appear above.
When NAAS has confirmed that the figure files meet our requirements, please download the file via the download option, and include these NAAS processed figure files when submitting your revised manuscript.**

Reply:

We have uploaded the all figures as separate figure files in .tif format with 300dpi resolution.

***        Please ensure that the paper adheres to the PLOS Data Availability Policy (see http://journals.plos.org/plosmedicine/s/data-availability), which requires that all data underlying the study's findings be provided in a repository or as Supporting Information. For data residing with a third party, authors are required to provide instructions with contact information (web or email address) for obtaining the data. Please note that a study author cannot be the contact person for the data. PLOS journals do not allow statements supported by "data not shown" or "unpublished results." For such statements, authors must provide supporting data or cite public sources that include it.**

Reply:

We have included a Data Availability Statement as follows (Page 24 Lines 568-573): “Data is publicly available at the National Health Commission website (http://www.nhc.gov.cn/mohwsbwstjxxzx/tjzxtjcbw/tjsj_list.shtml), National Bureau of Statistics website (https://www.stats.gov.cn/sj/ndsj/), and Global Health Data Exchange (GHDx) online website (<https://vizhub.healthdata.org/gbd-results/>). All data inputs for BKMR are also provided in Table S13.”

***        We expect all researchers with submissions to PLOS in which author-generated code underpins the findings in the manuscript to make all author-generated code available without restrictions upon publication of the work. In cases where code is central to the manuscript, we may require the code to be made available as a condition of publication. Authors are responsible for ensuring that the code is reusable and well documented. Please make any custom code available, either as part of your data deposition or as a supplementary file. Please add a sentence to your data availability statement regarding any code used in the study, e.g. "The code used in the analysis is available from Github [URL] and archived in Zenodo [DOI link]" Please review our guidelines at https://journals.plos.org/plosmedicine/s/materials-software-and-code-sharing and ensure that your code is shared in a way that follows best practice and facilitates reproducibility and reuse. Because Github depositions can be readily changed or deleted, we encourage you to make a permanent DOI'd copy (e.g. in Zenodo) and provide the URL.** 

Reply:

We have added the following sentences to the Data Availability Statement: “No custom code was used in this study. We used open-source R software for these analyses. R scripts use existing code examples from publicly available R packages and have been described in the Methods.” (Page 24 Lines 573-576)

***        Please ensure that the study is reported according to the appropriate guideline (e.g. STROBE) and include the completed checklist as Supporting Information. When completing the checklist, please use section and paragraph numbers, rather than page numbers. Please add the following statement, or similar, to the Methods: "This study is reported as per [XXXX] guideline (S1 Checklist)."**

Reply:

We have completed a GATHER checklist and uploaded it as Supporting Information. We also added a statement in the Methods: “This study is reported as per the Guidelines for Accurate and Transparent Health Estimates Reporting (GATHER) statement (Checklist S1).” (Page 12 Lines 269-271) **FORMATTING - GENERAL
*        Abstract: Please structure your abstract using the PLOS Medicine headings (Background, Methods and Findings, Conclusions). Please combine the Methods and Findings sections into one section.**

Reply:

We have structured the abstract using the PLOS Medicine headings. ***        At this stage, we ask that you include a short, non-technical Author Summary of your research to make findings accessible to a wide audience that includes both scientists and non-scientists. The Author Summary should immediately follow the Abstract in your revised manuscript. This text is subject to editorial change and should be distinct from the scientific abstract. Ideally each sub-heading should contain 2-3 single sentence, concise bullet points containing the most salient points from your study. In the final bullet point of 'What Do These Findings Mean?', please include the main limitations of the study in non-technical language. Please see our author guidelines for more information: https://journals.plos.org/plosmedicine/s/revising-your-manuscript#loc-author-summary.**

Reply:

We have included an Author Summary after the Abstract as follows (Pages 3-4 Lines 35-58):

“**Author Summary**

**Why was this study done?**

•	Maternal mortality worldwide is not decreasing fast enough to reach global targets by 2030.

•	China has made major progress in reducing maternal deaths in both wealthier and poorer regions over the past two decades.

•	Learning from China’s experience may help low- and lower-middle-income countries accelerate progress in reducing maternal mortality toward global goals.

**What did the researchers do and find?**

•	We employed a Bayesian kernel machine regression model to identify important social and health system determinants associated with decreased maternal mortality in China using nationwide provincial-level data from 2004 to 2020.

•	Hospital delivery rate, antenatal care rate, urbanization rate, local fiscal expenditure on healthcare, and per capita disposable income were associated with the remarkable progress in maternal survival in both wealthier and poorer regions in China.

**What do these findings mean?**

•	To reduce maternal deaths in less developed regions, it is vital to increase both the utilization and quality of antenatal care and facility birth, supported by adequate health funding and social improvements.

•	The study’s main limitation was the unavailability of more direct and comprehensive indicators of non-biomedical determinants.”

***        Please express the main results with 95% CIs as well as p values. When reporting p values please report as p<0.001 and where higher as the exact p value p=0.002, for example. Throughout, suggest reporting statistical information as follows to improve clarity for the reader "22% (95% CI [13%,28%]; p</=)". Please be sure to define all numerical values at first use.**

Reply:

Under the Bayesian approach, we have reported the posterior inclusion probability, posterior mean and standard deviation.

***        Please include page numbers and line numbers in the manuscript file. Use continuous line numbers (do not restart the numbering on each page).** 

Reply:

We have included page numbers and line numbers in the manuscript file.

***        Please cite the reference numbers in square brackets. Citations should precede punctuation.**

Reply:

We have cited the reference numbers in square brackets preceding punctuation.

**FIGURES AND TABLES
*        Please provide titles and legends for all figures and tables (including those in Supporting Information files).**

Reply:

We have provided the titles and legends for all figures and tables after References.

***        Please define all abbreviations used in each figure/table (including those in Supporting Information files).**

Reply:

We have defined all abbreviations used in each figure/table.

***        Please consider avoiding the use of red and green in order to make your figure more accessible to those with color blindness.**

Reply:

We have changed the colors in Fig. 2, Fig. 4, and figures in Supporting Information.

**SUPPLEMENTARY MATERIAL
*        Please note that supplementary material will be posted as supplied by the authors. Therefore, please amend it according to the relevant comments outlined here.**

Reply:

We have amended the supplementary material according to the comments below.

***        Please cite your Supporting Information as outlined here: https://journals.plos.org/plosmedicine/s/supporting-information**

Reply:

We have cited the Supporting Information as outlined on https://journals.plos.org/plosmedicine/s/supporting-information.

**REFERENCES
*        PLOS uses the numbered citation (citation-sequence) method and first six authors, et al.**

Reply:

The References have been revised using the PLoS Style.

***        Please ensure that journal name abbreviations match those found in the National Center for Biotechnology Information (NCBI) databases (http://www.ncbi.nlm.nih.gov/nlmcatalog/journals), and are appropriately formatted and capitalised.**

Reply:

The journal name abbreviations match those found in the NCBI databases, and are appropriately formatted and capitalized.

***        Where website addresses are cited, please include the complete URL and specify the date of access (e.g. [accessed: 12/06/2023]).**

Reply:

The URL and data of access has been added where website addresses are cited.

***        Please also see https://journals.plos.org/plosmedicine/s/submission-guidelines#loc-references for further details on reference formatting.**

Reply:

The References have been revised using the PLoS Style.

**OBSERVATIONAL STUDIES

*        Abstract: Please include the study design, population and setting, number of participants, years during which the study took place (enrollment and follow up), length of follow up, and main outcome measures.**

Reply:

Not applicable.

***        Please ensure that the study is reported according to the STROBE (or appropriate STOBE extension) guideline (available from: https://www.equator-network.org/reporting-guidelines/strobe) and include the completed STROBE (or STROBE extension) checklist as Supporting Information. Please add the following statement, or similar, to the Methods: "This study is reported as per the Strengthening the Reporting of Observational Studies in Epidemiology (STROBE) guideline (S1 Checklist)." When completing the checklist, please use section and paragraph numbers, rather than page numbers.** 

Reply:

Not applicable.

***        [FOR POPULATION HEALTH/REGISTRY STUDIES] Please ensure that the study is reported according to the RECORD guideline (available from https://www.record-statement.org) and include the completed checklist as Supporting Information. Please add the following statement, or similar, to the Methods: "This study is reported as per the Reporting of Studies Conducted using Observational Routinely-Collected Data (RECORD) guideline (S1 Checklist)." When completing the checklist, please use section and paragraph numbers, rather than page numbers.**

Reply:

Not applicable.

***        [FOR POPULATION HEALTH ESTIMATES] Please ensure that the study is reported according to the GATHER statement (available from https://www.equator-network.org/reporting-guidelines/gather-statement) and include the completed checklist as Supporting Information. Please add the following statement, or similar, to the Methods: "This study is reported as per the Guidelines for Accurate and Transparent Health Estimates Reporting (GATHER) statement (S1 Checklist)." When completing the checklist, please use section and paragraph numbers, rather than page numbers.** 

Reply:

We have completed a GATHER checklist and uploaded it as Supporting Information. We also added a statement in the Methods: “This study is reported as per the Guidelines for Accurate and Transparent Health Estimates Reporting (GATHER) statement (Checklist S1).” (Page 12 Lines 269-271) ***        [FOR MEDIATION ANALYSES] We recommend that the study is reported according to the AGReMA statement (https://agrema-statement.org/#:~:text=AGReMA%20is%20an%20evidence%2D%20and,randomised%20trials%20and%20observational%20studies) and include the completed checklist as Supporting Information. Please add the following statement, or similar, to the Methods: "This study is reported as per the Guideline for Reporting Mediation Analyses (AGReMA) statement (S1 Checklist)." When completing the checklist, please use section and paragraph numbers, rather than page numbers.** 

Reply:

Not applicable.

***        For all observational studies, in the manuscript text, please indicate: (1) the specific hypotheses you intended to test, (2) the analytical methods by which you planned to test them, (3) the analyses you actually performed, and (4) when reported analyses differ from those that were planned, transparent explanations for differences that affect the reliability of the study's results. If a reported analysis was performed based on an interesting but unanticipated pattern in the data, please be clear that the analysis was data driven.**

Reply:

Not applicable.

***        Please state in the Methods section whether the study had a prospective protocol or analysis plan. If a prospective analysis plan (from your funding proposal, IRB or other ethics committee submission, study protocol, or other planning document written before analyzing the data) was used in designing the study, please include the relevant document(s) with your revised manuscript as a Supporting Information file to be published alongside your study and cite it in the Methods section. A legend for this file should be included at the end of your manuscript. If no such document exists, please make sure that the Methods section transparently describes when analyses were planned, and when/why any data-driven changes to analyses took place. Changes in the analysis, including those made in response to peer review comments, should be identified as such in the Methods section of the paper, with rationale.**

Reply:

Not applicable.

**MODELLING STUDIES

The following list is derived from Geoffrey P Garnett, Simon Cousens, Timothy B Hallett, Richard Steketee, Neff Walker. Mathematical models in the evaluation of health programmes. (2011) Lancet DOI:10.1016/S0140-6736(10)61505-X:        
                
*        If pertinent, please provide a diagram that shows the model structure, including how the natural history of the disease is represented, the process and determinants of disease acquisition, and how the putative intervention could affect the system.**

Reply:

Not applicable.

***        Please provide a complete list of model parameters, including clear and precise descriptions of the meaning of each parameter, together with the values or ranges for each, with justification or the primary source cited and important caveats about the use of these values noted.**

Reply:

Not applicable.

***        Please provide a clear statement about how the model was fitted to the data, including goodness-of-fit measure, the numerical algorithm used, which parameter varied, constraints imposed on parameter values, and starting conditions.**

Reply:

Not applicable.

***        For uncertainty analyses, please state the sources of uncertainties quantified and not quantified [can include parameter, data, and model structure].**

Reply:

Not applicable.

***        Please provide sensitivity analyses to identify which parameter values are most important in the model. Uncertainty estimates seek to derive a range of credible results on the basis of an exploration of the range of reasonable parameter values. The choice of method should be presented and justified.**

Reply:

Not applicable.

***        Please discuss the scientific rationale for the choice of model structure and identify points where this choice could influence conclusions drawn. Please also describe the strength of the scientific basis underlying the key model assumptions.**

Reply:

Not applicable.

***        For studies that develop a prediction model or evaluate its performance, please ensure that the study is reported according to the TRIPOD statement (https://www.equator-network.org/reporting-guidelines/tripod-statement) and include the completed checklist as Supporting Information. Please add the following statement, or similar, to the Methods: "This study is reported as per the Transparent Reporting of a Multivariable Prediction Model for Individual Prognosis Or Diagnosis (TRIPOD) statement (S1 Checklist)." For studies using machine learning, please use the TRIPOD-AI checklist. When completing the checklist, please use section and paragraph numbers, rather than page numbers.**

Reply:

Not applicable.
