# Supplementary material for: Social and health system factors associated with maternal mortality in Eastern and Western China: Population health estimates using provincial-level data
Source: PLoS Med. 2025 Dec 4;22(12):e1004837. doi: 10.1371/journal.pmed.1004837 (PMC12677549; doi:10.1371/journal.pmed.1004837)
Supplement: Attachment — Submitted filename: 20251112 Response letter.docx [file pmed.1004837.s033.docx]

Dear editor,

Thank you for the reviewers’ and editors’ comments concerning our manuscript entitled “Social and Health System Determinants of Maternal Mortality in More and Less Developed Regions of China: Implications for SDG 2030”. They are very helpful for improving our paper. We have carefully reviewed them and thoroughly revised the paper. The following is our point-by-point response to the reviewers’ comments and editorial requests.

***Requests from Editors:*** 

**Request 1: Please note in particular that we require that you to remove all causal assertions from the manuscript and frame your results as associative, as per the comments of reviewer 1. Please remove 'important' from the Abstract, qualify determinants (i.e. determinants of what) and see below for other Abstract requirements.**

Reply:

We have removed all causal assertions from the manuscript, including the Abstract, main text and the legends for figures, tables and supporting information, and framed our results as associative. Please see our reply to the comments of Reviewer 1 for details. In the Abstract, the word “important” has been removed, the word “determinant” has been replaced with “factor” and has been qualified. We also revised the Abstract according to other requirements below. Please refer to our response to Requests 5, 8, 10, and 11 for details.

**Request 2: Please also note that we require you to respond to the comments of reviewer 2, and please refer to this reviewer's comments from the original round of review, and provide a response in full to the points originally raised.**

Reply:

We have provided a response to the comments of Reviewer 2. Please refer to our response to the comments of Reviewer 2 for details.

**Request 3: Please ensure that the manuscript includes a definition of Eastern and Western China.**

Reply:

We have included a definition of Eastern and Western China in the Methods as follows (Page 9-10 Lines 195-204):

“We used the Aihui-Tengchong line as the geographical dividing line proposed by the Chinese geographer Huanyong Hu to divide China into eastern and western regions based on population density, natural conditions, and social, economic and human activities. Maternal mortality in the East and West was calculated as the weighted average of maternal mortality in the 24 eastern provinces (Anhui, Beijing, Chongqing, Fujian, Guangdong, Guangxi, Guizhou, Hainan, Hebei, Heilongjiang, Henan, Hubei, Hunan, Jiangsu, Jiangxi, Jilin, Liaoning, Shandong, Shanghai, Shanxi, Sichuan, Tianjin, Yunnan, Zhejiang) and 7 western provinces (Gansu, Inner Mongolia, Ningxia, Qinghai, Shaanxi, Tibet, Xinjiang), respectively.”

**Request 4: Please confirm that your title complies with PLOS Medicine's style. Your title must be nondeclarative and not a question. It should begin with main concept if possible. "Effect of" should be used only if causality can be inferred, i.e., for an RCT. Please place the study design ("A randomized controlled trial," "A retrospective study," "A modelling study," etc.) in the subtitle (ie, after a colon).** 

Reply:

We have revised the title as “Social and health system factors associated with maternal mortality in more and less developed regions of China: Population health estimates using provincial-level data” to comply with PLOS Medicine’s style and the requirement of changing causal assertions to associative ones.

**Request 5: Please confirm that your abstract complies with our requirements, including format (three sections: Background, Methods and Findings, and Conclusions) and providing all the information relevant to this study type https://journals.plos.org/plosmedicine/s/submission-guidelines#loc-abstract** 

Reply:

We have revised the Abstract as follows, including format and providing all the information relevant to the study type (Pages 2-3 Lines 2-38):

“**Background** Globally, maternal mortality is off track in achieving the Sustainable Development Goals by 2030. Over the past two decades, China has dramatically reduced maternal mortality in more developed (eastern) and less developed (western) regions. An understanding of the social and health system factors associated with maternal mortality in China may be helpful for countries attempting to meet the 2030 targets and beyond.

**Methods and Findings** We analyzed provincial-level data on maternal mortality and social and health system factors from the National Health Statistics Yearbooks and China Statistical Yearbooks from 2004 to 2020. We investigated the factors associated with maternal mortality before and after 2013, the year that a historic national program, Reducing Maternal Mortality and Eliminating Neonatal Tetanus, came to an end. Bayesian kernel machine regression was employed to analyze social and health system factors (urbanization rate, per capita disposable income, average years of schooling, number of health technical personnel in maternal and child health care, number of hospital beds for obstetrics and gynecology, local fiscal expenditure on healthcare, prenatal booking rate, antenatal care rate, and hospital delivery rate) as a mixture and identify the factors with larger posterior inclusion probability and a higher value of the exposure-response relationship for the total and cause-specific maternal mortality. In the East, an increase in hospital delivery rate correlated with the decreases in total maternal mortality [posterior mean and standard deviation (SD): -14.8(1.5)] before 2013, and the urbanization rate was negatively associated with total maternal mortality [posterior mean and SD: -3.9(0.6)] after 2013. Hospital delivery, urbanization, local fiscal expenditure on healthcare and antenatal care were the factors associated with reduced cause-specific maternal mortality in the East. In the West, an increase in antenatal care rate was associated with reduced total maternal mortality, with the posterior mean and SD of -33.8(6.8) and -11.5(4.1) before and after 2013, respectively. Hospital delivery and antenatal care were the factors associated with reduced cause-specific maternal mortality in the West. The main limitation of this study was the data constraints in the national statistics.

**Conclusions** Coverage of maternal care, health financing and urbanization were the factors associated with the substantial reduction in maternal deaths in eastern and western China during 2004-2020. The improvement of the quantity and quality of antenatal care and hospital delivery may be a viable policy priority in less developed regions worldwide.”

**Request 6: Please ensure that the Introduction ends with a clear description of the study question or hypothesis.** 

Reply:

We have revised the Introduction so that it ends with a clear description of the study question (Pages 6-7 Lines 118-127):

“We aimed to estimate the posterior inclusion probability (PIP) and the single-exposure risk measures of 9 social and health system factors, including urbanization rate, per capita disposable income (PCDI), average years of schooling, number of health technical personnel in maternal and child health (MCH), number of hospital beds for obstetrics and gynecology (Ob/Gyn), local fiscal expenditure on healthcare, prenatal booking rate, antenatal care rate, and hospital delivery rate, and identify the factors with higher values of exposure-response relationships for reduced maternal mortality in China, thereby drawing implications for LMICs around the world.”

**Request 7: Please ensure that all abbreviations are defined at first use throughout the text.** 

Reply:

We have ensured that all abbreviations are defined at first use throughout the text.

**Request 8: Please confirm that all numbers presented in the abstract are present and identical to numbers presented in the main manuscript text.** 

Reply:

We confirmed that all numbers presented in the Abstract are present and identical to numbers given in the main text.

**Request 9: Please review your text for claims of novelty or primacy (e.g. 'for the first time') and remove this language. In addition, please check that any use of statistical terms (such as trend or significant) are supported by the data, and if not please remove them.** 

Reply:

We confirmed that claims of novelty or primacy are not included, and any use of statistical terms is supported by the data.

**Request 10: In the last sentence of the Abstract Methods and Findings section, please describe the main limitation(s) of the study's methodology.** 

Reply:

We have described the main limitation of the study in the Abstract Methods and Findings section as follows (Page 3 Lines 32-33): “The main limitation of this study was the data constraints in the national statistics.”

**Request 11: In the abstract, please include the important dependent variables that are adjusted for in the analyses**. 

Reply:

A total of 9 variables were included in the Bayesian kernel machine regression, and we did not adjust for other variables in the main analyses. To clarify this, we have revised the Abstract as follows (Page 2 Lines 13-21): “Bayesian kernel machine regression was employed to analyze social and health system factors (urbanization rate, per capita disposable income, average years of schooling, number of health technical personnel in maternal and child health care, number of hospital beds for obstetrics and gynecology, local fiscal expenditure on healthcare, prenatal booking rate, antenatal care rate, and hospital delivery rate) as a mixture and identify the factors with larger posterior inclusion probability and a higher value of the exposure-response relationship for the total and cause-specific maternal mortality.”

**Request 12: Please convert any stacked bar charts to another data representation for example a table, or other type of graph. If that is not possible, please explain why and provide a Table containing the data in the bar chart (e.g. in Fig. 2). 
In Fig. 3, please lighten the dark blue colour (Hemorrhage) for improved clarity.** 

Reply:

We used stacked bar charts because they are well-suited for displaying data with many categories, such as the different causes for maternal mortality in our study. They allow clear comparison of both the rate and the relative contribution of each cause across years. We have provided the tables for Fig. 2 in the supporting information (Table S2 and Table S3) as follows:

**Table S2 Cause-specific maternal mortality rate in China, 2004-2020.**

| **Year** | **Maternal hemorrhage** | **Indirect maternal deaths** | **Other direct maternal disorders** | **Maternal hypertensive disorders** | **Maternal obstructed labor and uterine rupture** | **Late maternal deaths** | **Ectopic pregnancy** | **Maternal sepsis and other maternal infections** | **Maternal abortion and miscarriage** | **Maternal deaths aggravated by HIV/AIDS** |
| --- | --- | --- | --- | --- | --- | --- | --- | --- | --- | --- |
| 2004 | 0.34 | 0.31 | 0.14 | 0.13 | 0.06 | 0.03 | 0.03 | 0.03 | 0.01 | 0.00 |
| 2005 | 0.29 | 0.28 | 0.13 | 0.12 | 0.05 | 0.03 | 0.03 | 0.03 | 0.01 | 0.00 |
| 2006 | 0.25 | 0.25 | 0.12 | 0.10 | 0.05 | 0.03 | 0.03 | 0.02 | 0.01 | 0.00 |
| 2007 | 0.21 | 0.23 | 0.11 | 0.09 | 0.04 | 0.03 | 0.03 | 0.02 | 0.01 | 0.00 |
| 2008 | 0.19 | 0.21 | 0.10 | 0.09 | 0.04 | 0.03 | 0.03 | 0.02 | 0.01 | 0.00 |
| 2009 | 0.16 | 0.19 | 0.10 | 0.08 | 0.03 | 0.03 | 0.03 | 0.01 | 0.01 | 0.00 |
| 2010 | 0.13 | 0.16 | 0.08 | 0.06 | 0.03 | 0.03 | 0.02 | 0.01 | 0.01 | 0.00 |
| 2011 | 0.11 | 0.14 | 0.07 | 0.06 | 0.02 | 0.03 | 0.02 | 0.01 | 0.01 | 0.00 |
| 2012 | 0.10 | 0.13 | 0.07 | 0.05 | 0.02 | 0.03 | 0.02 | 0.01 | 0.01 | 0.00 |
| 2013 | 0.09 | 0.12 | 0.06 | 0.05 | 0.02 | 0.03 | 0.02 | 0.01 | 0.01 | 0.00 |
| 2014 | 0.08 | 0.11 | 0.06 | 0.05 | 0.02 | 0.02 | 0.02 | 0.01 | 0.01 | 0.00 |
| 2015 | 0.08 | 0.11 | 0.06 | 0.05 | 0.02 | 0.03 | 0.02 | 0.01 | 0.01 | 0.00 |
| 2016 | 0.08 | 0.11 | 0.06 | 0.05 | 0.02 | 0.03 | 0.02 | 0.01 | 0.01 | 0.00 |
| 2017 | 0.07 | 0.10 | 0.06 | 0.04 | 0.02 | 0.03 | 0.02 | 0.01 | 0.00 | 0.00 |
| 2018 | 0.06 | 0.09 | 0.05 | 0.04 | 0.02 | 0.02 | 0.02 | 0.01 | 0.00 | 0.00 |
| 2019 | 0.05 | 0.08 | 0.05 | 0.03 | 0.02 | 0.02 | 0.01 | 0.01 | 0.00 | 0.00 |
| 2020 | 0.04 | 0.07 | 0.04 | 0.03 | 0.01 | 0.02 | 0.01 | 0.00 | 0.00 | 0.00 |

**Table S3 Cause-specific maternal mortality fractions in China, 2004-2020.**

| **Year** | **Maternal hemorrhage** | **Indirect maternal deaths** | **Other direct maternal disorders** | **Maternal hypertensive disorders** | **Maternal obstructed labor and uterine rupture** | **Late maternal deaths** | **Ectopic pregnancy** | **Maternal sepsis and other maternal infections** | **Maternal abortion and miscarriage** | **Maternal deaths aggravated by HIV/AIDS** |
| --- | --- | --- | --- | --- | --- | --- | --- | --- | --- | --- |
| 2004 | 31.2 | 28.2 | 13.2 | 11.9 | 5.2 | 3.2 | 3.1 | 2.8 | 1.3 | 0.0 |
| 2005 | 29.9 | 28.5 | 13.7 | 11.9 | 5.2 | 3.5 | 3.3 | 2.7 | 1.3 | 0.0 |
| 2006 | 28.8 | 28.7 | 14.1 | 12.0 | 5.2 | 3.8 | 3.5 | 2.5 | 1.4 | 0.0 |
| 2007 | 27.5 | 29.1 | 14.5 | 12.1 | 5.2 | 4.1 | 3.7 | 2.4 | 1.4 | 0.0 |
| 2008 | 26.4 | 29.5 | 14.8 | 12.1 | 5.1 | 4.5 | 3.9 | 2.3 | 1.4 | 0.0 |
| 2009 | 25.0 | 29.9 | 15.1 | 12.2 | 5.1 | 4.9 | 4.1 | 2.3 | 1.4 | 0.0 |
| 2010 | 24.1 | 30.1 | 15.3 | 12.2 | 5.1 | 5.2 | 4.3 | 2.2 | 1.4 | 0.0 |
| 2011 | 23.4 | 30.2 | 15.5 | 12.2 | 5.2 | 5.6 | 4.4 | 2.2 | 1.4 | 0.0 |
| 2012 | 22.7 | 30.1 | 15.7 | 12.2 | 5.4 | 5.9 | 4.4 | 2.2 | 1.4 | 0.0 |
| 2013 | 22.0 | 30.0 | 15.9 | 12.2 | 5.6 | 6.2 | 4.5 | 2.2 | 1.4 | 0.0 |
| 2014 | 21.3 | 30.0 | 16.1 | 12.2 | 5.8 | 6.6 | 4.6 | 2.1 | 1.4 | 0.0 |
| 2015 | 20.7 | 30.0 | 16.2 | 12.2 | 5.9 | 6.9 | 4.7 | 2.1 | 1.4 | 0.0 |
| 2016 | 20.2 | 29.9 | 16.4 | 12.2 | 6.0 | 7.2 | 4.7 | 2.1 | 1.4 | 0.0 |
| 2017 | 19.5 | 30.0 | 16.6 | 12.0 | 6.1 | 7.5 | 4.9 | 2.1 | 1.3 | 0.0 |
| 2018 | 18.9 | 30.1 | 16.9 | 12.0 | 6.2 | 7.9 | 4.9 | 2.0 | 1.3 | 0.0 |
| 2019 | 18.3 | 30.2 | 17.1 | 11.8 | 6.2 | 8.2 | 5.0 | 1.9 | 1.3 | 0.0 |
| 2020 | 17.8 | 30.3 | 17.3 | 11.6 | 6.3 | 8.6 | 5.0 | 1.8 | 1.3 | 0.0 |

In Fig. 3, we have lightened the dark blue color (Hemorrhage) as follows:

**Fig. 3** Cause-specific maternal mortality rate for hemorrhage, coexisting medical diseases, and HDP in eastern and western China, 2004-2020. HDP, hypertensive disorders in pregnancy.

**Request 13: Where data points are discrete, please ensure that they are depicted in the figures as discrete data and not as a continuous line, e.g., in Fig. 1.** 

Reply:

We have revised Fig. 1 as follows:

**Fig. 1** Trends in maternal mortality in eastern and western China during 2004-2020. The dashed line represents the year 2013 when the Reducing Maternal Mortality and Eliminating Neonatal Tetanus Program (2000-2013) ended.

**Request 14: Please provide the unadjusted comparisons as well as the adjusted comparisons in all relevant Tables.** 

Reply:

For the main and sensitivity analyses using the Bayesian kernel machine regression (BKMR), all the nine social and health system factors, including urbanization rate, per capita disposable income, average years of schooling, number of health technical personnel in maternal and child health, number of hospital beds for obstetrics and gynecology, local fiscal expenditure on healthcare, prenatal booking rate, antenatal care rate, and hospital delivery rate, were included in the model. We did not adjust for other variables in the BKMR analyses.

For the sensitivity analyses using linear mixed-effects models, we have provided the unadjusted and adjusted comparisons in the Methods, Results, and supporting information (Table S8-S9):

1. Methods (Page 12 Lines 263-267): “The effect of each determinant was estimated using univariate and multivariate linear mixed-effects models weighted by the number of livebirths in each province for each year. In the multivariate mixed-effects models, the other eight social and health system factors were adjusted for when the effect of each factor was calculated.”

2. Results (Page 17 Lines 368-370): “Tables S8-S9 show the results of linear mixed-effects models for important components in the mixture of determinants identified by BKMR, including hospital delivery rate, antenatal care rate, urbanization rate, local fiscal expenditure on healthcare, and PCDI.”

3. Table S8

**Table S8 Effects of determinants on maternal mortality, using univariate linear mixed-effects models weighted by the number of livebirths.**

| **Outcome** | **Determinant** | **2004-2012** | | **2013-2020** | |
| --- | --- | --- | --- | --- | --- |
|  |  | **East** | **West** | **East** | **West** |
| Total maternal mortality | Hospital delivery rate | -1.51 (-1.62, -1.41) | -2.37 (-2.66, -2.09) | -4.42 (-5.34, -3.5) | -6.95 (-8.06, -5.84) |
|  | Antenatal care rate | -2.37 (-2.77, -1.97) | -6.30 (-7.15, -5.45) | -1.16 (-1.52, -0.81) | -1.66 (-2.62, -0.70) |
|  | Local fiscal expenditure on healthcare | -0.07 (-0.08, -0.06) | -0.28 (-0.35, -0.21) | -0.01 (-0.01, 0) | -0.07 (-0.11, -0.03) |
|  | Urbanization rate | -0.95 (-1.06, -0.84) | -2.13 (-2.86, -1.39) | -0.33 (-0.40, -0.25) | -1.28 (-1.78, -0.79) |
|  | Per capita disposable income | -21.1 (-24.2, -18.0) | -59.8 (-73.8, -45.8) | -2.13 (-2.72, -1.54) | -10.4 (-15.2, -5.47) |
| Maternal mortality due to hemorrhage | Hospital delivery rate | -1.07 (-1.13, -1.01) | -1.31 (-1.48, -1.15) | -2.81 (-3.16, -2.46) | -3.76 (-4.23, -3.29) |
|  | Antenatal care rate | -1.47 (-1.76, -1.19) | -3.37 (-3.78, -2.96) | -0.46 (-0.63, -0.29) | -1.96 (-2.33, -1.59) |
|  | Local fiscal expenditure on healthcare | -0.04 (-0.05, -0.04) | -0.15 (-0.19, -0.11) | 0 (0, 0) | -0.04 (-0.06, -0.01) |
|  | Urbanization rate | -0.61 (-0.69, -0.54) | -1.28 (-1.67, -0.90) | -0.16 (-0.19, -0.12) | -0.73 (-1, -0.46) |
|  | Per capita disposable income | -13.0 (-15.2, -10.7) | -32.9 (-40.8, -25.0) | -0.99 (-1.26, -0.72) | -5.51 (-8.44, -2.58) |
| Maternal mortality due to coexisting medical diseases | Hospital delivery rate | -0.31 (-0.35, -0.27) | -0.62 (-0.72, -0.52) | -0.76 (-1.21, -0.32) | -0.69 (-1.09, -0.3) |
|  | Antenatal care rate | -0.52 (-0.63, -0.42) | -1.44 (-1.77, -1.11) | -0.23 (-0.37, -0.08) | -0.16 (-0.4, 0.07) |
|  | Local fiscal expenditure on healthcare | -0.02 (-0.02, -0.01) | -0.07 (-0.1, -0.05) | 0 (0, 0) | -0.01 (-0.02, 0) |
|  | Urbanization rate | -0.23 (-0.26, -0.2) | -0.53 (-0.75, -0.31) | -0.06 (-0.09, -0.03) | -0.16 (-0.29, -0.04) |
|  | Per capita disposable income | -5.16 (-5.92, -4.40) | -15.8 (-20.3, -11.4) | -0.54 (-0.77, -0.31) | -0.83 (-2.21, 0.55) |
| Maternal mortality due to hypertensive disorders in pregnancy | Hospital delivery rate | -0.16 (-0.17, -0.14) | -0.53 (-0.65, -0.41) | -0.39 (-0.59, -0.18) | -1.17 (-1.5, -0.84) |
|  | Antenatal care rate | -0.26 (-0.31, -0.21) | -1.47 (-1.74, -1.21) | -0.13 (-0.2, -0.06) | -0.41 (-0.63, -0.19) |
|  | Local fiscal expenditure on healthcare | -0.01 (-0.01, -0.01) | -0.05 (-0.08, -0.03) | 0 (0, 0) | -0.01 (-0.02, 0) |
|  | Urbanization rate | -0.1 (-0.12, -0.09) | -0.39 (-0.62, -0.17) | -0.03 (-0.04, -0.02) | -0.23 (-0.36, -0.11) |
|  | Per capita disposable income | -2.33 (-2.7, -1.95) | -11.7 (-16.5, -6.96) | -0.27 (-0.38, -0.16) | -1.77 (-3.11, -0.43) |

Note: Effects are shown as estimates and the 95% confidence intervals in the linear mixed effects models.

4. Table S9

**Table S9 Effects of determinants on maternal mortality, using multivariate linear mixed-effects models weighted by the number of livebirths.**

| **Outcome** | **Determinant** | **2004-2012** | | **2013-2020** | |
| --- | --- | --- | --- | --- | --- |
|  |  | **East** | **West** | **East** | **West** |
| Total maternal mortality | Hospital delivery rate | -1.09 (-1.22, -0.95) | -1.94 (-2.56, -1.31) | -3.09 (-4.05, -2.13) | -4.93 (-6.57, -3.29) |
|  | Antenatal care rate | -0.05 (-0.46, 0.36) | -1.90 (-3.97, 0.17) | -0.64 (-1.00, -0.29) | -0.96 (-1.97, 0.05) |
|  | Local fiscal expenditure on healthcare | -0.01 (-0.02, 0.01) | 0.07 (-0.07, 0.22) | 0 (0, 0.01) | -0.02 (-0.06, 0.02) |
|  | Urbanization rate | -0.27 (-0.42, -0.13) | 0.98 (0.22, 1.74) | -0.16 (-0.31, -0.01) | -0.60 (-1.47, 0.27) |
|  | Per capita disposable income | -1.42 (-5.78, 2.94) | -30.5 (-55.9, -5.18) | 0.21 (-1.05, 1.46) | 6.88 (-2.27, 16.02) |
| Maternal mortality due to hemorrhage | Hospital delivery rate | -0.90 (-0.99, -0.82) | -0.86 (-1.22, -0.5) | -2.16 (-2.52, -1.08) | -2.78 (-3.54, -2.02) |
|  | Antenatal care rate | -0.02 (-0.27, 0.23) | -2.01 (-3.11, -0.92) | -0.17 (-0.3, -0.04) | -1.05 (-1.61, -0.49) |
|  | Local fiscal expenditure on healthcare | 0 (-0.01, 0) | 0.04 (-0.03, 0.1) | 0 (0, 0) | -0.01 (-0.02, 0) |
|  | Urbanization rate | -0.14 (-0.23, -0.06) | -0.01 (-0.44, 0.43) | -0.11 (-0.16, -0.06) | -0.36 (-0.64, -0.08) |
|  | Per capita disposable income | 1.11 (-1.58, 3.79) | -3.4 (-18.0, 11.1) | 0.56 (0.11, 1.01) | 3.57 (-0.35, 7.49) |
| Maternal mortality due to coexisting medical diseases | Hospital delivery rate | -0.16 (-0.21, -0.11) | -0.59 (-0.80, -0.37) | -0.39 (-0.87, 0.08) | -0.58 (-1.20, 0.04) |
|  | Antenatal care rate | 0.09 (-0.05, 0.22) | -0.37 (-1.04, 0.3) | -0.04 (-0.21, 0.12) | 0.01 (-0.4, 0.42) |
|  | Local fiscal expenditure on healthcare | 0 (-0.01, 0) | 0 (-0.05, 0.05) | 0 (0, 0) | -0.02 (-0.03, -0.01) |
|  | Urbanization rate | -0.10 (-0.15, -0.06) | 0.48 (0.22, 0.75) | -0.03 (-0.09, 0.03) | -0.27 (-0.54, 0) |
|  | Per capita disposable income | -0.45 (-1.95, 1.05) | -8.51 (-17.4, 0.41) | -0.13 (-0.66, 0.41) | 5.14 (1.77, 8.52) |
| Maternal mortality due to hypertensive disorders in pregnancy | Hospital delivery rate | -0.10 (-0.12, -0.08) | -0.38 (-0.63, -0.12) | -0.14 (-0.36, 0.07) | -1.13 (-1.71, -0.55) |
|  | Antenatal care rate | 0.02 (-0.04, 0.08) | -1.05 (-1.83, -0.26) | -0.08 (-0.16, -0.01) | -0.12 (-0.52, 0.27) |
|  | Local fiscal expenditure on healthcare | 0 (0, 0) | 0.05 (0, 0.1) | 0 (0, 0) | 0 (-0.01, 0.01) |
|  | Urbanization rate | -0.03 (-0.05, -0.01) | 0.26 (-0.05, 0.57) | -0.03 (-0.05, 0) | -0.06 (-0.31, 0.18) |
|  | Per capita disposable income | 0.01 (-0.66, 0.68) | -7.63 (-18.2, 2.91) | -0.04 (-0.29, 0.21) | 1.45 (-1.69, 4.59) |

Note: Effects are shown as estimates and the 95% confidence intervals in the linear mixed effects model, adjusting for the other eight determinants. For example, the effect of hospital delivery rate was estimated when urbanization rate, per capita disposable income, average years of schooling, number of health technical personnel in maternal and child health, number of hospital beds for obstetrics and gynecology, local fiscal expenditure on healthcare, prenatal booking rate, and antenatal care rate were adjusted for.

**Request 15: Please specify the variables controlled for in all relevant Tables**

Reply:

We have specified the variables controlled for in Table S9. Please refer to our response to Request 14 for details.

***Comments from Reviewer #1:*** 

**The authors have comprehensively addressed many of my original concerns and those of other reviewers, particularly regarding some of the additional analyses they present. In general, I am satisfied with these revisions, but did still have two lingering comments - one that I would like to see addressed, and another that might serve merely as a suggestion.**

Reply:

Thank you for your comments. Please see our point-by-point responses below.

**Most importantly, the language used in the model still inappropriately makes causal assertions. for instance, the word "determinant" implies that these social factors are determinants of mortality or health outcome, which is a causal association. Furthermore, the word "important" is being used as a proxy for magnitude of effect, which might not necessarily connote "importance" in terms of the concept we regularly think of. Some of the suggested language is indeed appropriate (e.g., directly stating that something was "associated with a higher value of the exposure-response relationship,"), and so I encourage the authors to use such language throughout, instead of anything that may - incorrectly or not - be construed as implying a causal relationship (e.g., "important determinant for").**

Reply:

We have replaced causal assertions with associative ones throughout the manuscript, as well as in the legends for the supporting information. The word “determinant” has been replaced as “factor” throughout, and we also revised the manuscript as follows:

1. Title

We have replaced the “determinants of maternal mortality” in the title as “factors associated with maternal mortality”.

2. Abstract

In the Abstract, we have replaced the word “determinant” with “factor”. We also removed expressions like “was important determinant for” and used expressions such as “correlated with the decreases in total maternal mortality”, “was negatively associated with total maternal mortality”, or “were the factors associated with reduced cause-specific maternal mortality”. In the Author Summary, we also replaced the expression “important social and health system determinants” with “the social and health system factors associated with”.

3. Methods

We have revised the expression containing “important” as follows:

(Page 11 Lines 242-246) “Both GroupPIP and CondPIP are indices that range from 0 to 1. A larger GroupPIP suggests that this group is relatively more likely to be associated with the outcome than other groups. Similarly, a larger ConPIP suggests that the factor is more likely to be associated with the outcome within the group.”

(Page 11 Lines 251-257) “We used the following criteria to identify the factors associated with decreased maternal mortality: 1) Among the four groups of social and health system factors, we first selected the groups with a GroupPIP ≥ 0.9; 2) For a group with a GroupPIP ≥ 0.9, the factor with the highest CondPIP was selected; 3) For the factor with the highest CondPIP within a group, significant and negative estimate of the single-exposure risk measures should be observed.”

4. Results

(Page 15 Lines 302-306) “**Factors associated with total and cause-specific maternal mortality in eastern and western China**

Using BKMR, we identified five factors associated with the decreased maternal mortality based on the results of GroupPIP, CondPIP, and single-exposure risk measures……”

(Page 16 Lines 336-340) “In the West after 2013, and an increase in antenatal care rate from 94.7% to 97.7% was associated with a larger response when considering the single-exposure risk measures, with a posterior mean and SD of -11.5(4.1) when other factors were at a lower level.”

(Pages 16-17 Lines 341-349) “The reduction of deaths due to hemorrhage was mainly correlated with hospital delivery rate, urbanization rate, and local fiscal expenditure on healthcare in the East during 2004-2012, with hospital delivery rate having a higher single-exposure risk measure, and hospital delivery rate and urbanization rate during 2013-2020, with urbanization rate having a higher single-exposure risk measure in the mixture of determinants. In the West, however, antenatal care rate and hospital delivery rate were the factors with larger GroupPIP and CondPIP, and significant single-exposure risk measures before and after 2013, respectively.”

(Page 17 Lines 351-357) “For the reduction of maternal mortality caused by coexisting medical diseases in the East, the urbanization rate and local fiscal expenditure on healthcare were the factors associated with higher values of the exposure-response relationship before and after 2013, respectively. Before 2013, hospital delivery rate was also a factor associated with the decreased maternal mortality due to coexisting medical diseases in both the East and the West.”

(Page 17 Lines 363-365) “Antenatal care rate was the factor associated with a higher value of the exposure-response relationship in the West during 2004-2012, and in the East during 2013-2020.”

5. Discussion

(Page 18 Lines 388-390) “In this study, we conducted a mixture analysis to quantify the exposure-response relationships of social and health system-related factors and the decline of maternal mortality in eastern and western China from 2004 to 2020.”

(Page 20 Lines 495-497) “Here, we identified the factors associated with higher values of the exposure-response relationship in order to suggest the priorities that can be considered in policy and program planning.”

(Page 23 Lines 521-524) “Furthermore, we utilized mixture analysis to simultaneously estimate the effects of these co-occurring factors, which can address the issue of collinearity and rank the values of exposure-response relationships for these factors.”

(Page 23 Lines 533-535) “A global analysis including more distal factors is warranted to rank the values of exposure-response relationships for these co-occurring contributors to decreased maternal mortality.”

(Page 24 Lines 563-567) “Although the social and health system factors are all crucial to maternal mortality, investigating the values of exposure-response relationships for these factors can provide practical guidance for policy and program planning in these developed regions.”

**As a potential suggestion, I wonder whether the current "sensitivity analyses" might actually serve better as the main analysis. For instance, the mixed model that directly addresses the question "an x increase in y is associated with a z change in w, while adjusting for the other abc..." is directly interpretable and directly addresses research questions of interest regarding the magnitude of relationships. The sensitivity analysis might then be the BKMR, which answers the potential challenge to the analysis of "we know some of these are correlated - what might "get chosen" if we only had to choose a subset of these individual predictors."**

Reply:

Thank you for your suggestion. We would still consider using the BKMR analyses as our main analyses. As we mentioned in the Introduction in Page 6 Lines 111-116, hierarchical BKMR addresses the possibility of collinearity by concurrently estimating both the posterior inclusion probability of groups of highly correlated exposures or exposures from common sources, and the individual components within the group, making it more suitable than a mixed-effects model for complex, co-occurring exposure data such as social and health system factors. Therefore, we used BKMR as the main model to identify factors with larger single-exposure risk measures and then applied mixed-effects models to investigate parameter inference for these factors.

**Regardless, I am satisfied with the response to original review and the revised manuscript. The only real comment I have is on the lingering use of causal language.**

Reply:

Thank you for your positive comments. We have framed our results using associative language.

***Comments from Reviewer #2:*** 

**The authors of the manuscript has addressed most of my comments in the revised version. However, they failed to address one major comment, that is, the quality of data derived from the national health statistics published in the 2010s. The answer to my comment (No 2) was vague and did not show their understanding of potential implications for the use of such poor quality data. I understand that it is a very tough question that may not be easily addressed. Should the authors not be able to address this issue, they should at least put it as one of main limitations of the study.**

Reply:

Thank you for raising the important issue of data quality. We fully acknowledge that routine surveillance data and report data may have limitations, such as the reporting biases and regional inconsistencies in data collection and data quality. Nonetheless, we would like to point out that these national surveillance and report data are valuable and available sources providing comprehensive social, economic and health statistics at the provincial levels over multiple decades for not only ecological studies, but also government decision making and performance evaluation of national intervention programs. It is also worth mentioning that China has made considerable efforts to improve data quality in the national surveillance systems, especially for the information system of maternal and child health, so that key trends can be tracked even if some biases remain [1]. The underlying surveillance systems involve strict reporting and quality control procedures [1-4]. While this may not eliminate all errors and biases, it is, nevertheless, the most comprehensive data source available for us to address our research question. Finally, the national surveillance and report data have been used in studies published in high-impact journals [1,5-8]. That said, we have added the following sentences in the Discussion to acknowledge this limitation (Page 24 Lines 558-561):

“Finally, this study relied on national annual statistics, which may be subject to reporting biases and regional inconsistencies in data collection and data quality. This limitation should be considered when interpreting the results.”

References:

1. Qiao J, Wang Y, Li X, Jiang F, Zhang Y, Ma J, et al. A Lancet Commission on 70 years of women's reproductive, maternal, newborn, child, and adolescent health in China. Lancet. 2021;397(10293):2497-2536.
2. Di J, Yang Q, Jin X. Combating maternal mortality in China: experience, challenges and opportunities. Global Health Journal. 2017;1(3):20-33.
3. Gan X, Hao C, Dong X, Alexander S, Dramaix M, Hu L, et al. Provincial maternal mortality surveillance systems in China. Biomed Res Int. 2014;2014:187896.
4. Feng X, Guo S, Hipgrave D, Zhu J, Zhang L, Song L, et al. China's facility-based birth strategy and neonatal mortality: a population-based epidemiological study. Lancet. 2011;378(9801):1493-500.
5. Gao Y, Zhou H, Singh NS, Powell-Jackson T, Nash S, Yang M, et al. Progress and challenges in maternal health in western China: a Countdown to 2015 national case study. Lancet Glob Health. 2017;5(5):e523-e536.
6. Shi H, Chen L, Wei Y, Chen X, Zhao Y. Improving maternal healthcare further in China at a time of low maternal mortality. BMJ. 2024;386:e078640.
7. Li J, Liang J, Wang J, Ren Z, Yang D, Wang Y, et al. Spatiotemporal trends and ecological determinants in maternal mortality ratios in 2,205 Chinese counties, 2010-2013: A Bayesian modelling analysis. PLoS Med. 2020;17(5):e1003114.
8. Liang J, Li X, Kang C, Wang Y, Kulikoff XR, Coates MM, et al. Maternal mortality ratios in 2852 Chinese counties, 1996-2015, and achievement of Millennium Development Goal 5 in China: a subnational analysis of the Global Burden of Disease Study 2016. Lancet. 2019;393(10168):241-252.
